# Hybrid Molecules Containing Methotrexate, Vitamin D, and Platinum Derivatives: Synthesis, Characterization, *In Vitro* Cytotoxicity, *In Silico* ADME Docking, Molecular Docking and Dynamics

**DOI:** 10.1002/cbdv.202400373

**Published:** 2024-11-07

**Authors:** Zintle Mbese, Mpho Choene, Eric Morifi, M. Nwamadi, Samson Adeyemi, Abel Kolawole Oyebamiji, Adedapo S. Adeyinka, Blassan George, Blessing Atim Aderibigbe

**Affiliations:** ^1^ Department of Chemistry University of Fort Hare Alice Campus 5700 Alice, Eastern Cape South Africa; ^2^ Department of Biochemistry University of Johannesburg Kingsway Campus, Auckland Park 2006 Johannesburg South Africa; ^3^ School of Chemistry Mass Spectrometry Division University of Witwatersrand 2050 Johannesburg South Africa; ^4^ Department of Chemistry University of Johannesburg Auckland Park Campus 2006 Johannesburg South Africa; ^5^ Wits Advanced Drug Delivery Platform Research Unit Department of Pharmacy and Pharmacology School of Therapeutic Science Faculty of Health Sciences University of the Witwatersrand Johannesburg South Africa; ^6^ Department of Chemistry and Industrial Chemistry Bowen University Iwo, Osun State Nigeria; ^7^ Research Centre for Synthesis and Catalysis Department of Chemical Sciences University of Johannesburg Auckland Park 2006 Johannesburg South Africa; ^8^ Laser Research Centre, Faculty of Health Sciences University of Johannesburg Doornfontein Campus 2028 Johannesburg South Africa

**Keywords:** Cytotoxicity, Methotrexate, *In Silico* ADME, Vitamin D, Molecular docking

## Abstract

Designing hybrid‐based drugs is one promising strategy for developing effective anticancer drugs that explore combination therapy to enhance treatment efficacy, overcome the development of drug resistance, and lower treatment duration. Bisphosphonates and Vitamin D are commonly administered drugs for the treatment of bone diseases and the prevention of bone metastases. Platinum‐based and methotrexate are widely used anticancer drugs in clinics. However, their use is hampered by adverse side effects. Hybrid‐based compounds containing either bisphosphonate, vitamin D, platinum‐based, or methotrexate were synthesized and characterized using FTIR, ^1^H‐,^31^P, ^13^C‐NMR, and UHPLC‐HRMS which confirmed their successful synthesis. The hydroxyapatite bone binding assay revealed a promising percentage binding affinity of the bisphosphonate hybrid compounds. *In vitro* cytotoxicity assays on MCF‐7 and HT‐29 cell lines revealed a promising cytotoxic effect of hybrid **19** at 50 and 100 μg/mL on HT‐29 and hybrid **15** on MCF‐7 at 100 μg/mL. Molecular docking and dynamics simulation analysis revealed a binding affinity of −9.70 kcal/mol for hybrid **15** against Human 3 alpha‐hydroxysteroid dehydrogenase type 3, showing its capability to inhibit Human 3 alpha‐hydroxysteroid dehydrogenase type 3. The Swiss ADME, ProTox‐II, GUSAR (General Unrestricted Structure‐Activity Relationships), and molecular docking and dynamics studies revealed that these compounds are promising anticancer compounds.

## Graphical abstract

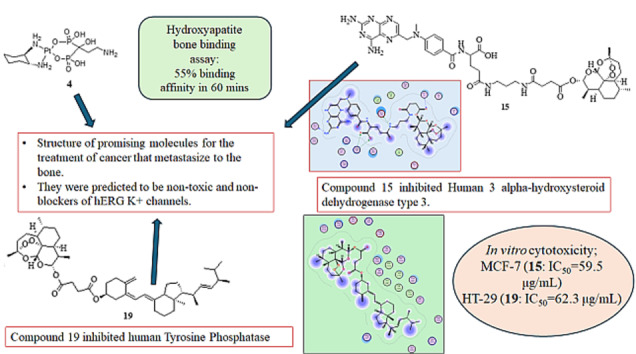

## 1 Introduction

The bone is one of the most frequent sites to which most cancers metastasize, and this is the major cause of death in cancer patients.[^1^, ^2^] There is no cure for bone metastases, and the available treatments are administered to prevent bone metastases, improve the strength of the bones, and reduce pain. Drugs used to prevent bone metastases are bisphosphonates (BPs), vitamin D, demosumab, etc. BPs exert antitumor effects via inhibition of the cycle of increased osteolysis and tumor growth, thereby delaying bone lesion progression.^[3]^ Hybrid compounds containing BPs with promising anticancer activities have been reported.[^4^, ^5^] Vitamin D has also been reported to alter skeletal metastases.^[6]^ Hybrid compounds containing vitamin D have been synthesized and are effective against cancer.[^7^, ^8^]

Inhibiting the progression of metastases and preventing osteoclast activity improves the quality of life in cancer patients.^[9]^ Thus, the development of remedies for the treatment of bone metastases remains a main clinical challenge. Platinum (Pt)‐based drugs are widely used in clinics for the treatment of cancer and are known to induce toxicity, resulting from their non‐specificity, which limits their use for long‐term treatment. Thus, targeted delivery of Pt‐based drugs has been reported to overcome the problems of their non‐specificity.^[10]^ Nadar et al. prepared radioactive Pt‐bisphosphonates (^195^mPt‐BPs) complexes to enable targeted delivery of ^195^mPt to bones of high metabolic activity.^[11]^ Pt‐BP demonstrated a 4.5‐fold higher affinity for bone than the Pt complexes without BPs.^[11]^ On the other hand, Pt‐BP complexes formed fewer Pt‐DNA adducts than the BPs‐free platinum complexes, which indicates that the *in vivo* release of Pt from Pt‐BP complexes will proceed slowly, a feature useful in treating bone metastases with reduced side effects.^[11]^

Methotrexate (MTX) is an anti‐metabolite derived from folic acid and is the most active remedy for t treating several types of cancer.^[12]^ It has also been utilized for the management of acute leukaemia, pulmonary, osteogenic sarcoma, and epidermoid carcinoma.^[13]^ The clinical application of MTX is limited due to its short half‐life, poor solubility, and rapid absorption from the gastrointestinal tract.^[13]^ To prevent and reduce the abovementioned limitations, MTX is administered in combination with other drugs for the treatment of numerous neoplasms, such as acute myeloid leukaemia, bladder cancer, acute lymphoblastic leukaemia, osteosarcomas, meningeal leukaemia, lymphoma, breast cancer, etc. Hybrid compounds containing MTX have been reported to have promising anticancer activities. Kmiecik et al. synthesized MTX‐epirubicin conjugates.^[14]^ However, the cytotoxic effect on FR‐positive MV‐4‐ 11, LoVo, and LoVo/Dx cell lines was promising but lower than the free drugs, MTX and epirubicin, suggesting the need for a cleavable linker between both drugs for the enhanced cytotoxic effect of the hybrid molecules.^[14]^ Cai et al. synthesized MTX‐diosgenin hybrid compounds with significant cytotoxic effects on MDA‐MB‐231 cell lines than the free MTX.^[15]^ The lengths of the linker influenced their cytotoxic effects and the hybrid with a disulfide linker was the most cytotoxic.^[15]^ Nadhum et al. prepared an MTX‐silibinin hybrid with higher anticancer activity against the HEP‐2 cell line (Larynx carcinoma) than MTX or silibinin.^[16]^ Semenenko et al. prepared MTX‐betulonic acid hybrids with different linkers. However, the hybrids’ capability to penetrate Caco‐2 cells was not significant when compared to methotrexate, resulting from the increased lipophilic nature of the hybrids.^[17]^

Hybrid compounds are useful for improving therapeutic effects, decreasing unwanted side effects, reducing the risk of developing drug resistance, promoting interaction with two or more targets, lowering the occurrence of drug‐drug adverse effects, and improving patient compliance. Due to the advantages of hybrid molecules, researchers have developed hybrid compounds for the treatment and management of numerous human diseases.[^18^, ^19^] The hybrid molecule can be transported to numerous targets involved in cancer cell proliferation and exhibit similar pharmacokinetic profiles, decreasing the occurrence of drug‐drug interactions.[^20^, ^21^] To overcome the challenges of the current drugs and enhance their therapeutic outcomes, BPs, MTX, and Vitamin D compounds were explored as precursors for the synthesis of hybrid compounds. Hybridizing BPs with known anticancer drugs has been reported to result in synergistic anticancer effects with the potential to enhance the treatment outcomes of the BPs.^[22]^ Vitamin D, which is beneficial for healthy bones, is ideal for hybridizing with other anticancer drugs for improved bone remodelling.^[23]^ The hybridization of MTX with other anticancer agents was found to be effective and a promising therapeutic agent in the treatment of cancer patients.^[24]^ Based on the reported therapeutic efficacy of hybrid‐based compounds, hybrid compounds containing BP, vitamin D, and methotrexate were synthesized and evaluated as potential anticancer agents against bone metastasis. The compounds were characterized using FTIR, ^1^H‐,^31^P, ^13^C‐NMR, UHPLC‐HRMS, and EDX, followed by *in vitro* hydroxyapatite bone binding assay and cytotoxicity, prediction of ADME and toxicity, molecular docking, and dynamics studies.

## Experimental

### Materials

All the reagents and solvents used in this work were of analytical grade and were purchased from Merck Chemicals, South Africa. They were used without further purification.

### Characterization

The melting points of the synthesized compounds were determined using a Stuart melting point apparatus model SMP 11, LabFreind, South Africa. The reactions were monitored by Thin Layer Chromatography. The synthesized compounds were purified by gravity column chromatography using silica gel adsorbent with a particle size range of 40–63 μm. FTIR was performed using a Perkin Elmer spectrum FTIR spectrometer in the range of 4000–400 cm^−1^ to study the functional groups present in the synthesized compounds. The mass spectra of the synthesized compounds hybrid compounds were recorded on a Bruker Compact Q‐TOF mass spectrometer Bruker Daltonics. The ^1^H, ^13^C, and ^31^P NMR spectra of the compounds were recorded on a Bruker ultrashield 500 NMR spectrometer. The NMR analysis of the synthesized hybrid compounds was performed using either deuterium oxide (D_2_O), deuterated chloroform (CDCl_3_), or deuterated dimethyl sulfoxide (DMSO‐*d_6_*).

### Synthesis

#### Synthesis of *Trans*‐[Pt(DACH)(Pamidronate)]

**Scheme 1:**
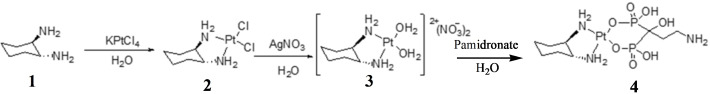
Synthesis of pamidronate‐[Pt(NO_3_)_2_(DACH)] (**4**).

Trans‐[Pt(DACH)(H_2_O)_2_](NO_3_)_2_ (**3**) complex was prepared by a known protocol by first synthesizing trans‐cyclohexane‐1,2‐diamine (DACH) (**1**), (trans‐cyclohexane‐1,2‐diamine)platinum(II) chloride trans‐[PtCl_2_(DACH)] (**2**)^[25]^ followed by reaction with silver nitrate^[26]^ (Scheme 1).

*Trans*‐[Pt(DACH)(Pamidronate)] (**4**) was prepared by a modified method^[27]^ in which **3** (100 mg, 0.216 mmol, 1.00 equiv) was dissolved in 10 mL of distilled water followed by the addition of pamidronate (60.2 mg, 0.216 mmol, 1.00 equiv). The reaction mixture was stirred at room temperature for 3 days, and the flask was covered with aluminum foil to protect the reaction from light. The reaction mixture was concentrated, and acetone was added, resulting in the formation of solids which were filtered and further washed with acetone and dried under vacuum to afford yellow product (**4**) (amount=118 mg; % yield=73; mp=220–221 °C, HRMS: m/z calcd. for [C_9_H_22_N_3_O_7_P_2_Pt+H]^+^: 541.1, found: 541.1). FTIR (ATR, cm^−1^): *v*=2941 and 2862 sp^3^(C−H), 1359 (C−N), 1044–982 (P=O), 910 (P−OH), 540 (Pt−N), and 470 (Pt−O) cm^−1^. ^1^H‐NMR (500 MHz, D_2_O, ppm): δ=3.81–3.76 (m, 1H), 3.45 (dd, 2H), 3.35 (dd, 2H), 3.26 (s, 1H), 2.96 (t, 2H), and 1.05 (d, *J*=5.0 Hz, 2H). ^31^P‐NMR (202 MHz, D_2_O, ppm): δ=19.21 (Pt), ^13^C‐NMR (125 MHz, D_2_O, ppm): δ=67.93 (C5 & C4), 66.60 (C11), 39.19 (C21), 32.94 (C6 & C3), 27.23 (C2 & C1), and 17.98 (C20). The numbering of the carbons is reported in Figure S1d.

#### Synthesis of Methotrexate‐Vitamin B1

**Scheme 2:**
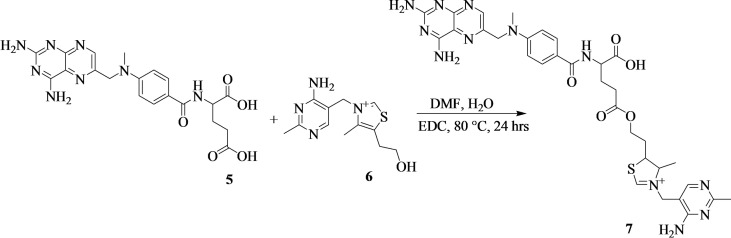
Synthesis of methotrexate‐vitamin B1 (**7**).

The synthesis of methotrexate‐vitamin B1 (**7**) was prepared by a modified method[^28^, ^29^] (Scheme 2). Methotrexate (**5**) (500 mg, 1.10 mmol, 1.00 equiv) was dissolved in dried dimethylformamide (8 mL) and vitamin B1 (**6**) (292 mg, 1.10 mmol, 1.00 equiv) was dissolved in distilled water (2 mL). The mixture was heated at 80 °C followed by the addition of 1‐ethyl‐3‐(3‐dimethylaminopropyl)carbodiimide (232 mg, 1.21 mmol, 1.10 equiv) and continuous of the reaction mixture for 24 hours. The compound was purified by column chromatography (MeOH:Hexane:H_2_O (3 : 2 : 1) to afford brown powder (amount=410 mg; % yield=47; mp=105–106 °C; Rf=0.29 (MeOH:Hexane:H_2_O (3 : 2 : 1); HRMS: m/z calcd. for [C_32_H_39_N_12_O_5_S+H]^+^: 703.2, found: 703.2. FTIR (ATR, cm^−1^): *v*=3346–3213 (N−H), 2830 (C−H), 1607 (C=O), 1514 (C=C/C=N), and 1210 (C−O) cm^−1^. ^1^H‐NMR (500 MHz, d_6_‐DMSO, ppm): δ=8.78 (s, 1H), 8.66 (s, 1H), 7.75 (d, *J*=5.0 Hz, 2H), 6.83 (d, *J*=5.0 Hz, 2H), 4.84 (s, 2H), 4.36 (s, 2H), 3.56 (t, 2H), 3.23 (s, 3H), 2.87 (t, 1H), 2.72 (s, 2H), 2.30 (s, 3H), and 0.98 (t, 3H). ^13^C‐NMR (125 MHz, d_6_‐DMSO, ppm): δ=174.36 (C27), 174.13 (C29), 166.84 (C20), 163.16 (C46), 158.88 (C44 & C1), 151.32 (C48), 150.62 (C3), 149.46 (C13), 148.97 (C5), 129.42 (C18 & C16), 128.94 (C4), 122.29 (C17), 121.75 (C19 & C15), 111.59 (C43) 61.83 (C40), 55.29 (C34), 54.97 (C23), 52.26 (C41), 42.46 (C11), 36.75 (C36), 34.54 (C14), 30.93 (C35), 29.82 (C25), 25.67 (C24), 16.03 (C49), and 15.15 (C42). The numbering of the carbons is reported in Figure S2c.

#### Synthesis of Methotrexate‐Propane‐1,3‐Diamine

**Scheme 3:**
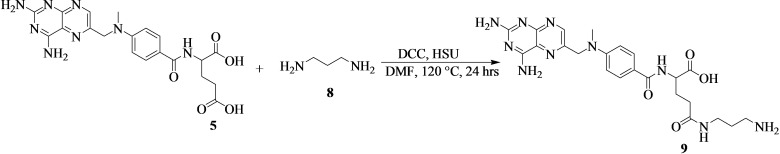
Synthesis of **methotrexate‐propane‐1,3‐diamine** (**9**).

Methotrexate‐propane‐1,3‐diamine (**9**) was prepared as shown in Scheme 3. A mixture of **5** (5000 mg, 11.0 mmol, 1.00 equiv) and propane‐1,3‐diamine (**8**) ((0.9 mL) in dried dimethylformamide (20 mL) was stirred at room temperature. *N,N*′‐dicyclohexylcarbodiimide (2496 mg, 12.1 mmol, 1.10 equiv), and *N*‐hydroxysuccinimide (1876 mg, 12.1 mmol, 1.10 equiv) were added to the mixture, and the solution was heated at 120 °C for 24 hours. The reaction mixture was monitored by thin‐layer chromatography using MeOH:Hexane (6 : 4). The reaction was cooled, followed by the purification by column chromatography (MeOH:Hexane (6 : 4) and concentration on a roti‐evaporator to afford yellow crystals (**9**) (4.2 g and % yield of 72 %).

#### Synthesis of Methotrexate‐Propane‐1,3‐Diamine‐Cinnamic Acid

**Scheme 4:**
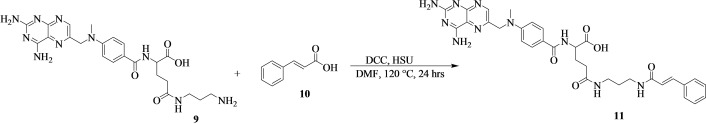
Synthesis of methotrexate‐propane‐1,3‐diamine‐cinnamic acid (**11**).

In a round bottom flask, **9** (500 mg, 0.979 mmol, 1.00 equiv) was dissolved in dried dimethylformamide (10 mL). Cinnamic acid (**10**) (145 mg, 0.979 mmol, 1.00 equiv) was added to the solution, followed by the addition of *N,N*′‐dicyclohexylcarbodiimide (222 mg, 1.08 mmol, 1.10 equiv), and *N*‐hydroxysuccinimide (167 mg, 1.08 mmol, 1.10 equiv). The reaction mixture was heated at 120 °C for 24 hours. The reaction was monitored by thin‐layer chromatography until the reaction was complete. The compound was purified by column chromatography (DCM:EtOAc (7 : 3) to afford a yellow powder product (**11**) (Scheme 4) (amount=439 mg; % yield=68; mp=100–103 °C; Rf=0.62 (DCM:EtOAc (7 : 3); HRMS: m/z calcd. for [C_32_H_36_N_10_O_5_+H]^+^: 641.3, found: 641.3). FTIR (ATR, cm^−1^): *v*=2933–2903 sp^3^(C−H), 1700 (C=O), 1623–1617 (HNC=O), 1521 cm^−1^ (Ar−C), 1224 (C−N), and 989 (C−H) cm^−1^. ^1^H‐NMR (500 MHz, CDCl_3_, ppm): δ=8.13 (s, 1H), 7.76 (d, *J*=15 Hz, 1H), 7.71 (s, 1H), 7.68 (s, 1H), 7.55 (d, *J*=10 Hz, 2H), 6.92 (d, *J*=10 Hz, 2H), 6.48 (d, *J*=15 Hz, 1H), 4.23 (s, 2H), 3.90 (s, 2H), 3.48 (t, 2H), 2.91 (s, 3H), 1.37 (q, 2H), and 1.16 (q, 2H). ^13^C‐NMR (125 MHz, CDCl_3_, ppm): δ=171.04 (C27), 165.07 (C38, C29, & C20), 146.52 (C1), 140.80 (C3), 134.98 (C13), 134.22 (C5), 130.55 (C9), 129.51 (C8), 128.91 (C42), 128.75 (C46, C44, C18 & C16), 128.27 (C45), 127.32 (C48, 43, C19 & C15), 121.12 (C17), 117.58 (C39) 48.44 (C11), 33.22 (C14), 31.02 (C36 & 34), 29.67 (C25), 25.56 (C35), and 24.84 (C24). The numbering of the carbons is reported in Figure S3c.

#### Synthesis of Methotrexate‐Propane‐1,3‐Diamine‐Oleanolic Acid

**Scheme 5:**
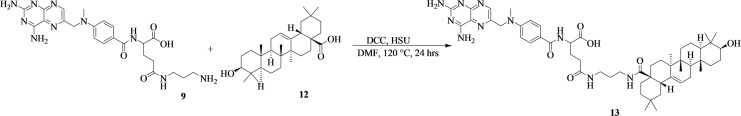
Synthesis of methotrexate‐propane‐1,3‐diamine‐oleanolic acid (**13**).

A mixture of **9** (500 mg, 0.979 mmol, 1.00 equiv) and oleanolic acid (**12**) (447 mg, 0.979 mmol, 1.00 equiv) was dissolved in 10 mL of dried dimethylformamide (Scheme 5). *N,N*′‐dicyclohexylcarbodiimide (222 mg, 1.08 mmol, 1.10 equiv), and *N*‐hydroxysuccinimide (167 mg, 1.08 mmol, 1.10 equiv) were added to the mixture. The reaction mixture was heated at 120 °C for 24 hours. The reaction mixture was purified by column chromatography (Hexane:EtOAc 7 : 3) to obtain a white powder (**13**) (amount=689 mg; % yield=72; mp=210–215 °C; Rf=0.63 (Hexane:EtOAc 7 : 3); HRMS: m/z calcd. for [C_53_H_76_N_10_O_6_+H]^+^: 948.6, found: 948.7. FTIR (ATR, cm^−1^) *v*=3586 (O−H), 3339 (RO=C−NHR), 2930 and 2899 sp^3^(C−H), 1466 (Ar−C/C=C), 1629–1694 (C=O), 1214 (C−N), and 1057 (C−H) cm^−1^. ^1^H‐NMR (500 MHz, CDCl_3_, ppm): δ=8.53 (s, 1H), 7.55 (d, *J*=10 Hz, 1H), 7.37 (s, 1H), 7.14 (s, *J*=10 Hz, 1H), 5.59 (s, 1H), 5.37 (s, 2H), 5.29 (s, 4H), 4.23 (t, 2H), and 1.62–0.78 (m, 20H). ^13^C‐NMR (125 MHz, CDCl_3_, ppm): δ=182.68 (C27), 172.74 (C38, C29, & C20), 169.31 (C1), 157.11 (C3), 143.66 (C13), 142.55 (C5), 139.24 (C9), 123.28 (C8), 122.56 (C18 & C16), 114.03 (C19 & C15), 79.02 (C64), 55.25 (C23), 47.66 (C59), 46.49 (C11), 41.64 (C43), 39.31 (C41), 38.76 (C65), 37.09 (C36 & 34), 33.76 (C56), 30.66 (C61), 29.67 (C63), 28.10 (C68), 27.20 (C42 & C40), 25.91 (C48), 25.57 (C50 & C51), 24.84 (C67), 23.48 (C58), 17.15 (C60), 15.53 (C70 & C71), and 15.30 (C69). The numbering of the carbons is reported in Figure S4c.

#### Synthesis Of Methotrexate‐Propane‐1,3‐Diamine‐Artesunate

**Scheme 6:**
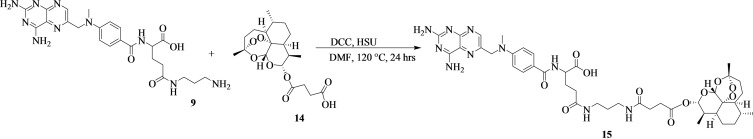
Synthesis of methotrexate‐propane‐1,3‐diamine‐artesunate (**15**).

The reaction scheme showing the synthesis of methotrexate‐propane‐1,3‐diamine‐artesunate (**15**) is Scheme 6. 500 mg of **9** (0.979 mmol, 1.00 equiv) was dissolved in 10 mL of dried dimethylformamide. Artesunate (**14**) (376 mg, 0.979 mmol, 1.00 equiv) was added in a reaction followed by *N,N*′‐dicyclohexylcarbodiimide (222 mg, 1.08 mmol, 1.10 equiv), and *N*‐hydroxysuccinimide (167 mg, 1.08 mmol, 1.10 equiv). The reaction was heated at 120 °C, for 24 hours and monitored by thin‐layer chromatography. The reaction mixture was purified by column chromatography (Hexane:MeOH:EtOAc (6 : 2 : 2)) to obtain a brown viscous oil (amount=420 mg; % yield=48; Rf=0.13 (H:6; MeOH:2; EtOAc:2); HRMS: m/z calcd. for [C_42_H_56_N_10_O_11_+H]^+^: 876.4, found: 876.4). FTIR (ATR, cm^−1^) *v*=2904–2936 sp^3^(C−H), 1639 (HNC=O), 1717 (C=O), 1543 (C=C/C=N), 1218 (C−N), and 1025 (C−H) cm^−1^. ^1^H‐NMR (500 MHz, CDCl_3_, ppm): δ=8.11 (s, 1H), 6.49 (d, *J*=10 Hz, 1H), 6.01 (s, 1H), 5.71 (s, 1H), 4.25 (s, 2H), 3.41 (s, 2H), 2.71 (s, 3H), and 1.92–1.12 (m, 8H). ^13^C‐NMR (125 MHz, CDCl_3_, ppm): δ=175.06 (C27), 172.14 (C66) 171.86 (C61, C29, & C20), 164.09 (C1), 160.57 (C3), 157.96 (C13), 157.46 (C5), 109.20 (C39), 99.64 (C50), 82.41 (C41), 49.42 (C23), 47.16 (C11), 44.64 (C45), 42.42 (C14), 36.16 (C36 & 34), 34.05 (C38), 33.75 (C43), 32.97 (C47), 32.75 (C51), 25.66 (C62), 25.41 (C25), 25.00 (C48), 24.86 (C24), 24.69 (C53), 23.92 (C55), and 12.57 (C57). The numbering of the carbons is reported in Figure S5c.

#### Synthesis of Vitamin D2‐4‐Ferrocene‐Ketobutanoic Acid

**Scheme 7:**
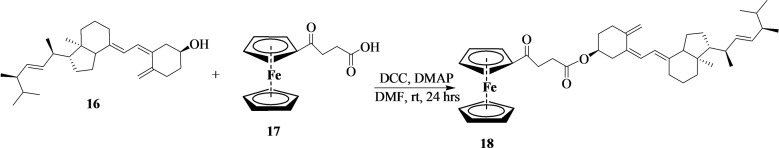
Synthesis of vitamin D2‐4‐ferrocene‐ketobutanoic acid (**18**).

4‐Ferrocene ketobutanoic acid was synthesized according to our previous reports.[^4^, ^30^, ^31^] Vitamin D2 (**16**) (300 mg, 0.76 mmol, 1.00 equiv) was dissolved in 10 mL of dried dimethylformamide. The solution was stirred at 0 °C for 30 minutes, and *N,N*′‐dicyclohexylcarbodiimide (171 mg, 0.83 mmol, 1.10 equiv) was added. 4‐Ferrocene ketobutanoic acid (**17**) (239 mg, 0.76 mmol, 1.00 equiv) was also added to the reaction mixture, followed by the addition of 4‐dimethylaminopyridine (92.4 mg, 0.76 mmol, 1.00 equiv). The reaction was stirred at room temperature for 24 hours and concentrated on a rotatory evaporator. The compound was then purified by column chromatography (DCM: EtOAc (7 : 3)) to obtain an orange powder (**18**) (Scheme 7) (amount=346 mg; % yield=64; mp=140–142 °C; Rf=0.80 (DCM:7; AtOAc:3); HRMS: m/z calcd. for [C_44_H_62_FeO_3_+H]^+^: 694.4, found: 695.5). FTIR (ATR, cm^−1^) *v*=2931 and 2910 sp^3^(C−H), 1625–1626 (C=O), 1577 (C=C), 1195–1244 (C−O), and 645 (C−Fe) cm^−1^. ^1^H‐NMR (500 MHz, CDCl_3_, ppm): δ=7.58 (s, 1H), 4.83 (s, 2H), 4.55 (s, 2H), 4.30 (s, 5H, C_5_H_5_), 4.18 (s, 2H), 3.26 (t, 2H), 2.72 (t, 2H), and 2.00–1.11 (m, 20H). ^13^C‐NMR (125 MHz, CDCl_3_, ppm): δ=204.26 (C14), 170.98 (17), 156.79 (C28 & C25), 154.43 (C35 & C34), 72.41, (C11 & C5), 70.06 (C13, C10, C9, C4, C3, C2, C1), 69.26 (C20), 54.89 (C32), 49.99 (C36), 49.13 (C42), 35,20 (C24), 33.95 (C33), 32.45 (C44), 30.69 (C38), 29.67 (C46), 28.50 (C22), 26.14 (C16), 25.63 (C30), 25.42 (C45), 24.93 (C43), 24.68 (C40 & C39), 22.66 (C41 & C37), and 14.07 (C6 & C7). The numbering of the carbons is reported in Figure S6c.

#### Synthesis of Vitamin D2‐Artesunate

**Scheme 8:**
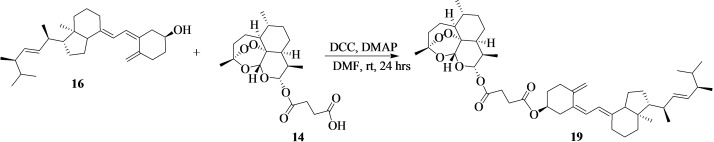
Synthesis of vitamin D2‐artesunate (**19**).

The reaction of vitamin D2‐artesunate (**19**) is presented in Scheme 8. In a round bottom flask, **16** (300 mg, 0.76 mmol, 1.00 equiv) was dissolved in dried dimethylformamide (10 mL). The reaction was placed in an ice bath for 30 minutes and *N,N*′‐dicyclohexylcarbodiimide (171 mg, 0.83 mmol, 1.10 equiv) was added. **14** (290 mg, 0.76 mmol, 1.00 equiv) was also added, followed by the addition of 4‐dimethylaminopyridine (92.4 mg, 0.76 mmol, 1.00 equiv). The reaction mixture was stirred at room temperature overnight. The mixture was concentrated on a rotary evaporator, and the reaction mixture was purified by column chromatography (MeOH:Hexane 6 : 4) to obtain a brown viscous compound. Amount=383 mg; % yield=65; Rf=0.85 (MeOH:6; H:4); HRMS: m/z calcd. for [C_47_H_70_O_8_+H]^+^: 762.5, found: 762.5. FTIR (ATR, cm^−1^) *v*=2905–2932 sp^3^(C−H), 1714 (C=O), 1628–1578 (C=C), 3342 (O−H), and 1020 (C−O) cm^−1^. ^1^H‐NMR (500 MHz, CDCl_3_, ppm): δ=7.14 (d, *J*=10 Hz, 1H), 6.20 (s, 1H), 5.78 (d, *J*=10 Hz, 1H), 5.71 (s, 1H), 5.44 (s, 2H), 3.23–3.18 (m, 1H), 2.90 (s, 2H) 2.14 (s, 2H), and 1.92–0.86 (m, 20H). ^13^C‐NMR (125 MHz, CDCl_3_, ppm): δ=171.82 (C55 & C51), 157.08 (C28), 153.93 (C9), 147.09 (C6), 135.00 (C16), 124.45 (C15), 123.93 (C7), 119.09 (C8), 109.19 (C29), 104.47 (C31), 99.64 (C42), 91.56 (C33), 82.41 (34), 80.09 (C1), 56.52 (C10), 49.19 (C13), 45.24 (C17), 44.64 (C23), 42.44 (C5), 37.29 (C14), 35.35 (C37), 33.86 (C25), 33.47 (C30), 32.75 (C19), 31.41 (C39), 29.66 (C27), 26.19 (C4), 25.60 (C43), 24.89 (C54 & C53), 23.94 (C11), 23.52(C12), 22.02 (C26), 20.17 (C21 & C20), 18.54 (C45), 12.59 (C22), 11.98 (C18), and 11.01 (C49). The numbering of the carbons is reported in Figure S7c.

#### Synthesis of Vitamin D2‐Cinnamic Acid

**Scheme 9:**
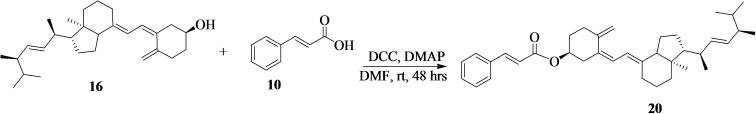
Synthesis of vitamin D2‐cinnamic acid (**20**).

Synthesis of vitamin D2‐cinnamic acid (**20**) is shown in Scheme 9. **16** (300 mg, 0.76 mmol, 1.00 equiv) was dissolved in dried dimethylformamide (10 mL). *N,N*′‐dicyclohexylcarbodiimide (171 mg, 0.83 mmol, 1.10 equiv) was added followed by the addition of **10** (112 mg, 0.76 mmol, 1.00 equiv), and 4‐dimethylaminopyridine (92.3 mg, 0.76 mmol, 1.00 equiv) to the reaction mixture in an ice bath for 15 minutes. The mixture was then stirred at room temperature for 48 hours. The reaction mixture was concentrated and purified by column chromatography using (DCM:EtOAc (7 : 3)) eluent to afford a cream‐white powder (amount=338 mg; % yield=82; mp=145–146 °C; Rf=0.71 (DCM:EtOAc 7 : 3); HRMS: m/z calcd. for [C_37_H_50_O_2_+H]^+^: 526.4, found: 527.4. FTIR (ATR, cm^−1^) *v*=2910–2930 sp^3^(C−H), 1707 (C=O), 1600–1645 (C=C), and 1233 (C−O) cm^−1^. ^1^H‐NMR (500 MHz, CDCl_3_, ppm): δ=7.67 (d, *J*=15 Hz, 2H), 7.48 (s, 2H), 6.78 (s, 1H), 6.74 (s, 1H), and 2.05–0.90 (m, 16H). ^13^C‐NMR (125 MHz, CDCl_3_, ppm): δ=166.76 (C30), 154.04 (C9), 143.38 (C28), 139,23 (C6), 134.76 (C16), 130.01 (C33), 128.88 (C37 & C35), 127.90 (C36), 119.52 (C29), 114.03 (C31), 56.23 (C10), 49.87 (C13), 32.79 (C19), 31.91 (C3), 30.97 (C27), 29.67(C11), 26.30 (C12), 25.48 (C26), 25.38 (C22), 24.67(C24), 22.66 (C21 & C20), and 14.04 (C18). The numbering of the carbons is reported in Figure S8c.

### Molecular Docking Study

Eight of the synthesized compounds were selected, optimized, refined, and saved in a Molecular Operating Environment (MOE) using molecular operation environment software. The studied receptors (Human Tyrosine Phosphatase (PDB ID: 1wch)^[32]^ and human 3‐alpha hydroxysteroid dehydrogenase type 3 (PDB ID: 4xo6)^[33]^ were retrieved from the protein data bank. The receptors were prepared for docking and saved in MOE format. The studied compounds were optimized and saved in MOE format for further analysis using induced fit for refinement and triangle matcher for placement.

### Molecular Dynamics Calculations

This study involves the use of GROMACS software for the compounds with the highest binding affinity as well as the referenced compounds. Compounds **19** and **15**, with the topmost potential to inhibit Human Tyrosine Phosphatase (PDB ID: 1wch) and human 3‐alpha hydroxysteroid dehydrogenase type 3 (PDB ID: 4xo6), were subjected to a molecular dynamic simulation study. This study was accomplished using Charmm36m as a force field which was embedded in GROMACS software^[34],^ and the simulated compounds were solvated using proper water molecules. Counter‐ions were added to the system to obtain ionic concentrations in the simulation system. The molecular dynamic simulation study was done in the NPT ensemble with constant pressure and temperature. The studied molecular dynamic simulation was carried out using 100000 ps.^[35]^

### Swissadme Prediction Tool

SwissADME is a reliable and open‐access online software that was used to predict some physicochemical properties of the compounds (http://www.swissadme.ch/).

### *In Silico* Toxicity Predictions

In Silico Toxicity predictions of the compounds were studied using free online software, ProTox‐II (http://tox.charite.de/protox_II/) and GUSAR (http://www.pharmaexpert.ru/GUSAR/antitargets.html) for quantitative prediction of LD50 values, the potential routes of administration (i. e., oral, intraperitoneal, intravenous, and subcutaneous) and the environmental toxicity of the synthesized compounds. *In silico* cardiac toxicity of the compounds was also predicted using the free online web server, http://predherg.labmol.com.br/.

### *In Vitro* Cytotoxicity Evaluation on MCF‐7and HT‐29 Cell Lines

Cell viability was determined using tetrazolium salt (3‐(4, 5‐dimethylthiazol‐2‐yl)‐2,5‐ diphenyltetrazolium bromide) (MTT) assay. Briefly, MCF‐7 cells were seeded in 35 mm cell culture plates at a density of 3×10^5^ cells and incubated for 4 h prior to experimentation. Incubation was performed for 20 h after treatment, in which the medium was replaced with a fresh medium containing tetrazolium salt. The presence of the mitochondrial enzyme succinate dehydrogenase cleaved the tetrazolium salt to give a blue product (formazan). The optical density was read at 590 nm wavelength using Perkin‐Elmer, VICTOR3TM plate reader.

The colon cell line was grown in an incubator until 80 % of cell confluence was reached. Trypan blue was used to check the viability of the cells. The desired concentration of cells used was 1.0×10^5^ for the cell lines, and a total of 100 μL of cells/mL was seeded in the 96 well plates for 24 h. After 24 h of incubation, the cell line was treated with different concentrations of the compound, 100 μg/mL, 50 μg/mL, 25 μg/mL, 12.5 μg/mL, and 6.25 μg/mL. The positive control used was cisplatin at the concentration of 200 μg/mL. The negative control was DMSO with cells (0.1 %) and cells with media only. After 24 h of treatment, 10 μL of AlamarBlue reagent was added in each well in the dark, and the plates were wrapped with aluminium foil as the dye is sensitive to light. It was incubated for 2 h, after which the plates were read by a fluorescence microplate reader (Synergy HT).

## 2 Results

### 2.1 FTIR, ^1^H‐, ^31^P‐ and ^13^C‐NMR, Spectrum OF *Trans*‐[Pt(DACH)(Pamidronate)] (4)

The complex **4** is synthesized for the first time in this study. Table 1 depicts the physical properties of the complex.

**Table 1:** Physical properties and structure of the complexes.

Compound	Mass(g)	%Yield	Solubility	Colour	Melting Point (°C)	Structure
[Pt(DACH)(Pamidronate)]	0.118	73	H_2_O	yellow	220–221	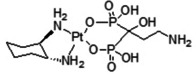

The FTIR spectrum of **4** (Figure S1a) exhibited a characteristic band pattern, confirming the appearance of the corresponding bisphosphonate ligands in the synthesized platinum complex. The important peaks observed at 2941 and 2862 cm^−1^ are attributed to C−H stretching. The prominent peak at 1359 cm^−1^ region is attributed to the bending modes of C−N of the amine. The peaks in the range of 1044–982 cm^−1^ result from the P=O stretch and the peak at 910 cm^−1^ is due to the P−OH stretch. Similar findings for bisphosphonates were observed by Alvarez‐Valdes et al., Dlamini et al., and Qiu et al.[^27^, ^36^, ^37^] The C−O stretch was visible at 1153 cm^−1^. The appearance of the bands at 540 and 470 cm^−1^ region is ascribed to Pt−N and Pt−O stretching, confirming the bisphosphonate ligand is coordinated to the platinum complex. Similar findings were reported by Ray et al. for platinum‐based complexes.^[38]^ In the ^1^H‐NMR spectrum of **4**, the signals of amino groups were visible at 3.80–3.77 ppm, the multiplet signals due to the aliphatic protons of the phosphonate ligand range between 3.46–3.44 ppm, the signals corresponding to amine and hydroxyl group were at 3.36–3.26 ppm, the protons of diaminocyclohexane ring were visible between 2.96–1.04 ppm (Figure S1b). The coordination of bisphosphonate ligand to platinum *via* the oxygen atoms of amino bisphosphonate was further confirmed by ^31^PNMR spectroscopy. The ^31^PNMR spectrum displayed a characteristic peak at 19.21 ppm (Figure S1c). These chemical shifts confirm the formation of the complexes. ^13^C‐NMR spectra confirmed the number of carbons in the complex. Carbon signals due to the phosphonates were displayed in a range of 67.93–66.60 ppm, and the carbons due to diaminocyclohexane were visible at 39.19–17.98 ppm (Figure S1d). The UHPLC‐HRMS spectrum of complex **4** exhibited a peak at 541.1 (Figure S1e), which further confirmed the successful synthesis of the compound.

### 2.2 Spectroscopic Analysis of the Hybrid Compounds Containing Methotrexate

The FTIR spectrum of **7** revealed significant peaks for C−H stretch at 2981 cm^−1^, H−C= stretch at 3191 cm^−1^, C=C stretch at 1551–1400 cm^−1^, N−H stretch at 3314 cm^−1^, C=O stretch at 1611 cm^−1^, O−H stretch at 3500–2500 cm^−1^, C−O stretch at 1304–1044 cm^−1^, and C−S stretch of the thiazole group at 774 cm^−1^, confirming the successful synthesis of the compound (Figure S2a). El‐Megharbel reported a similar finding for C−S stretching at 773 cm^−1^ for the thiazole group in a vitamin‐B1 complex.^[39]^ The ^1^H‐NMR spectrum showed a signal at 4.84 ppm ascribed to NH_2_, the signals at the range of 3.23–0.96 ppm are due to three CH_3_ (one from MTX and two from Vitamin B1), the signal at 8.66 ppm is attributed to amide N−H and the signals at 7.75–6.82 ppm are ascribed to aromatic protons (Figure S2b). The ^13^CNMR spectrum revealed the ester O−C=O peak at 174.13 ppm, a crucial functional group on the linker of the hybrid molecule (Figure S2c).

The FTIR spectrum of **11** displayed the following absorption peaks: C−H stretch at 2924 and 2853 cm^−1^, H−C= stretch at 3068 cm^−1^, N−H stretch at 3274 cm^−1^, C=C stretch at 1600–1400 cm^−1^, C=N stretch at 1329 cm^−1^, C=O stretch at 1618 cm^−1^, and C−O stretch at 1218–1082 cm^−1^ (Figure S3a). Similar bands were visible on MTX derivatives reported by Nemat et al.^[40]^

The ^1^H‐NMR spectra of **11** showed the appearance of the singlet signal at 8.13 ppm (s, 1H) which is ascribed to the C9 pyrazine proton. The presence of the doublet signals at 7.77–7.74 ppm is attributed to the aromatic protons (Figure S3b). The ^13^CNMR spectrum displayed signals of the carboxylic and amide carbons at 171.04 ppm and 165.07 ppm, respectively (Figure S3c).

The FTIR spectrum of **13** showed characteristic bands for C−H stretch at 2931 and 2832 cm^−1^, N−H stretch at 3597 and 3288 cm^−1^, C−O stretch 1205–1056 cm^−1^, C=N stretch at 1377 cm^−1^, C=C stretch at 1611–1427 cm^−1^, and C=O stretch at 1760 and 1674 cm^−1^ (Figure S4a). ^1^H‐NMR spectrum of **13** showed signals between 0.79–1.27 ppm which are attributed to the CH_3_ groups. The presence of a singlet signal at 8.53 ppm (s, 1H) is ascribed to the pyrazine proton, and the doublet signals at 7.56–7.54 ppm and 7.15–7.13 ppm are attributed to the aromatic protons (Figure S4b). The ^13^C‐NMR spectrum of **13** showed a carboxylic signal at 182.68 ppm and the amide carbon signals at 172.74 ppm, 169.31 ppm, and 157.11 ppm. The signals assigned to the (C=N and C=C) were found at 143.66–139.24 ppm, and the signals of the aromatic carbons were visible between 123.28 and 114.03 ppm (Figure S4c). Similar signals were reported by Tang et al. for oleanolic acid‐based hybrid compounds.^[41]^

On the FTIR spectrum of **15**, important peaks confirming the successful synthesis of the hybrid molecules were significant for C−H stretch at 2931 and 2858 cm^−1^, N−H stretch, C=C stretch at 1637–1452 cm^−1^, C=N stretch at 1377 cm^−1^, C=O stretch at 1698 cm^−1^, C−O stretch at 1230–1008 cm^−1^, O−H stretch at 3314 cm^−1^, and −COO stretch at 872 cm^−1^ (Figure S5a). Lawal et al reported COO stretching for the endoperoxide bridge of artesunate at 890–820 cm^−1^.^[42]^ The ^1^H‐NMR spectrum of **15** showed the presence of a singlet signal at 8.11 ppm ascribed to the pyrazine proton (Figure S5b). The ^13^C‐NMR spectrum of **15** exhibited a carboxylic signal at 175.06 ppm, a ketone carbon signal at 172.14 ppm, and amide carbon at 171.86, 164.09, and 160.57 ppm. The CH_3_ and CH_2_ signals were significant at 1.12–1.17 ppm (Figure S5c). Similar findings were reported by Munnik et al. for ferrocenyl‐artesunate hybrid compounds.^[43]^ The UHPLC‐HRMS spectra of the hybrid compounds showed peaks at 703.2, 641.3, 948.7, 876.4, respectively, for **7**, **11**, **13**, and **15**, which further confirmed the successful synthesis of the compounds (Figures S2d, S3d, S4d, and S5d).

### 2.3 Spectroscopic Analysis of the Hybrid Compounds Containing Vitamin D2

The FTIR spectra of hybrid compounds containing Vitamin D2 are reported in the Supplementary Figures. The FTIR spectrum of **18** displayed peaks for C=O stretch at 1748 and 1674 cm^−1^, C−O ester stretch 1266–1056 cm^−1^, C−H stretch at 2931 and 2844 cm^−1^, C=C stretch at 1644–1440 cm^−1^, and C−Fe stretch at 650–527 cm^−1^ (Figure S6a). On the FTIR spectrum of **19**, the absorption peaks of O−H, −C−H, C=C, C=O, C−O ester, and −COO stretching were visible at 3327, 2931–2832, 1597–1476, 1710–1649, 1230–1020, and 872 cm^−1^, respectively (Figure S7a). The FTIR spectrum of **20** displayed characteristic peaks of O−H, −C−H, =C−H, C=C, C=O, and C−O ester stretching at 3264, 2931–2844, 3042, 1599–1464, 1698, 1266–1155 cm^−1^, respectively (Figure S8a).

In the ^1^H‐NMR spectrum of **18**, the singlet signals at 4.30 ppm and 4.18 ppm are attributed to the cyclopentadiene ring (Figure S6b). The ^1^H‐NMR spectrum of **19** showed the signals of CH_3_ at 0.86–1.54 ppm (Figure S7b). The ^1^H‐NMR spectrum of **20** revealed characteristic signals at 7.69–7.66 ppm for aromatic protons, multiplets between 2.05 and 1.62 ppm are attributed to the aliphatic protons and the signals at 1.28 ppm, and 0.90 ppm is assigned to the methyl protons of the compound (Figure S8b). Similar findings were reported by Pietraszek et al. for 1,25‐dihydroxyergocalciferol analogues.^[44]^ The ^13^C NMR spectrum of **18** showed signals for ketone carbon at 204.26 ppm, C=O ester carbon was found at 170.98 ppm, and the carbons due to the (C=C) were found at 156.79–154.43 ppm. The signal between 72.41 and 69.26 ppm was assigned to the cyclopentadiene carbon (Figure S6c). The ^13^C NMR spectrum of **19** displayed ester carbon, C=O at 171.82 ppm, and C=C at 157.08–109.19 ppm (Figure S7c). The ^13^C‐NMR spectrum of **20** showed a carboxylic C=O peak at 174.24 ppm, a signal of an ester carbon C=O at 170.38 ppm and an amide carbon at 162.86 ppm (Figure S8c). The ^13^C NMR spectra of the hybrid compounds containing vitamin D2 revealed characteristic signals of C=O stretch, resulting from the ester linker at 170.98, 171.82, and 166.76 ppm for **18**, **19**, and **20**, respectively. The UHPLC‐HRMS spectra of the hybrid compounds showed peaks at 695.5, 762.5, and 527.4, which also confirmed the successful synthesis of the hybrid compounds, **18**, **19**, and **20**, respectively (Figures S6d, S7d, and S8d).

### 2.4 *In Vitro* Hydroxyapatite Binding Assay of *Trans*‐[ Pt(DACH)(Pamidronate)] (4)

**Figure 1:**
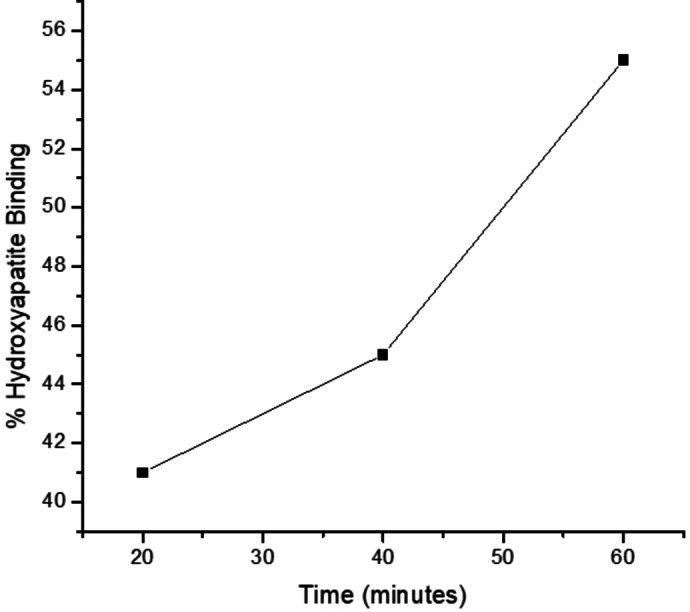
The HA binding assay affinity of the complex **4**.

Hydroxyapatite (HA) is the main component of bone mineral. The HA binding assay was conducted to evaluate the affinity of the complex for bone (Figure 1). The HA binding assay was conducted at 20 min, 40 min, and 60 min time intervals by known procedure.[^45^, ^46^] Within 20 min of incubation, 41 % of **4** was bound to HA, and as the time increased to 40 min, the percentage increased to 45 %. After 60 min, the amount bound to HA increased to 55 %. The results indicate that the complex has a moderate affinity for HA which increases with an increase in the reaction time.[^45^, ^46^] In an earlier study, the binding affinity of pamidronate was reported to be 60 %, which was the highest when compared to risedronate.^[47]^ Bisphosphonates with P−C‐P backbone structure have a high affinity for bone. In this study, the modification of the hydroxyl groups on bisphosphonates did not significantly reduce the binding affinity of the compound. The compound displayed promising bone binding affinity, revealing that preparing bisphosphonate/platinum‐based complexes is a promising approach to developing therapeutics that target the bones.

### 2.5 Swissadme Prediction of the Complex and Hybrid Compounds

The complexes were analyzed for their Absorption, distribution, metabolism, and excretion (ADME) properties using SwissADME software. The physicochemical properties, lipophilicity, water solubility, pharmacokinetics, and drug‐likeness of the complexes were also predicted using SwissADME, as shown in (Tables S1 and S2). The molar refractivity of **4, 7, 11, 13, 15, 18, 19**, and **20**, was 82.24, 194.55, 176.28, 269.82, 225.63, 190.87, 218.08, 168.72, respectively, and showed topological polar surface area (TPSA) of 210.97, 279.65, 231.44, 251.67, 294.66, 43.37, 89.52, and 23.60 Å^2^, respectively (Table 3 and Supplementary Table). The hybrid compounds containing Vitamin D2 pharmacophore displayed TPSA values of less than 140 Å^2^, revealing good absorption potential in the intestines. TPSA values are used to predict drug absorption, such as absorption in the intestines, drug bioavailability, and blood‐brain barrier penetration.^[48]^

The TPSA values of most of the hybrid compounds were in the range of 210.97–294.66 Å^2^, which is attributed to their high molecular weight (Tables S1 and S2). The lipophilicity of the compounds was also predicted using SwissADME, a vital parameter for drug development to predict their solubility, capability to penetrate *via* cell barriers, and uptake to the molecular target.^[49]^ The Log *P*_o/w_ (XLOGP3), (WLOGP), (MLOGP), (SILICOS‐IT), and Consensus Log *P*_o/w_ values of the complex and hybrid compounds were evaluated. The compounds’ Log P lipophilicity values were less than three except in the case of **13** and the hybrid compounds containing Vitamin D2 pharmacophore. The software was unable to predict the ADME properties of Vitamin D2 hybrid compounds. A lipophilicity value of greater than 5 contributes to features such as strong drug plasma protein binding, drug accumulation in the tissues, rapid drug metabolic turnover, and poor water solubility.^[50]^ The hybrid compounds were mostly water soluble except **13**. Good water solubility of drugs is important in drug pharmacokinetics and pharmacodynamics.^[51]^ Furthermore, the pharmacokinetic properties of the platinum complex and the hybrid compounds containing methotrexate were studied, and the BOILED‐Egg models are shown in Figures S9–S16. Cytochrome P450 isoforms, CYP1 A2, CYP2 C19, CYP2 C9, CYP2D6, and CYP3 A4 play a crucial role in drug bioactivation and safety. CYP3 A4 is one major important drug‐metabolizing enzyme found in the gastrointestinal tract and liver of the human body. It metabolizes most clinically approved drugs. The inhibition of CYP3 A4 can result in drug‐drug interactions, drug toxicity, and side effects. However, in some cases, its inactivation can enhance the drug therapeutic effectiveness of rapidly metabolized drugs by increasing their plasma levels.^[52]^ The drugs, **7**, **11**, and **13** were predicted to be inhibitors of CYP3 A4. The compounds displayed a low degree of GI absorption and were not permeable *via* the BBB. The compounds **4, 7, 11, 13**, and **15** were predicted to be P‐gp substrates. P‐glycoprotein (P‐gp) influences the uptake and efflux of drugs. It is expressed in tissues, such as the liver, small intestine, kidney, and the blood‐tissue barriers.^[53]^ Substrates of P‐glycoprotein can act as inhibitors or inducers of its function. The inhibition of P‐gp results in increased drug bioavailability.^[53]^ P‐glycoprotein is believed to be important against potentially toxic substances. It promotes drug elimination into the bile and urine and protects tissues such as the brain, placenta, testis, etc.[^53^, ^54^] The bioavailability score of the compound was either 0.11 or 0.17. A bioavailability score of 0.17 suggests a 17 % chance of having greater than 10 % bioavailability in rats.[^55^, ^56^] The drug‐likeness was studied using Lipinski, Ghose, Veber, Egan, and Muegge rules. However, the high molecular weight and TPSA values influenced their violation of drug‐likeness prediction. Based on Lipinski's assumptions, drug molecules that violate these rules are likely not to possess desirable features. It is important to indicate that many approved and existing drugs do not follow the drug‐likeness rules but are orally bioavailable.^[57]^

The predicted targets of compounds **4**, **7**, **11**, **13**, **15**, **18**, **19**, and **C20** are cytochrome P450, lyase, oxidoreductase, protease, kinase, transferase, family AG protein‐coupled receptor, and phosphatase (Figures S17–S24). These are promising drug targets explored in cancer therapies. Serine‐threonine protein phosphatase 2 A regulates some signaling pathways and its modulation has been reported to result in potent antitumor activity, indicating that it is a therapeutic target for treating cancer.^[58]^ Cytochrome enzymes are involved in multi‐organ metastasis and cancer progression. They are overexpressed in the tumour microenvironment. Targeting Cytochrome enzymes is beneficial in cancer therapy.^[59]^ Transferase enzymes, such as Carnitine palmitoyl transferase I, are involved in breast cancer survival and invasion. Carnitine palmitoyl transferase I have been identified as a tumour target for anticancer therapeutics.^[60]^ G protein‐coupled receptors are potential targets for the treatment of colorectal cancer, and they control different cellular functions, such as tumorigenesis, etc.^[61]^ Oxidoreductase enzymes such as NAD(P)H: quinone oxidoreductase 1 are overexpressed in different types of tumours and are a target for cancer therapy.^[62]^ Proteases promote tumour growth and progression, which is dependent on the supply of nutrients and oxygen. High levels of proteases in tumours at an early stage have been reported. They promote proliferation, tumour invasion, inflammatory cell recruitment, angiogenesis, metastasis, etc.^[63]^

### 2.6 Toxicity Prediction of the Complex and Hybrid Compounds

The ProTox‐II web server was used in the study for the prediction of oral toxicity, organ toxicity (hepatotoxicity), and toxicological endpoints (carcinogenicity, immunotoxicity, mutagenicity, and cytotoxicity). The organ toxicity (hepatotoxicity, mutagenicity, and cytotoxicity) of the compounds were all predicted as inactive, revealing that they are not capable of causing damage to the liver, genetic mutation, or cell death, respectively. However, **15, 18, 19**, and **20** were predicted to be immunotoxic. The predicted toxicity of the compounds is shown in Table S3. GUSAR‐free online software was used for quantitative in silico toxicity prediction of LD50 values, the four possible routes of administration (i. e., oral, intraperitoneal, intravenous, and subcutaneous), and environmental toxicity of the synthesized compounds (Table S4). The hybrid compounds were mostly predicted to belong to classes 4 and 5 and are suitable for intraperitoneal administration, suggesting the non‐toxic nature of the drugs. In most cases, the compounds were within the applicability domain of models.

The bioaccumulation factor (*BCF*) is the ratio of the chemical concentration in an aquatic organism to that in water at a condition of a steady state.^[64]^ The *BCF* values of the hybrid compounds ranged from −0.042–0.945 when compared to the precursors in the range of −0.620–0.684 (Table S5). The *Daphnia magna* LC_50_ represents the concentration in water that can kill half of a population of *Daphnia magna* (a water flea). The range LC_50_ values of the hybrid compounds were in the range of 5.239–7.472, and the precursors were in the range of 3.222–6.279. The fathead minnow LC_50_ is the concentration in water that can kill half of a population of fathead minnows (*Pimephales promelas*). The fathead minnow values of all compounds varied from −1.776–−8.891. *Tetrahymena pyriformis* IGC_50_ is a 50 % growth inhibitory concentration of *T. pyriformis* organism. The compounds’ IGC_50_ values ranged from 0.449–3.142. Most of the compound falls in the applicability domain of models.

The antitarget, hERG K+ channels are important and should be considered in drug development. The blockage of the hERG K+ channels results in cardiotoxicity, such as lethal cardiac arrhythmia.^[65]^ Some drugs have been withdrawn from clinical use due to severe side effects.^[66]^ The human ether‐a‐go‐go related gene (hERG) channel is expressed in the nervous system and the heart. It is crucial to predict a drug candidate that can block the hERG potassium ion channel at the early stage of drug discovery.^[66]^ The hybrid compounds were evaluated to predict their capability to block hERG K+ channels (Table S6). Four outcomes were obtained namely: binary prediction Consensus applicability domain model), multiclass prediction (Consensus applicability domain model), probability maps extracted from the binary models, and fragment Contribution Maps for the Regression Model together with predicted IC_50_ values.^[67]^ The fragment Contribution Maps for the Regression Model and the probability maps extracted from the binary models are shown in Table S7. In the predicted probability maps, the green atoms/fragments indicate a contribution towards the blockage of hERG, the pink atoms/fragments contribute to the decrease of hERG blockage, and the grey means no contribution.^[67]^ The hybrid compounds containing vitamin D2, such as **18**, **19**, and **20**, were predicted to block hERG K+ channels. However, the compounds, **4**, **7**, **11**, **13**, and **15** were predicted to be non‐blockers of hERG K+ channels.

### 2.7 *In Vitro* Cytotoxicity Assay of the Compounds

The cell viability for **4**, **7**, and **19** revealed toxic effects against the colorectal cancer cells. They were conducted in varying concentrations of 100 μg/mL, 50 μg/mL, 25 μg/mL, 12.5 μg/mL, and 6.25 μg/mL as shown in Table 2. The growth inhibitory effects at 100 μg/mL displayed good concentrations for all selected compounds. However, **19** displayed a significant inhibition effect than compounds **4** and **7** at 50 μg/mL and 100 μg/mL when compared to the parent drug, vitamin D. The IC_50_ value of **19** was 62.3 μg/mL, which was significant when compared to **4** and **7** (Table 2). Colorectal cancer metastasis to the bone is not common and has been reported in only 6–10 % of cases. However, recently, there has been a reported increase in cases of bone metastasis from colorectal cancer.[^68^, ^69^] Bone metastasis resulting from colorectal cancer affects the survival rate of the patients, with a median survival time of five to thirteen months.^[69]^ Bone metastasis due to colorectal cancer occurs mostly in the spine and the pelvis or hips.^[68]^ Bisphosphonate has been reported to be effective when administered to cancer patients with bone metastases, resulting in delayed skeletal‐related events.^[70]^ The anti‐metastatic effects of oxythiamine, an analogue of thymine (Vitamin B1) have been reported.^[71]^ Vitamin D has an antiproliferative effect and suppresses tumour metastasis and angiogenesis.[^72^, ^73^] The design of hybrid compounds containing a combination of anticancer drugs and pamidronate, vitamin D2, or Vitamin B1 is a promising approach to developing effective anticancer drugs to treat cancer that can metastasize to the bone.

**Table 2:** Anticancer activity of the selected compound (**4, 7, 19**) against HT‐29 cell lines.

Compounds	% Viability					1 C50 (μg/mL)
	6.25 μg/mL	12.5 μg/mL	25 μg/mL	50 μg/mL	100 μg/mL	–
4	75.45838	66.89329	138.6372	65.36956	58.2559	118
7	111.4532	87.97642	112.3503	74.63331	59.89005	125.02
19	77.19797	75.49189	112.8483	51.26488	43.89068	62.3
Alendronate	145.1377	111.1793	231.5063	90.39642	88.98565	–
Vitamin D	74.78048	72.75136	84.40247	73.54453	56.96499	–
Cisplatin (Positive control)						44.97

**Figure 2:**
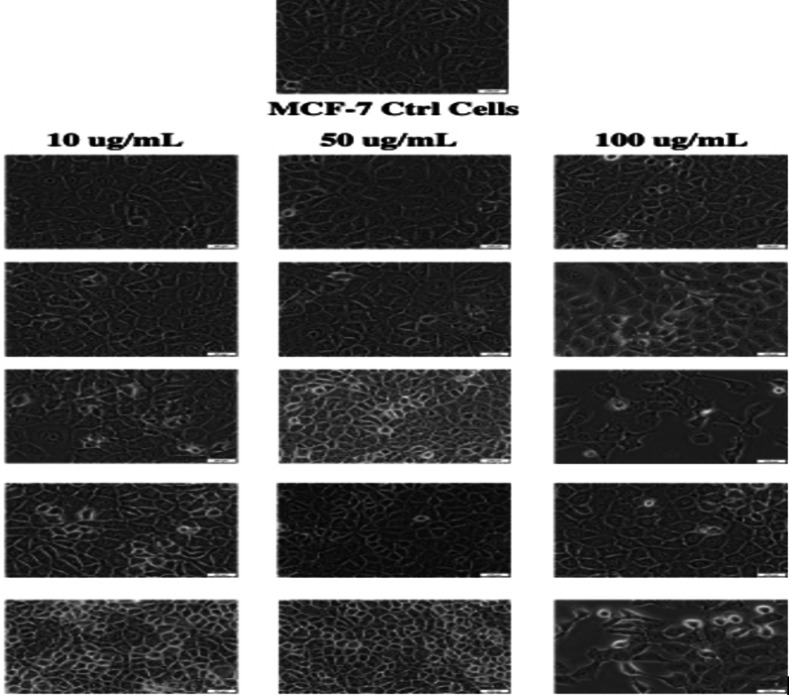
Morphology of MCF‐7 after treatment at different concentrations of the hybrid compounds (**11, 13, 15, 18, 20**).

The cell viability studies of selected hybrid using MCF‐7 cell lines showed that **15** was cytotoxic at a concentration of 100 μg/mL, revealing a promising anticancer activity. However, more studies are needed to fully understand the mode of action of the hybrid compounds (Table 3). The morphology of the cell lines after treatment with the hybrid **20** at the concentration of 100 μg/mL revealed apoptotic cell features, confirming the cytotoxic effect (Figure 2). Similar findings were reported by Majidzadeh et al. for cancer cell lines treated with a combination of two anticancer agents.^[74]^

**Table 3:** Anticancer activity of the selected compound (**11, 13, 15, 18, 20**) against MCF‐7 cell line.

Compounds	% Viability		
	10 μg/mL	50 μg/mL	100 μg/mL
11	87.98	102.5	78.74
13	82.40	89.44	70.53
15	90.76	77.86	59.53
18	81.82	99.71	78.23
20	84.89	66.13	68.91
MCF‐7 ctrl cells	100	100	100

### 2.8 Molecular Docking Studies

**Figure 3:**
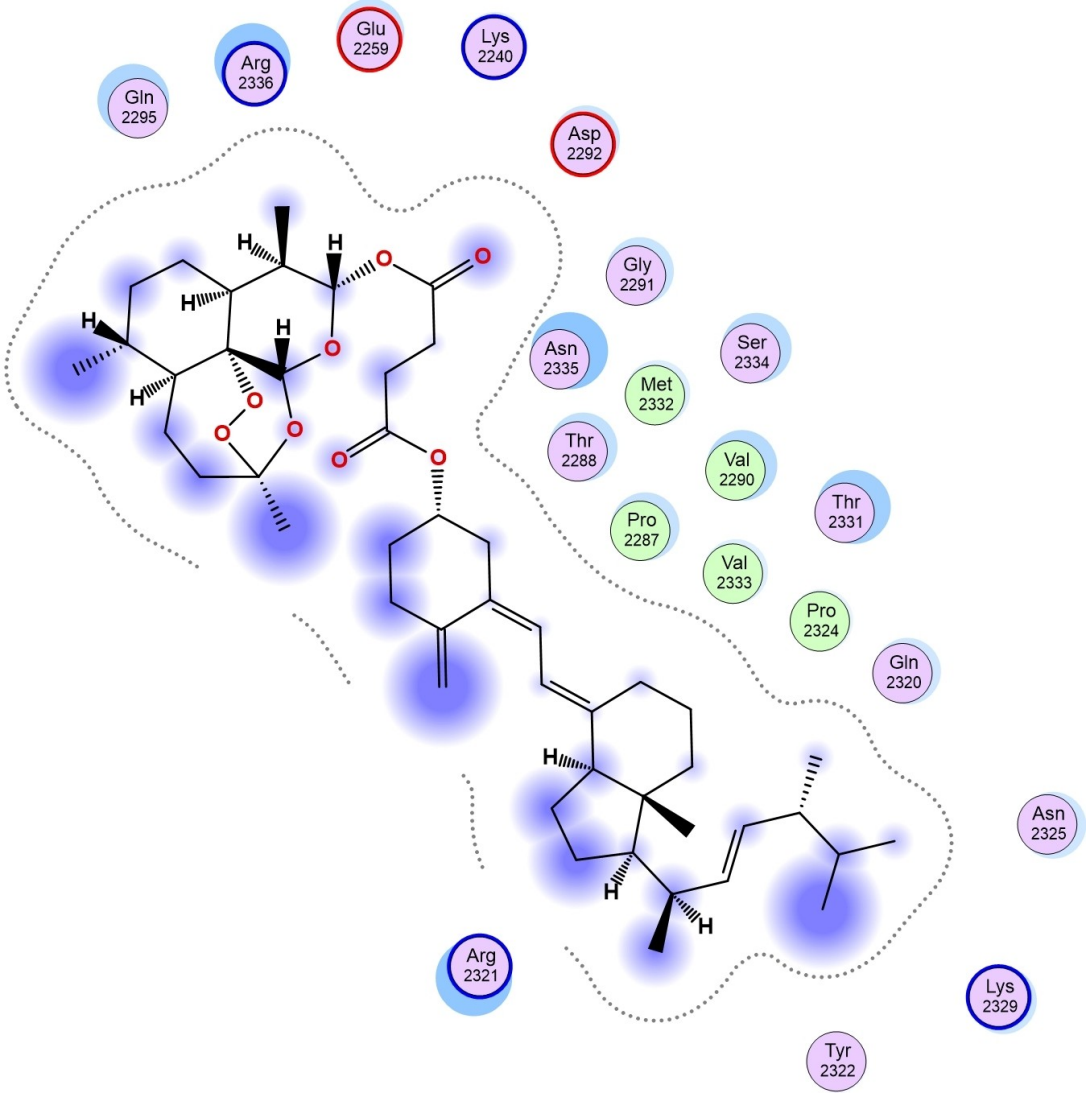
Two‐dimensional structure of **19**‐ human Tyrosine Phosphatase complex.

In this study, selected molecular compounds that were experimentally viable were docked against human Tyrosine Phosphatase and Human 3 alpha‐hydroxysteroid dehydrogenase type 3 to investigate the amino acid residues and the type of non‐bonding interactions that occurred between the studied complexes. Representative 3D structure of the receptor: Human 3 alpha‐hydroxysteroid dehydrogenase type 3 and the selected molecular compound are shown in Figure S25. Compounds **4**, **7**, and **19** were docked against human Tyrosine Phosphatase, and the calculated scoring were −3.35 kcal/mol, −8.42 kcal/mol and −8.64 kcal/mol, respectively (Table 4). The calculated binding affinity for the docked **11**, **13**, **15**, **18**, and **20** against Human 3 alpha‐hydroxysteroid dehydrogenase type 3 was −8.36 kcal/mol, −9.28 kcal/mol, −9.70 kcal/mol, −7.02 kcal/mol and −7.13 kcal/mol, respectively (Table 4). The reference compounds proved to possess a higher ability to inhibit human Tyrosine Phosphatase than compound **4**. Oyebamiji et al., 2023 reported that a compound's capability to inhibit receptors is attributed to a low binding affinity value.^[75]^ Compounds **7** and **19** showed a greater tendency to inhibit the target than compound **4** and the reference compounds. Compound **19** exhibited the highest tendency to inhibit human Tyrosine Phosphatase than other studied compounds (Figure 3) and (Table 4).

**Table 4:** Calculated binding affinity for the studied compounds against human Tyrosine Phosphatase.

4	ALA 2354 (A) H‐donor ALA 2354 (A) SER 2171 (A) ALA 2354 (A) H‐Acceptor	−3.35
7	Ligand Receptor Interaction Distance E (kcal/mol) N47 49 NZ LYS 2301 (A) H‐acceptor 3.01 −11.8 O28 30 NH2 ARG 2400 (A) Ionic 3.07 −4.0	−8.42
19	–	−8.64
**Ref1**	Ligand Receptor Interaction Distance E (kcal/mol) O7 7 NZ LYS 2185 (A) H‐acceptor 3.40 −0.5	−6.18
Cisplatin		−3.86

**Figure 4:**
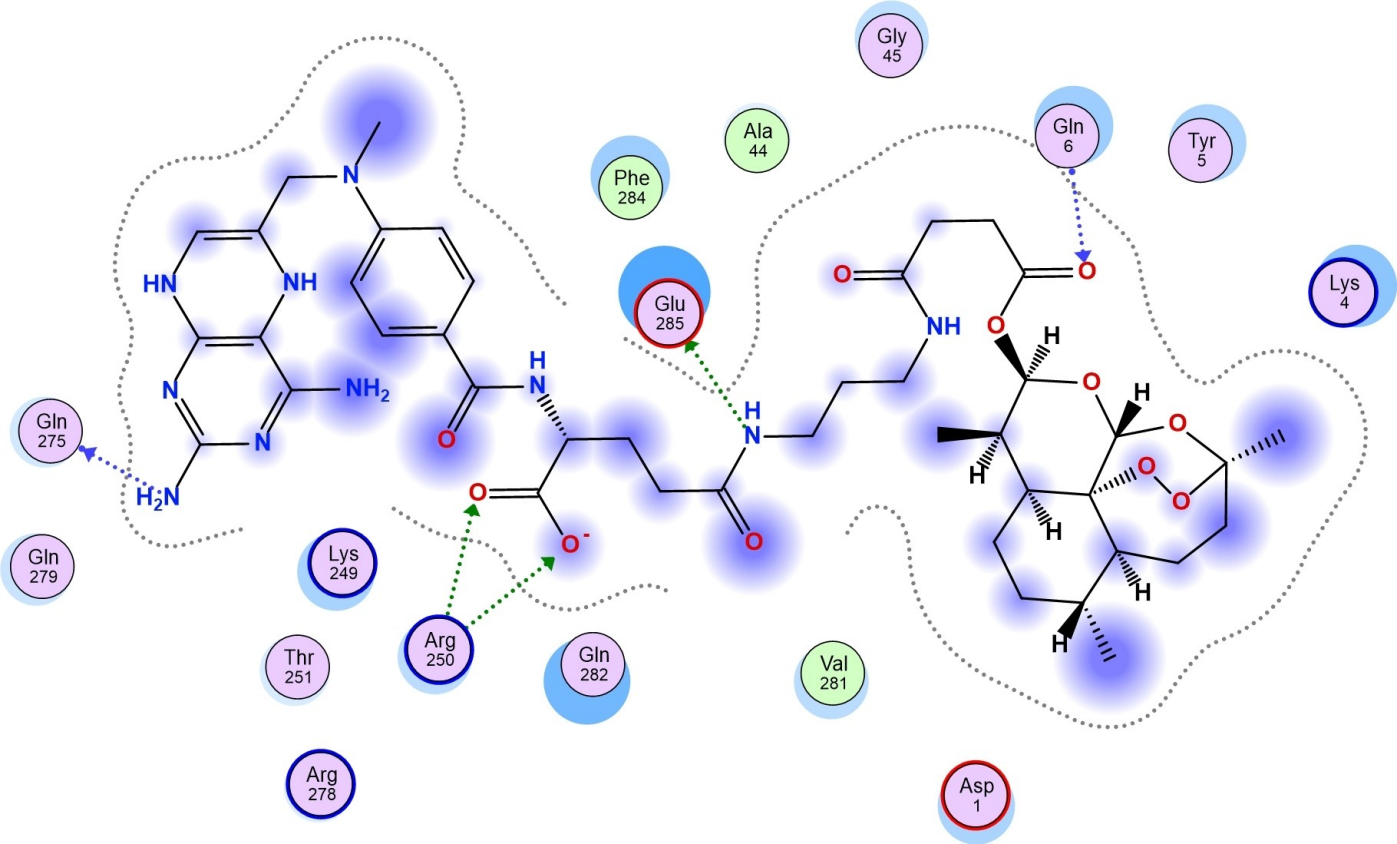
Two‐dimensional structure of **15**‐ Human 3 alpha‐hydroxysteroid dehydrogenase type 3 complex.

Furthermore, the calculated scoring from the docking of compounds **11**, **13**, **15**, **18** and **20** against Human 3 alpha‐hydroxysteroid dehydrogenase type 3 revealed the compound with the highest tendency to inhibit the studied receptor. As shown in Table 5, compound **15** with a calculated binding affinity of −9.70 kcal/mol proved to have the greatest capability to inhibit Human 3 alpha‐hydroxysteroid dehydrogenase type 3 than other studied compounds as well as the referenced compounds used in this research (Figure 4).

**Table 5:** Calculated binding affinity for the studied compounds against Human 3 alpha‐hydroxysteroid dehydrogenase type 3.

11	Ligand Receptor Interaction Distance E (kcal/mol) O27 29 NZ LYS 270 (A) Ionic 3.56 −1.7 O28 30 NZ LYS 270 (A) Ionic 3.00 −4.5 6‐ring CB ALA 27 (A) pi‐H 3.68 −1.0 6‐ring CA HIS 222 (A) pi‐H 4.46 −0.7 6‐ring N LYS 270 (A) pi‐H 4.53 −2.7 6‐ring CB LYS 270 (A) pi‐H 4.05 −0.5	−8.36
13	Ligand Receptor Interaction Distance E (kcal/mol) N40 40 OG SER 217 (A) H‐donor 2.78 −1.4 O23 23 NE ARG 276 (A) H‐acceptor 3.14 −3.0 O23 23 NH2 ARG 276 (A) H‐acceptor 3.37 −1.0 O27 27 NZ LYS 270 (A) H‐acceptor 3.02 −0.9 O27 27 NZ LYS 270 (A) Ionic 3.02 −4.4 6‐ring CB HIS 222 (A) pi‐H 4.22 −0.6	−9.28
15	Ligand Receptor Interaction Distance E (kcal/mol) N11 13 O GLN 275 (A) H‐donor 2.95 −2.1 N31 33 OE2 GLU 285 (A) H‐donor 2.95 −1.5 O27 29 NH2 ARG 250 (A) H‐acceptor 3.11 −6.3 O28 30 NH1 ARG 250 (A) H‐acceptor 2.79 −8.2 O43 45 N GLN 6 (A) H‐acceptor 3.00 −4.2 O27 29 NH1 ARG 250 (A) Ionic 3.41 −2.2 O27 29 NH2 ARG 250 (A) Ionic 3.11 −3.8 O28 30 NH1 ARG 250 (A) Ionic 2.79 −6.0 O28 30 NH2 ARG 250 (A) Ionic 3.97 −0.6	−9.70
18		−7.02
20	Ligand Receptor Interaction Distance E (kcal/mol) 6‐ring N LYS 270 (A) pi‐H 4.00 −0.7	−7.13
^**[Ref1]**^		−7.21
Cisplatin	Ligand Receptor Interaction Distance E (kcal/mol) N 1 O GLN 275 (A) H‐donor 2.86 −3.7 N 18 O ARG 276 (A) H‐donor 2.91 −2.9 O 6 N GLN 282 (A) H‐acceptor 2.86 −1.7 OE1 17 OG1 THR 251 (A) H‐acceptor 2.78 −1.5 O 23 N VAL 283 (A) H‐acceptor 3.20 −1.5	−4.384

### 2.9 Molecular Dynamics Simulation Analysis

**Figure 5:**
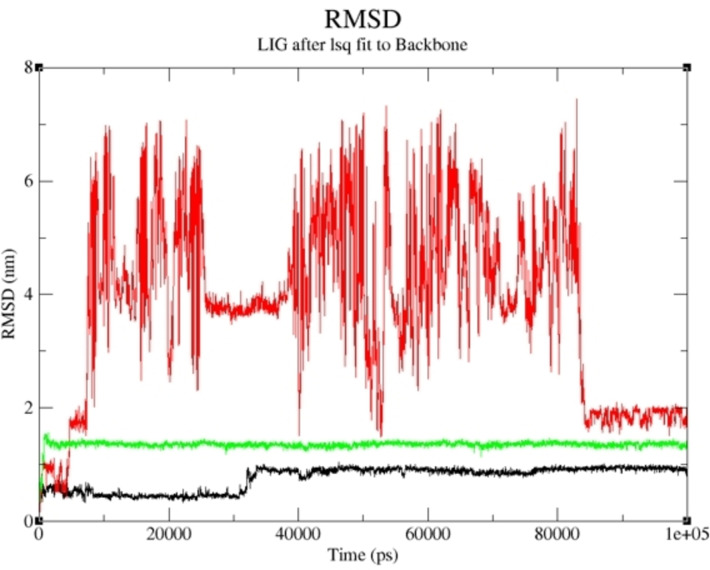
RMSD of Compound **19**‐ human Tyrosine Phosphatase complex and the reference compounds against human Tyrosine Phosphatase during 100 ns simulation.

**Figure 6:**
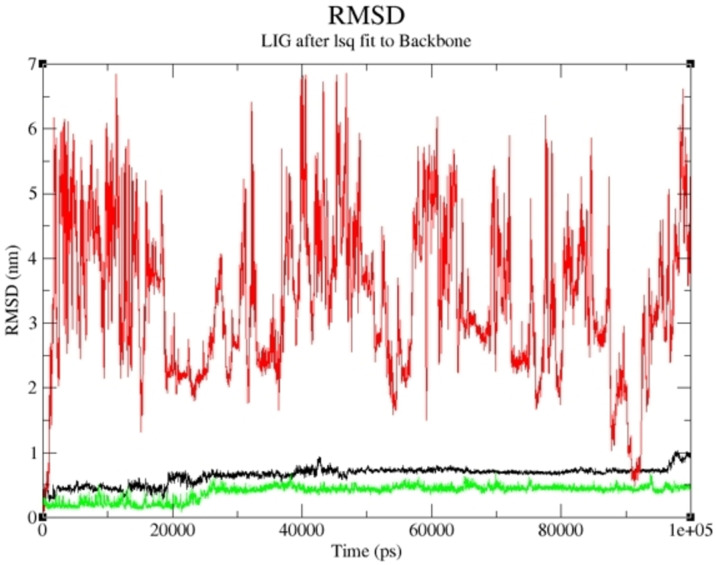
RMSD of Compound **15**‐ Human 3 alpha‐hydroxysteroid dehydrogenase type 3 complex and the reference compounds against Human 3 alpha‐hydroxysteroid dehydrogenase type 3 during 100 ns simulation.

The compounds with the highest binding affinity were subjected to a molecular dynamics simulation study to obtain the actual binding energy between the studied complexes. Compounds **19** and **15** proved potent in inhibiting human Tyrosine Phosphatase and Human 3 alpha‐hydroxysteroid dehydrogenase type 3, respectively. As shown in Figures 5 and 6, the predicted Root Mean Square Deviation (RMSD) using 100 ns simulation time revealed that compounds **19** and **15** were steady compared to the studied reference compounds. This is an indication that **19** and **15** interacted well with the receptor, thereby inhibiting the target than the referenced molecules.

**Figure 7:**
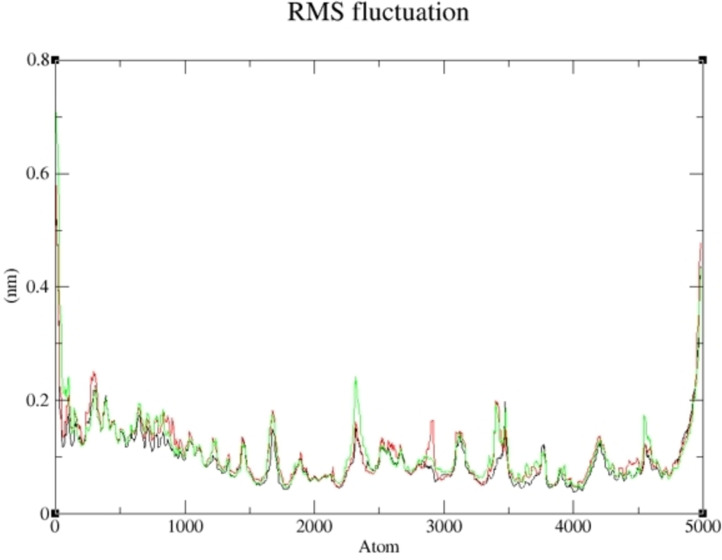
RMSF of Compound **19**‐ human Tyrosine Phosphatase complex and the reference compounds against human Tyrosine Phosphatase during 100 ns simulation.

**Figure 8:**
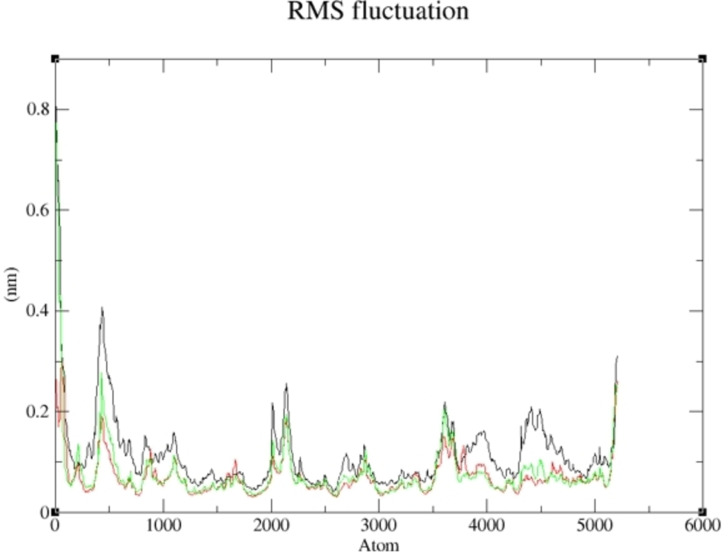
RMSF of Compound **15**‐ Human 3 alpha‐hydroxysteroid dehydrogenase type 3 complex and the reference compounds against Human 3 alpha‐hydroxysteroid dehydrogenase type 3 during 100 ns simulation.

More so, the suppleness of the residues involved in the linking of the selected complexes was investigated *via* Root Mean Square Fluctuation (RMSF). As shown in Figures 7 and 8, a similar configuration was observed for the pattern formed by the selected compounds and the studied receptor except for little deviation; thus, a greater degree of affinity of **19** and **15** toward the studied targets supports their potential greater capacity as anti‐cancer agents. The lower the binding free energy of a compound, the better the capacity of such compound to interact and inhibit the target^[75]^; thus, **19** and **15** proved to be better inhibitors of human Tyrosine Phosphatase and Human 3 alpha‐hydroxysteroid dehydrogenase type 3, respectively. As reported in Tables 6 and 7, the calculated value for van der Waal energy, electrostatic energy, and gas‐phase components were more favourable towards the inhibiting ability compounds **19** and **15** than the reference molecules used in this work.

**Table 6:** Calculated binding Energy Components for 19 and the reference compounds against human Tyrosine Phosphatase.

**Complexes**	**Binding Energy Components (kcal/mol)**				
	**ΔE_vdw_**	**ΔE_ele_**	**ΔG_gas_**	**ΔG_sol_**	**ΔG_bind_**
**Comp19‐1wch**	−37.69± 0.69	−11.8 1.02	−49.49± 1.22	60.21± 1.45	−10.71± 1.09
**Cisplatin‐1wch**	−5.06±0.55	−6.06± 2.3	−11.12±1.98	13.29± 1.62	−2.17± 0.86
**REF‐1wch**	−9.0±0.43	−3.14±0.86	−12.15±1.22	15.99±1.3	3.85±0.54

**Table 7:** Calculated binding Energy Components for 15 and the reference compounds against Human 3 alpha‐hydroxysteroid dehydrogenase type 3.

Complexes	Binding Energy Components (kcal/mol)				
	**ΔE_vdw_**	**ΔE_ele_**	**ΔG_gas_**	**ΔG_sol_**	**ΔG_bind_**
Comp15‐4xo6	−85.47± 2.98	−39.02± 2.14	−124.49± 1.51	140.75± 1.82	−16.26± 3.07
Cisplatin‐4xo6	−10.53±0.85	−18.86± 3.3	−29.39±2.95	29.68± 1.72	0.29± 1.75
REF‐4xo6	−36.52±1.15	−1.74±0.94	−38.27±0.82	39.26± 1.03	1.0± 1.63

## 3 Conclusions

Hybrid Molecules containing Methotrexate, Vitamin D, and bisphosphonates Derivatives are promising compounds for the treatment of cancer that metastasize to the bone. The compounds were synthesized with good yield. They were predicted to be non‐toxic and non‐blockers of hERG K+ channels. Their capability to cause environmental toxicity was not significant. Docking studies revealed compounds **19** and **15** have potent capability to inhibit human Tyrosine Phosphatase and Human 3 alpha‐hydroxysteroid dehydrogenase type 3, respectively. Molecular dynamics studies further showed that compounds **19** and **15** interact well with the receptor, thereby inhibiting the target than the referenced molecules. The Root Mean Square Fluctuation showed a greater degree of affinity of **19** and **15** toward the studied targets, indicating their greater capacity as anti‐cancer agents. The van der Waal energy, electrostatic energy, and gas‐phase components were more favourable towards the inhibiting capability of **19** and **15** than the reference molecules used in this work. Compounds **19** and **15** are promising compounds that require further studies.

## Conflict of Interests

The authors declare no conflict of interest, financial or otherwise.

4

## Supporting information

As a service to our authors and readers, this journal provides supporting information supplied by the authors. Such materials are peer reviewed and may be re‐organized for online delivery, but are not copy‐edited or typeset. Technical support issues arising from supporting information (other than missing files) should be addressed to the authors.

Supporting Information

## Data Availability

The data that support the findings of this study are available in the supplementary material of this article.

## References

[1] G. Ren , M. Esposito , Y. Kang , J. Mol. Med. 2015, 93, 1203–1212. 26275789 10.1007/s00109-015-1329-4 PMC4636917

[2] R. Cathomas , Z. Bajory , M. Bouzid , A. El Ghoneimy , S. Gillessen , F. Goncalves , G. Kacso , G. Kramer , P. Milecki , D. Pacik , W. Tantawy , Urol. Int. 2014, 92, 377–386. 24802278 10.1159/000358258

[3] R. Aft , J. R. Perez , N. Raje , V. Hirsh , F. Saad , Crit. Rev. Oncol. Hematol. 2012, 82, 233–248. 21683613 10.1016/j.critrevonc.2011.05.009

[4] C. G. Anusionwu , B. A. Aderibigbe , S. A. Adeyemi , P. Ubanako , S. O. Oselusi , Y. E. Choonara , X. Y. Mbianda , Bioorg. Med. Chem. 2022, 58, 116652. 35180594 10.1016/j.bmc.2022.116652

[5] C. Chen , Y. Li , X. Yu , Q. Jiang , X. Xu , Q. Yang , Z. Qian , Chin. Chem. Lett. 2018, 29, 1609–1612.

[6] J. Welsh , Arch. Biochem. Biophys. 2012, 523, 107–114. 22085499 10.1016/j.abb.2011.10.019 PMC3295909

[7] A. Pietraszek , M. Malińska , M. Chodyński , M. Krupa , K. Krajewski , P. Cmoch , K. Woźniak , A. Kutner , Steroids. 2013, 78, 1003–1014. 23811018 10.1016/j.steroids.2013.06.001

[8] M. A. Maestro , F. Molnar , C. Carlberg , J. Med. Chem. 2019, 62, 6854–6875. 30916559 10.1021/acs.jmedchem.9b00208 PMC6727385

[9] R. Fernandes , P. Siegel , S. Komarova , J. Hilton , C. Addison , M. F. Ibrahim , J. Werier , K. Dennis , G. Singh , E. Amir , V. Jarvis , J. Bone. Oncol. 2016, 5, 57–62. 27335772 10.1016/j.jbo.2016.02.004 PMC4908181

[10] K. Krüger , J. Thomale , N. Stojanović , M. Osmak , C. Henninger , S. Bormann , G. Fritz , Biochim. Biophys. Acta (BBA)-Mol. Cell. Res. 2015, 1853, 685–698. 10.1016/j.bbamcr.2014.12.033 25565603

[11] R. A. Nadar , K. Farbod , K. C. der Schilden , L. Schlatt , B. Crone , N. Asokan , A. Curci , M. Brand , M. Bornhaeuser , M. Iafisco , N. Margiotta , Sci. Rep. 2020, 10, 1–2. 32246003 10.1038/s41598-020-62039-2 PMC7125202

[12] P. Koźmiński , P. K. Halik , R. Chesori , E. Gniazdowska , Int. J. Mol. Sci. 2020, 21, 3483. 32423175 10.3390/ijms21103483 PMC7279024

[13] B. Álvarez-González , M. Rozalen , M. Fernández-Perales , M. A. Álvarez , M. Sánchez-Polo , Molecules 2020, 25, 6049. 33371436 10.3390/molecules25246049 PMC7767463

[14] S. W. Kmiecik , M. A. Krzyścik , B. Filip-Psurska , J. Wietrzyk , J. Boratyński , T. M. Goszczyński , Adv. Hyg. Exp. Med. 2017, 71, 618–623. 10.5604/01.3001.0010.3842 28791956

[15] B. Cai , A. Liao , K. K. Lee , J. S. Ban , H. S. Yang , Y. J. Im , C. Chun , Steroids. 2016, 116, 45–51. 27770617 10.1016/j.steroids.2016.10.006

[16] S. A. Nadhum , M. H. Mohammed , Iraqi. J. Pharm. Sci. 2015, 24, 74–84.

[17] O. M. Semenenko , V. V. Lipson , A. O. Sadchenko , O. V. Vashchenko , N. A. Kasian , L. V. Sviechnikova , L. M. Lisetski , M. L. Babak , V. M. Vakula , O. V. Borysov , Y. V. Holota , Steroids. 2024, 201, 109332. 37939980 10.1016/j.steroids.2023.109332

[18] Y. Li , C. Zhao , J. Zhang , S. Zhai , B. Wei , L. Wang , J. Chem. Inf. Model. 2019, 59, 4063–4069. 31524396 10.1021/acs.jcim.9b00314

[19] J. M. Delou , A. S. Souza , L. C. Souza , H. L. Borges , Cells 2019, 8, 1013. 31480389 10.3390/cells8091013 PMC6770082

[20] S. A. Desai , A. Manjappa , P. Khulbe , J. Egypt. Natl. Canc. Inst. 2021, 33, 1–4. 33555490 10.1186/s43046-021-00059-3 PMC13316917

[21] P. M. Njogu , J. Gut , P. J. Rosenthal , K. Chibale , ACS Med. Chem. Lett. 2013, 4, 637–641. 24900723 10.1021/ml400164t PMC4027494

[22] L. Pasqua , I. E. De Napoli , M. De Santo , M. Greco , E. Catizzone , D. Lombardo , Nanoscale. Adv. 2019, 1, 3269–3278. 36133588 10.1039/c9na00249a PMC9417532

[23] H. Nakatake , H. Ekimoto , M. Aso , A. Ogawa , A. Yamaguchi , H. Suemune , Chem. Pharm. Bull. 2011, 59, 710–713. 10.1248/cpb.59.710 21628905

[24] C. W. Wu , H. C. Liu , Y. L. Yu , Y. T. Hung , C. W. Wei , G. T. Yiang , Oncol. Rep. 2017, 37, 2177–2184. 28259996 10.3892/or.2017.5439

[25] B. A. Aderibigbe , A. Mugogodi , M. Nwamadi , S. S. Ray , V. Steenkamp , M. O. Balogun , W. M. Matshe , J. Inorg. Organomet. Polym. Mater. 2020, 30, 1503–1518.

[26] H. E. Mukaya , R. L. Van Zyl , N. J. Van Vuuren , X. Y. Mbianda , Polym. Bull. 2017, 74, 3161–3178.

[27] A. Alvarez-Valdes , A. I. Matesanz , J. Perles , C. Fernandes , J. D. Correia , F. Mendes , A. G. Quiroga , J. Inorg. Biochem. 2019, 191, 112–118. 30496946 10.1016/j.jinorgbio.2018.11.010

[28] D. Gupta , S. V. Gupta , K. D. Lee , G. L. Amidon , Mol. Pharm. 2009, 6, 1604–1011. 19566080 10.1021/mp900084v PMC3496387

[29] J. K. Laha , K. V. Patel , K. S. Tummalapalli , M. K. Hunjan , ACS Omega 2018, 3, 8787–8793. 31459011 10.1021/acsomega.8b00894 PMC6645305

[30] V. Khwaza , O. O. Oyedeji , S. O. Oselusi , E. Morifi , M. Nwamadi , D. T. Ndinteh , P. Ramushu , T. Matsebatlela , B. A. Aderibigbe , Chem. Biodivers. 2023, 20, e202300034. 36920086 10.1002/cbdv.202300034

[31] Z. Mbese , M. Nell , Y. T. Fonkui , D. T. Ndinteh , V. Steenkamp , B. A. Aderibigbe , Recent. Adv. Anti-Infect. Drug. Discov. 2022, 17, 54–68. 10.2174/1574891X16666220124122445 35078393

[32] F. Villa , M. Deak , G. B. Bloomberg , D. R. Alessi , D. M. F. Van Aalten , J. Biol. Chem. 2005, 280, 8180. 15611135 10.1074/jbc.M412211200

[33] B. Zhang , X. J. Hu , X. Q. Wang , J. F. Theriault , D. W. Zhu , P. Shang , F. Labrie , S. X. Lin , Biochem. J. 2016, 473, 1037–1046. 26929402 10.1042/BCJ20160083

[34] U. A. Cevik , I. Celik , A. Isik , R. R. Pillai , T. E. Tallei , R. Yadav , Y. Ozkay , Z. A. Kaplancikli , J. Mol. Struct. 2022, 15, 1252.

[35] D. R. Roe , T. E. Cheatham III , J. Chem. Theory. Comput. 2013, 9, 3084–3095. 26583988 10.1021/ct400341p

[36] N. L. Dlamini , H. E. Mukaya , R. L. Van Zyl , N. C. Jansen van Vuuren , X. Y. Mbianda , Artif. Cells. Nanomed. Biotechnol. 2018, 46, 287–296. 10.1080/21691401.2018.1491481 30648446

[37] L. Qiu , G. Lv , Y. Cao , L. Chen , H. Yang , S. Luo , M. Zou , J. Lin , J. Biol. Inorg. Chem. 2015, 20, 1263–1275. 26531104 10.1007/s00775-015-1305-z

[38] S. Ray , R. Sarkar , A. Chattopadhyay , A. K. Ghosh , Prog. React. Kinet. Mech 2014, 39, 122–136.

[39] S. M. El-Megharbel , R. Z. Hamza , A. A. Gobouri , M. S. Refat , Appl. Organomet. Chem. 2019, 33, e4892.

[40] A. Nemat , I. N. Khan , S. Kalsoom , S. A. Malik , S. Ayub , F. Adnan , M. A. Kamal , M. Iqbal , J. Biomol. Struct. Dyn. 2022, 40, 2865–2877. 33183168 10.1080/07391102.2020.1844053

[41] L. Tang , Y. Zhang , J. Xu , Q. Yang , F. Du , X. Wu , M. Li , J. Shen , S. Deng , Y. Zhao , Z. Xiao , Molecules 2023, 28, 1414. 36771080 10.3390/molecules28031414 PMC9920998

[42] A. Lawal , R. A. Umar , M. G. Abubakar , U. Z. Faruk , U. Wali , J. Pharm. Biomed. Sci. 2012, 24, 6–14.

[43] B. L. Munnik , C. H. Kaschula , C. R. Harding , P. Chellan , Dalton. Trans. 2023, 52, 15786–15797. 37681434 10.1039/d3dt02254d PMC10628858

[44] A. Pietraszek , M. Malińska , M. Chodyński , M. Krupa , K. Krajewski , P. Cmoch , K. Woźniak , A. Kutner , Steroids. 2013, 78, 1003–1014. 23811018 10.1016/j.steroids.2013.06.001

[45] G. Bonzi , S. Salmaso , A. Scomparin , A. Eldar-Boock , R. Satchi-Fainaro , P. Caliceti , Bioconjug. Chem. 2015, 26, 489–501. 25613006 10.1021/bc500614b

[46] M. Karacivi , B. Sumer Bolu , R. Sanyal , Mol. Pharm. 2017, 14, 1373–1383. 28358515 10.1021/acs.molpharmaceut.6b01173

[47] W. Jahnke , C. Henry , ChemMedChem. 2010, 5, 770–776. 20209564 10.1002/cmdc.201000016

[48] M. Janicka , A. Śliwińska , M. Sztanke , K. Sztanke , Int. J. Mol. Sci. 2022, 23, 15887. 36555527 10.3390/ijms232415887 PMC9786067

[49] B. Morak-Młodawska , M. Jeleń , E. Martula , R. Korlacki , Int. J. Mol. Sci. 2023, 24, 6970. 37108135 10.3390/ijms24086970 PMC10138389

[50] K. Tsaioun , B. J. Blaauboer , T. Hartung , ALTEX-Altern. Anim. Exp 2016, 33, 343–358. 10.14573/altex.1610101 27806179

[51] M. Rodriguez-Aller , D. Guillarme , J. L. Veuthey , R. Gurny , J. Drug. Deliv. Sci. Technol. 2015, 30, 342–351.

[52] E. R. Samuels , I. Sevrioukova , Mol. Pharm. 2018, 15, 279–288. 29232137 10.1021/acs.molpharmaceut.7b00957 PMC5859942

[53] A. Finch, P. Pillans, *‘Aust. Prescr*. **2014**, *37*, 137–139.

[54] S. K. Dewanjee , T. Dua , N. Bhattacharjee , A. Das , M. Gangopadhyay , R. Khanra , S. Joardar , M. Riaz , V. De Feo , M. Zia-Ul-Haq , Molecules 2017, 22, 871. 28587082 10.3390/molecules22060871 PMC6152721

[55] P. A. Akinnusi , S. O. Olubode , A. A. Alade , A. A. Ashimi , O. L. Onawola , A. O. Agbolade , A. P. Emeka , S. A. Shodehinde , O. Y. Adeniran , Bioinform. Biol. Insights. 2023, 17, 11779322231167970. 37124131 10.1177/11779322231167970 PMC10134171

[56] P. J. Ameji , A. Uzairu , G. A. Shallangwa , S. Uba , J. Taibah. Univ. Med. Sci. 2023, 18, 1417–1437. 38162870 10.1016/j.jtumed.2023.05.021 PMC10757315

[57] M. D. Shultz , J. Med. Chem. 2018, 62, 1701–1714. 30212196 10.1021/acs.jmedchem.8b00686

[58] P. Kalev , A. Sablina , Anti-Cancer. Agents. Med. Chem. 2011, 11, 38–46. 10.2174/187152011794941172 21288198

[59] Z. Guo , V. Johnson , J. Barrera , M. Porras , D. Hinojosa , I. Hernández , P. McGarrah , D. A. Potter , Cancer. Metastasis Rev. 2018, 37, 409–423. 30066055 10.1007/s10555-018-9749-6

[60] S. Pucci , M. J. Zonetti , T. Fisco , C. Polidoro , G. Bocchinfuso , A. Palleschi , G. Novelli , L. G. Spagnoli , P. Mazzarelli , Oncotarget 2016, 7, 19982. 26799588 10.18632/oncotarget.6964 PMC4991433

[61] A. Hermawan , H. Putri , Egypt. J. Med. Hum. Genet. 2022, 23, 1–7.37521842

[62] K. Zhang , D. Chen , K. Ma , X. Wu , H. Hao , S. Jiang , J. Med. Chem. 2018, 61, 6983–7003. 29712428 10.1021/acs.jmedchem.8b00124

[63] S. D. Mason , J. A. Joyce , Trends. Cell. Biol. 2011, 21, 228–237. 21232958 10.1016/j.tcb.2010.12.002 PMC3840715

[64] S. S. Hassan , W. D. Zhang , H. Z. Jin , S. H. Basha , S. S. Priya , J. Biomol. Struct. Dyn. 2022, 40, 484–498. 32876526 10.1080/07391102.2020.1815579

[65] A. Garrido , A. Lepailleur , S. M. Mignani , P. Dallemagne , C. Rochais , Eur. J. Med. Chem. 2020, 195, 112290. 32283295 10.1016/j.ejmech.2020.112290

[66] S. Kalyaanamoorthy , K. H. Barakat , Med. Res. Rev. 2018, 38, 525–555. 28467598 10.1002/med.21445

[67] R. C. Braga , V. M. Alves , M. F. Silva , E. Muratov , D. Fourches , L. M. Lião , A. Tropsha , C. H. Andrade , Mol. Inform. 2015, 34, 698–701. 27490970 10.1002/minf.201500040 PMC5720373

[68] L. Holladay , J. Luu , V. Balendra , K. Kmetz , Cancer. Treat. Res. Commun. 2023, 37, 100763. 37839182 10.1016/j.ctarc.2023.100763

[69] F. Liu , J. Zhao , J. Xie , L. Xie , J. Zhu , S. Cai , H. Zheng , Y. Xu , Tumor. Biol. 2016, 37, 16127–16134.

[70] T. L. Ng , M. M. Tu , M. F. Ibrahim , B. Basulaiman , S. F. McGee , A. Srikanthan , R. Fernandes , L. Vandermeer , C. Stober , M. Sienkiewicz , A. Jeong , Support. Care. Cancer. 2021, 29, 925–943. 32535678 10.1007/s00520-020-05556-0

[71] C. M. Yang , Y. Z. Liu , J. W. Liao , M. L. Hu , Clin. Exp. Metastasis. 2010, 27, 341–349. 20449639 10.1007/s10585-010-9331-2

[72] J. Xu , W. Li , J. Ma , J. Liu , H. Sha , S. Zhou , F. Wang , Q. Ma , Curr. Med. Chem. 2013, 20, 4109–4120. 23895682 10.2174/09298673113209990194

[73] S. Y. Na , K. B. Kim , Y. J. Lim , H. J. Song , J. Cancer. Prev. 2022, 27, 147. 36258716 10.15430/JCP.2022.27.3.147 PMC9537583

[74] H. Majidzadeh , M. Araj-Khodaei , M. Ghaffari , A. Jafari , D. Shanehbandi , M. Torbati , J. E. Dolatabadi , Pharm. Sci. 2021, 27, 536–542.

[75] A. K. Oyebamiji, G. F. Tolufashe, O. M. Oyawoye, T. A. Oyedepo, B. Semire, *J. Chem*. **2020**, 1–10

