# Supplementary material for: Hybrid Molecules Containing Methotrexate, Vitamin D, and Platinum Derivatives: Synthesis, Characterization, In Vitro Cytotoxicity, In Silico ADME Docking, Molecular Docking and Dynamics
Source: Chem Biodivers. 2024 Nov 7;22(1):e202400373. doi: 10.1002/cbdv.202400373 (PMC11741164; doi:10.1002/cbdv.202400373)
Supplement: Supplementary file 1 — Supporting Information [file CBDV-22-e202400373-s001.pdf]

Chemistry & Biodiversity

Supporting Information

Hybrid Molecules Containing Methotrexate, Vitamin D, and Platinum Derivatives: Synthesis, Characterization, *In Vitro* Cytotoxicity, *In Silico* ADME Docking, Molecular Docking and Dynamics

Zintle Mbese, Mpho Choene, Eric Morifi, M. Nwamadi, Samson Adeyemi, Abel Kolawole Oyebamiji, Adedapo S. Adeyinka, Blassan George, and Blessing Atim Aderibigbe*

Table of Contents

SUPPLEMENTARY FIGURES AND TABLES 3

Figure S1a: FTIR spectrum of **4** 3

Figure S1b: ^{1}H-NMR spectra of **4** 3

Figure S1c: ^{31}P-NMR spectra of **4** 4

Figure S1d: ^{13}C-NMR spectra of **4** 4

Figure S1e: HRMS spectrum of **4** 5

Figure S2a: FTIR spectrum of **7** 5

Figure S2b: ^{1}H-NMR spectra of **7** 6

Figure S2c: ^{13}C-NMR spectra of **7** 6

Figure S2d: HRMS spectrum of **7** 7

Figure S3a: FTIR spectrum of **11** 7

Figure S3b: ^{1}H-NMR spectra of **11** 8

Figure S3c: ^{13}C-NMR spectra of **11** 8

Figure S3d: HRMS spectrum of **11** 9

Figure S4a: FTIR spectrum of **13** 9

Figure S4b: ^{1}H-NMR spectra of **13** 10

Figure S4c: ^{13}C-NMR spectra of **13** 10

Figure S4d: HRMS spectrum of **13** 11

Figure S5a: FTIR spectrum of **15** 11

Figure S5b: ^{1}H-NMR spectra of **15** 12

Figure S5c: ^{13}C-NMR spectra of **15** 12

Figure S5d: HRMS spectrum of **15** 13

Figure S6a: FTIR spectrum of **18** 13

Figure S6b: ^{1}H-NMR spectra of **18** 14

Figure S6c: ^{13}C-NMR spectra of **18** 14

Figure S6d: HRMS spectrum of **18** 15

Figure S7a: FTIR spectrum of **19** 15

Figure S7b: ^{1}H-NMR spectra of **19** 16

Figure S7c: ^{13}C-NMR spectra of **19** 16

Figure S7d: HRMS spectrum of **19** 17

Figure S8a: FTIR spectrum of **20** 17

Figure S8b: ^{1}H-NMR spectra of **20** 18
Figure S8c: ^{13}C-NMR spectra of **20** 18
Figure S8d: HRMS spectrum of **20** 19
Figure S9: BOILED-Egg model of **4** 25
Figure S10: BOILED-Egg model of **7** 25
Figure S11: BOILED-Egg model of **11** 26
Figure S12: BOILED-Egg model of **13** 26
Figure S13: BOILED-Egg model of **15** 27
Figure S14: BOILED-Egg model of **18** 27
Figure S15: BOILED-Egg model of **19** 28
Figure S16: BOILED-Egg model of **20** 28
Figure S17: SWISS target prediction of **4** 29
Figure S18: SWISS target prediction of **7** 29
Figure S19: SWISS target prediction of **11** 30
Figure S20: SWISS target prediction of **13** 30
Figure S21: SWISS target prediction of **15** 31
Figure S22: SWISS target prediction of **18** 31
Figure S23: SWISS target prediction of **19** 32
Figure S24: SWISS target prediction of **20** 32
Table S4: Rat Toxicity Prediction using GUSAR 34
Table S5: Environmental Toxicity using GUSAR 35
Table S6: hERG-predictions from Pred-hERG 36
Table S7: The fragment Contribution Maps for the Regression Model 38
Figure S25: Representative 3D structure of the receptor: Human 3 alpha-hydroxysteroid dehydrogenase type 3 40

SUPPLEMENTARY FIGURES AND TABLES

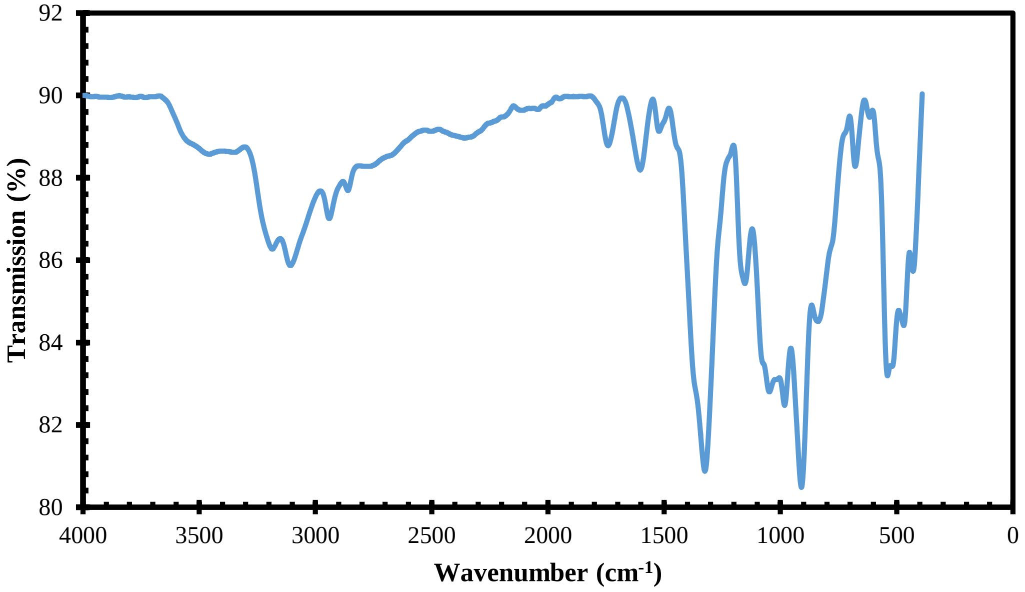

Figure S1a: FTIR spectrum of **4**

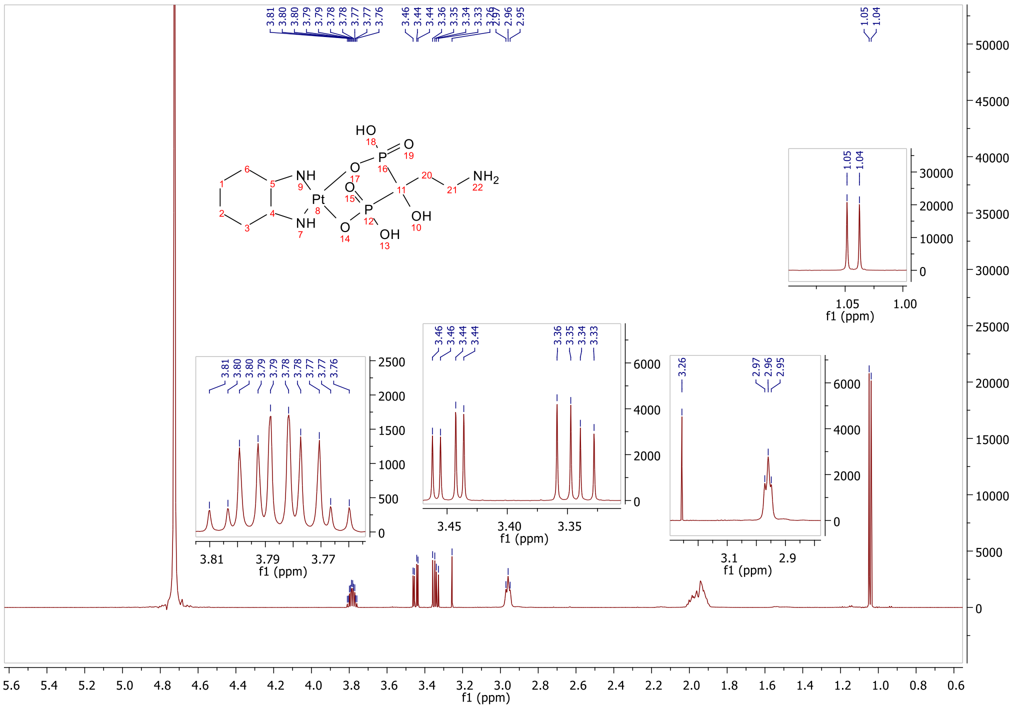

Figure S1b: ^{1}H-NMR spectra of **4**

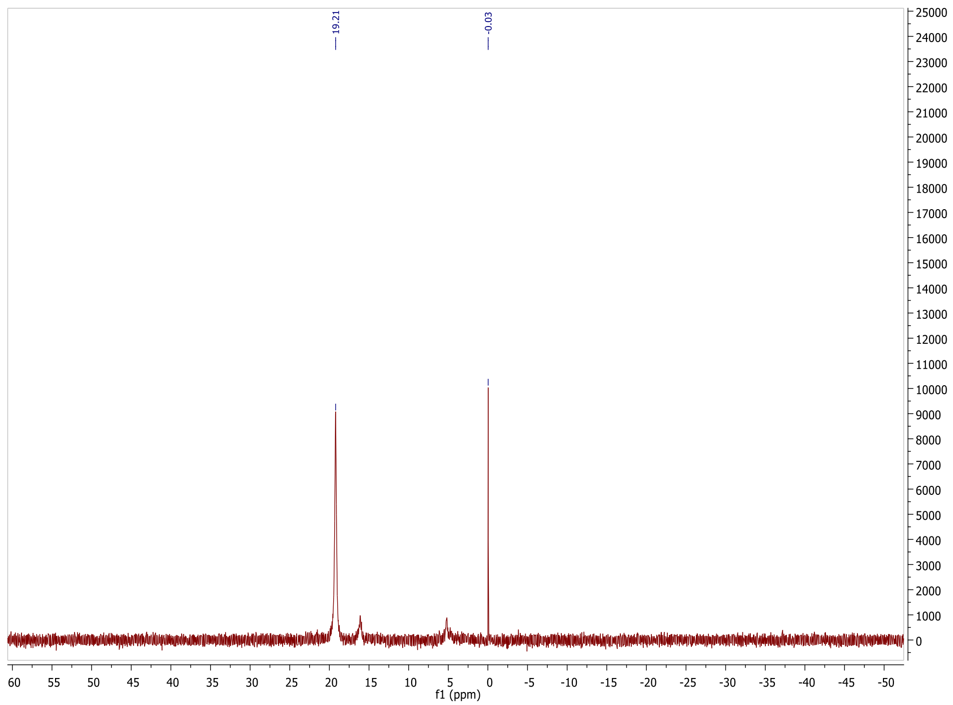

Figure S1c: ^{31}P-NMR spectra of **4**

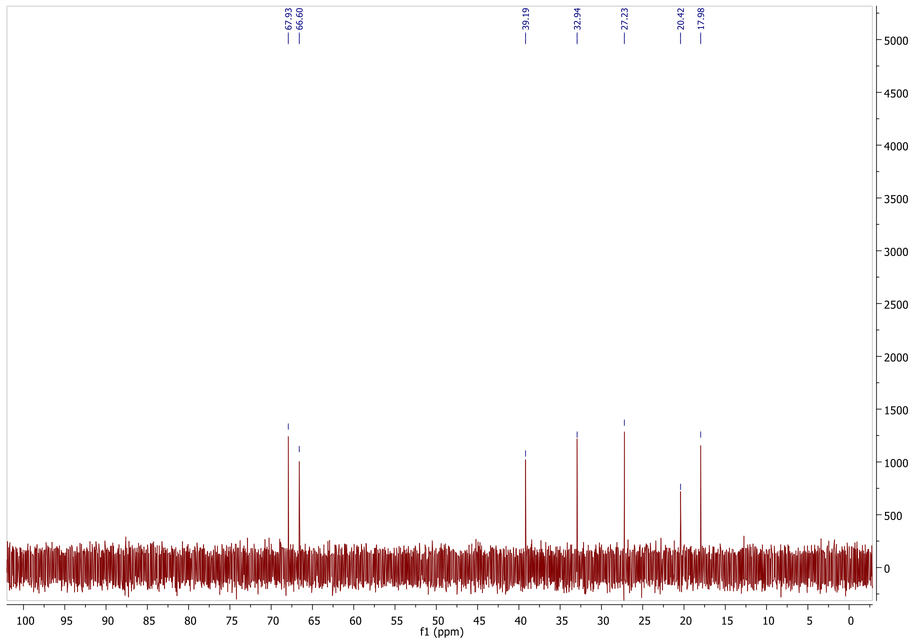

Figure S1d: ^{13}C-NMR spectra of **4**

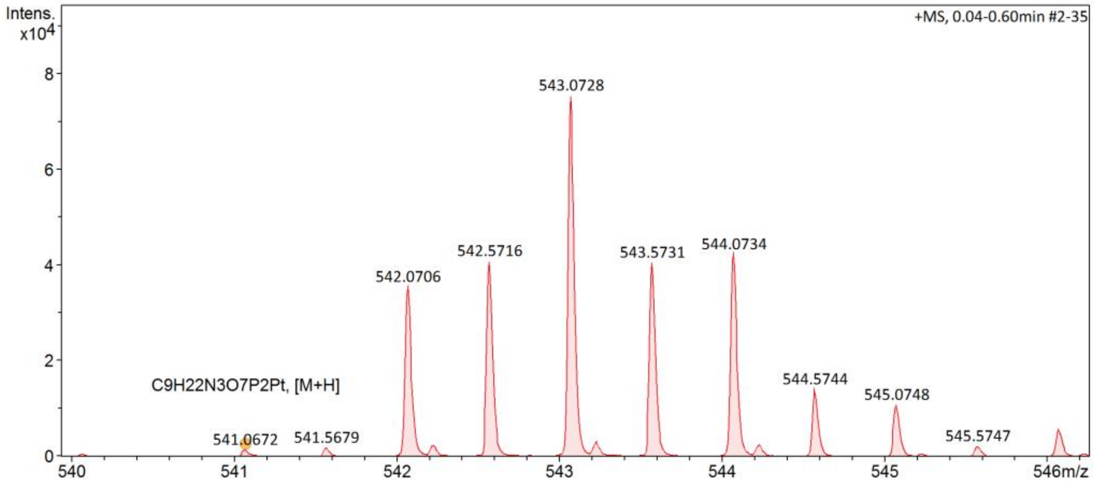

Figure S1e: HRMS spectrum of **4**

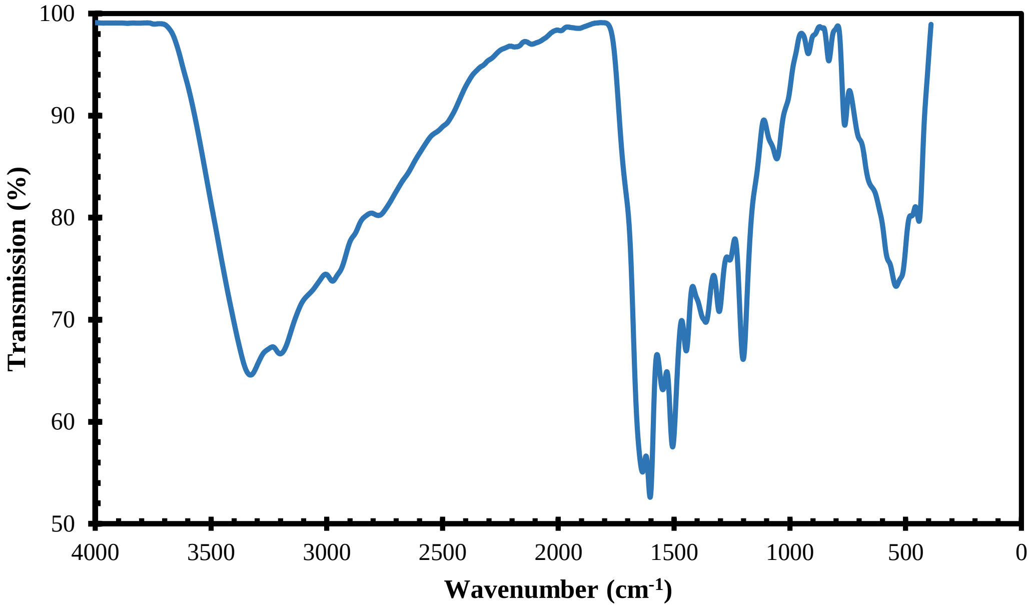

Figure S2a: FTIR spectrum of **7**

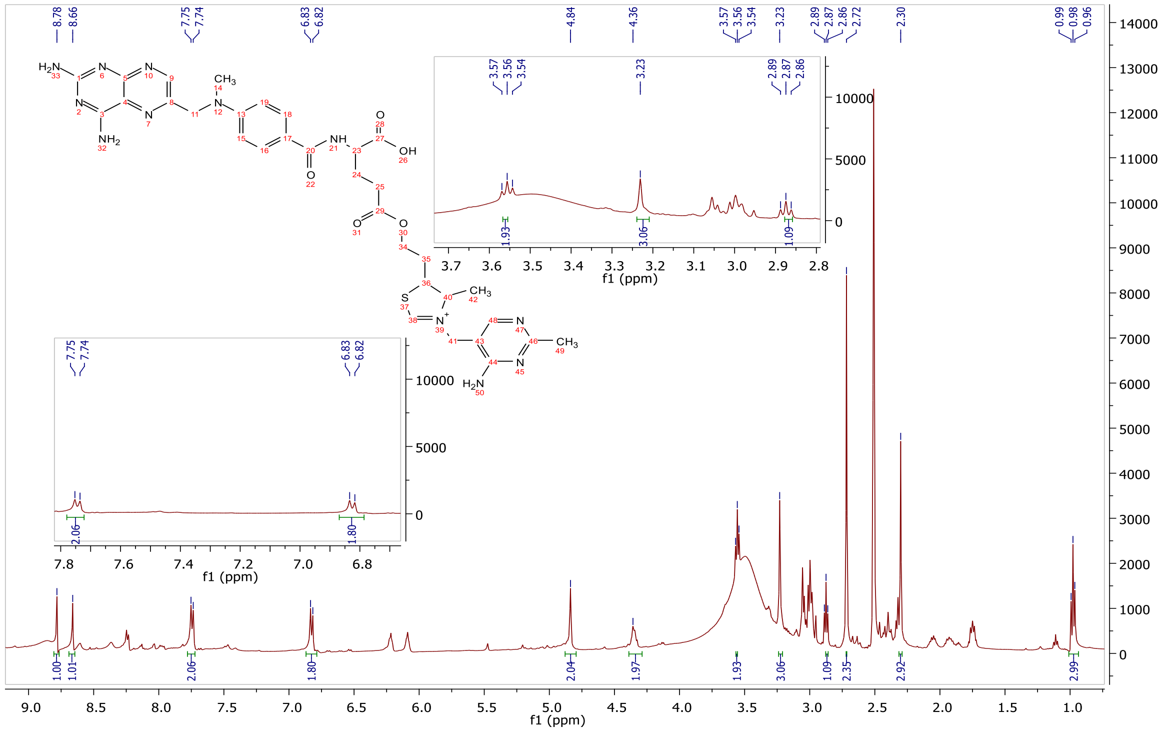

Figure S2b: ^{1}H-NMR spectra of **7**

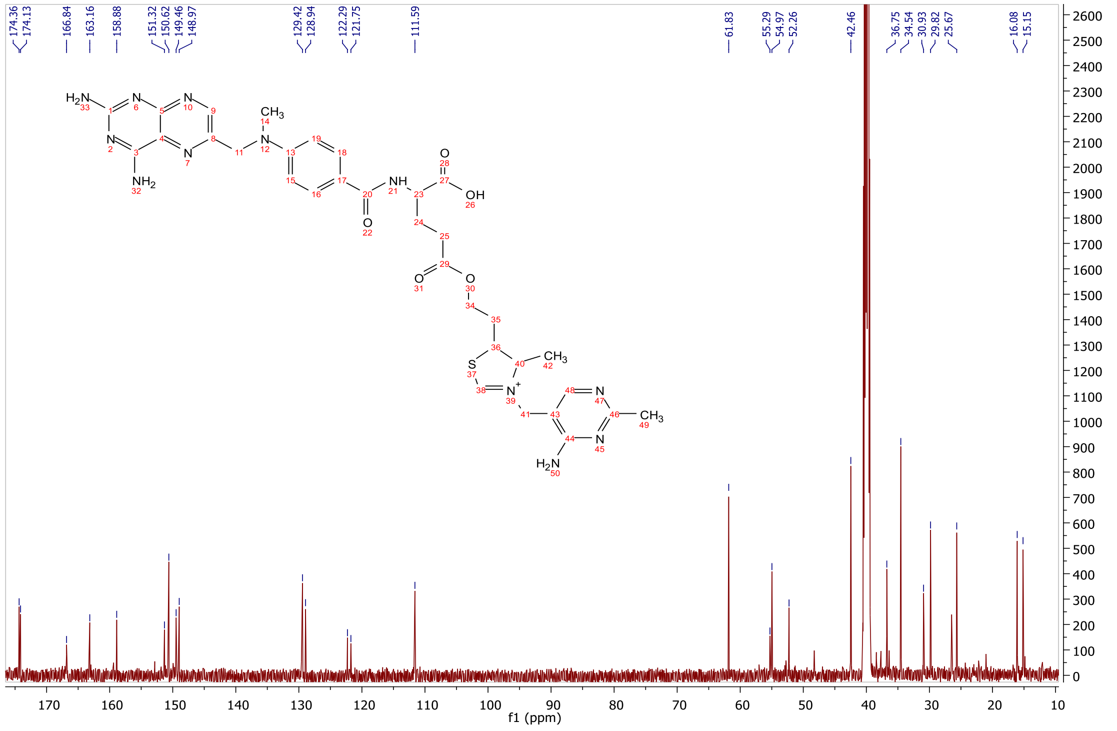

Figure S2c: ^{13}C-NMR spectra of **7**

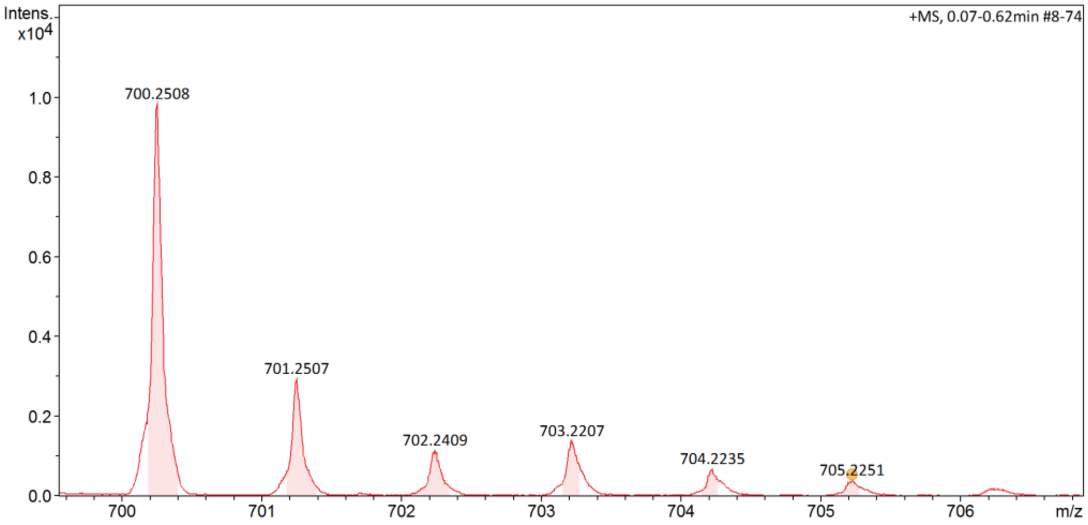

Figure S2d: HRMS spectrum of **7**

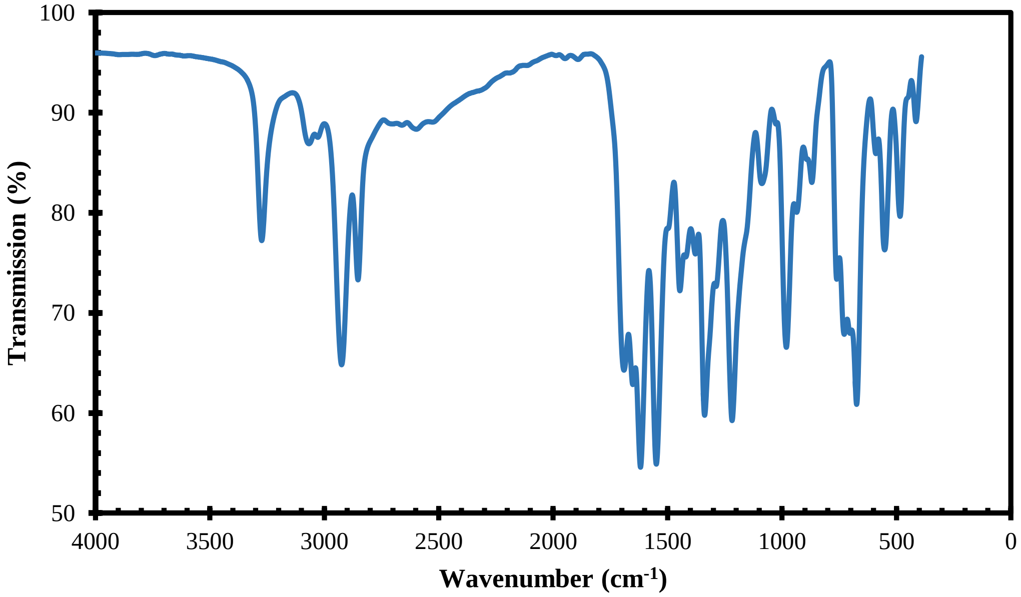

Figure S3a: FTIR spectrum of **11**

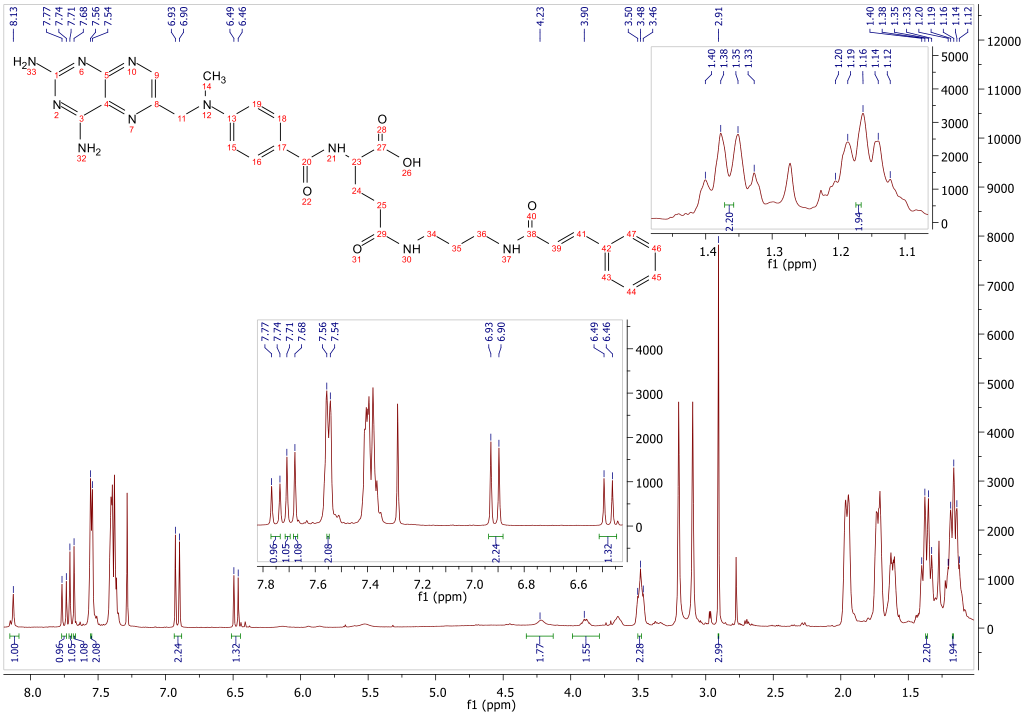

Figure S3b: ^{1}H-NMR spectra of **11**

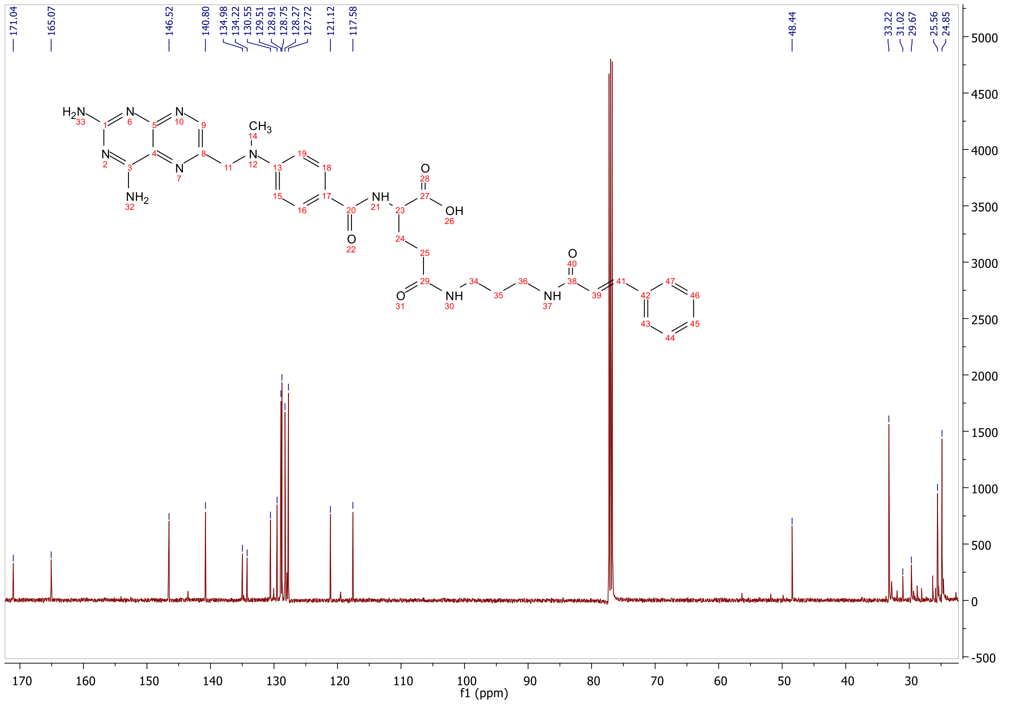

Figure S3c: ^{13}C-NMR spectra of **11**

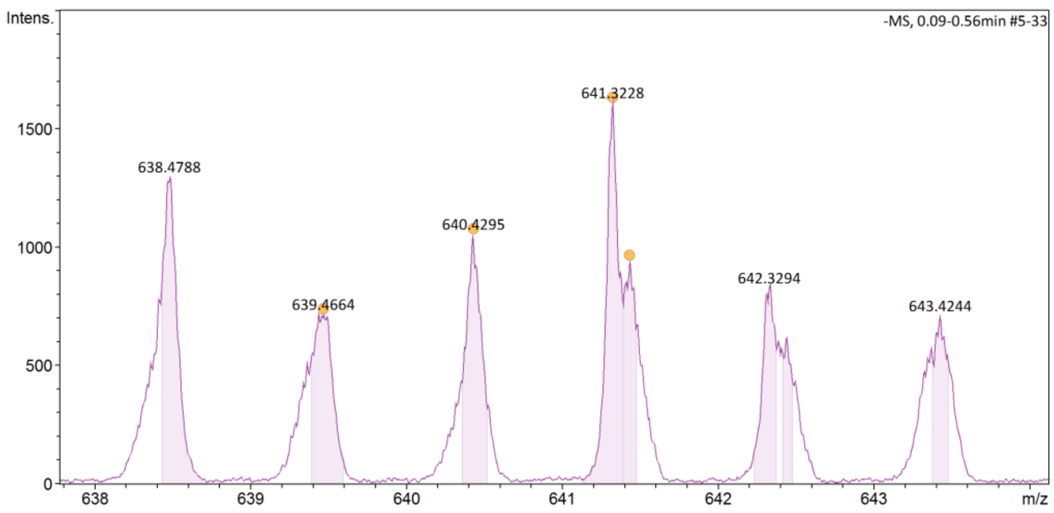

Figure S3d: HRMS spectrum of **11**

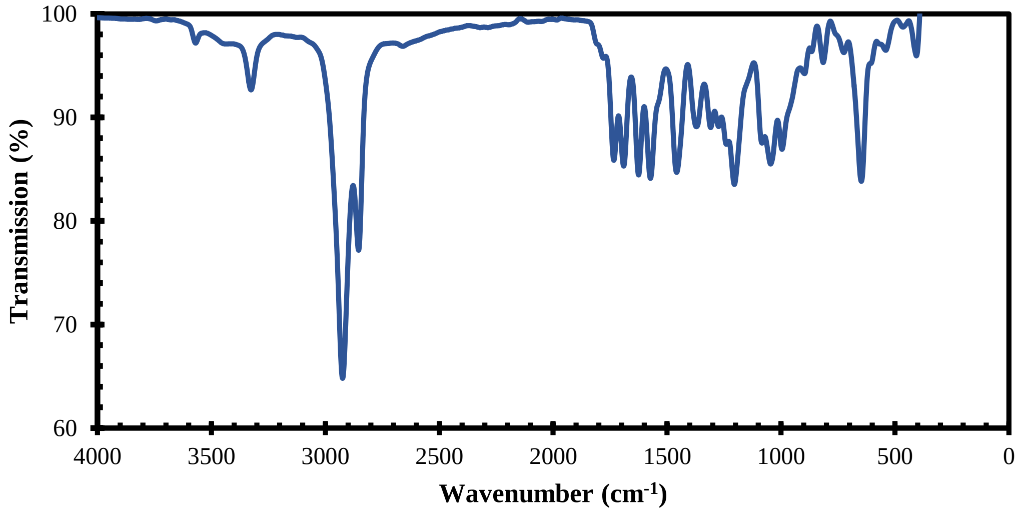

Figure S4a: FTIR spectrum of **13**

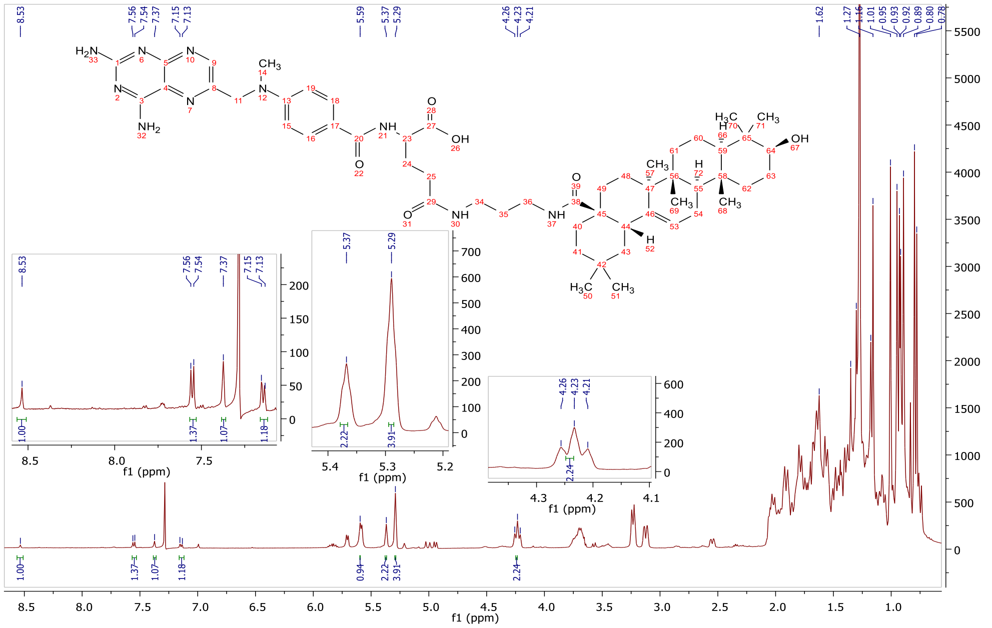

Figure S4b: ^{1}H-NMR spectra of **13**

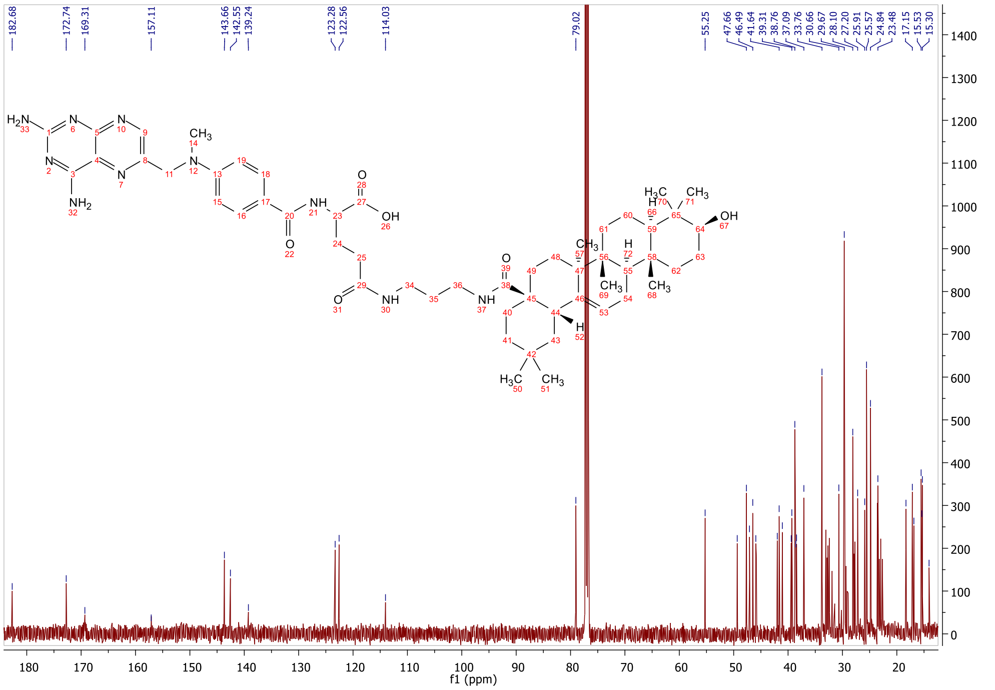

Figure S4c: ^{13}C-NMR spectra of **13**

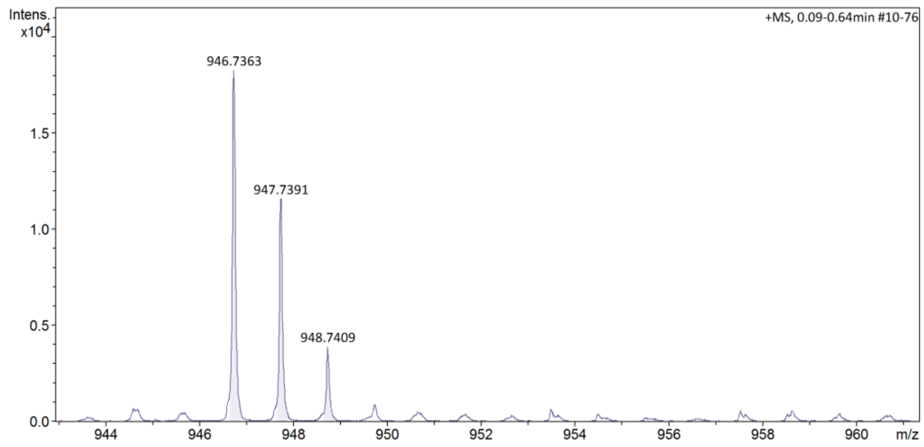

Figure S4d: HRMS spectrum of **13**

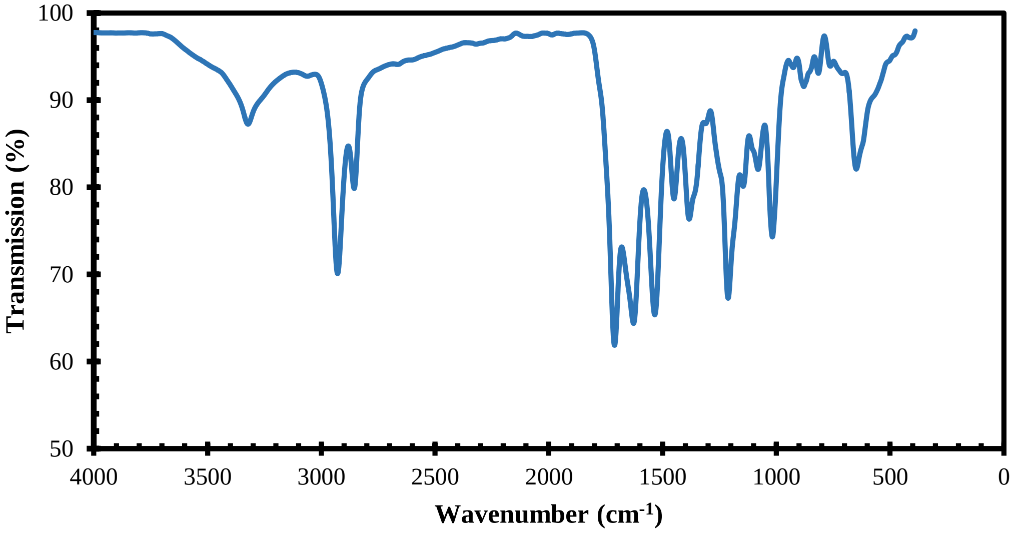

Figure S5a: FTIR spectrum of **15**

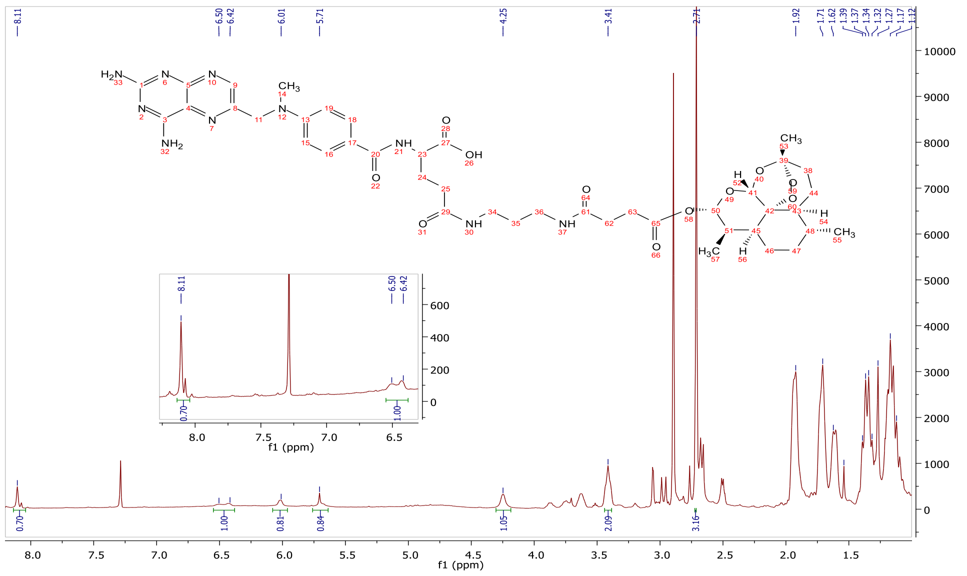

Figure S5b: ^{1}H-NMR spectra of **15**

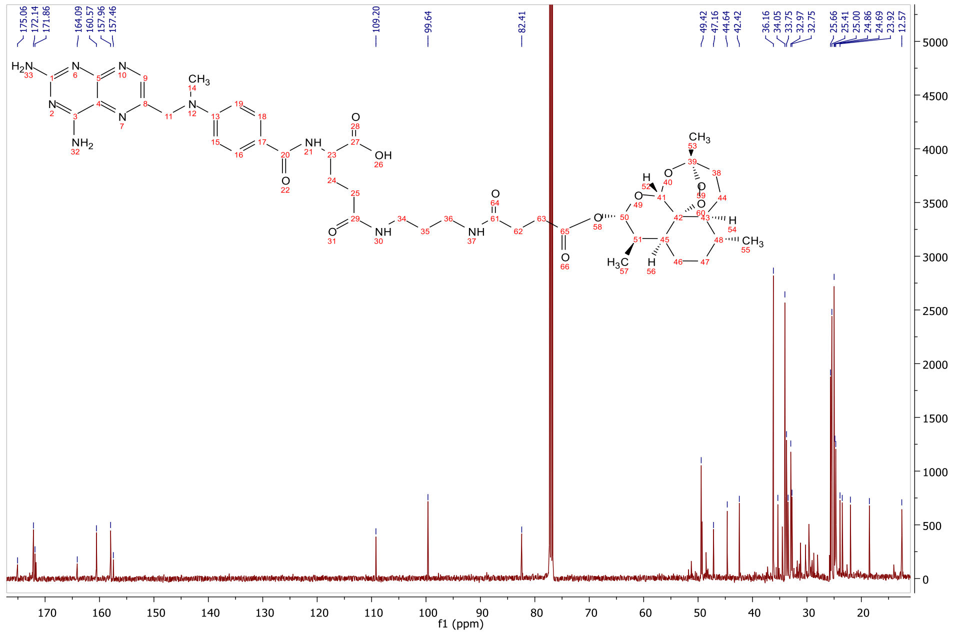

Figure S5c: ^{13}C-NMR spectra of **15**

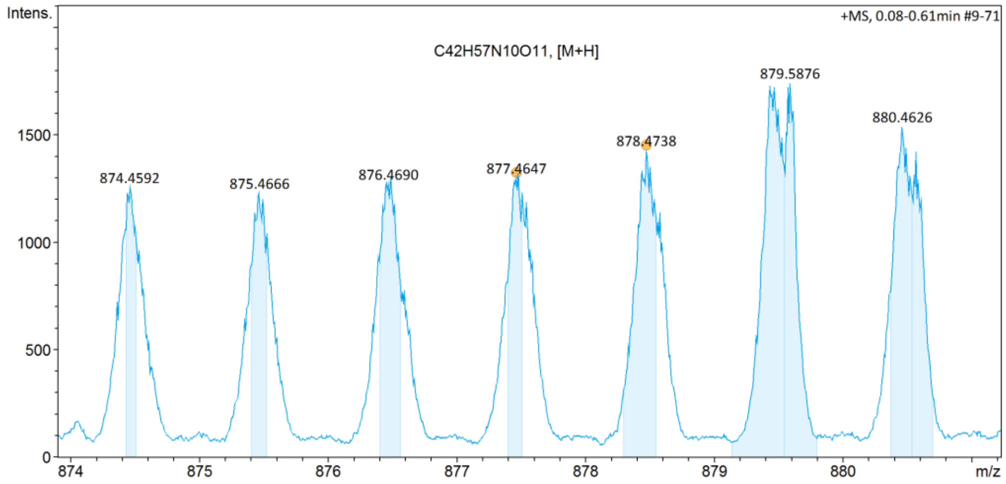

Figure S5d: HRMS spectrum of **15**

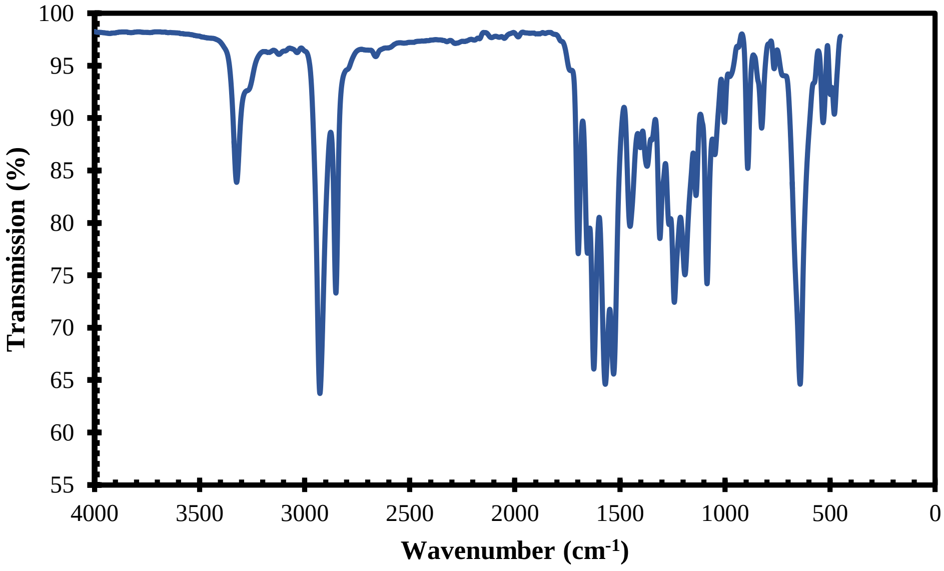

Figure S6a: FTIR spectrum of **18**

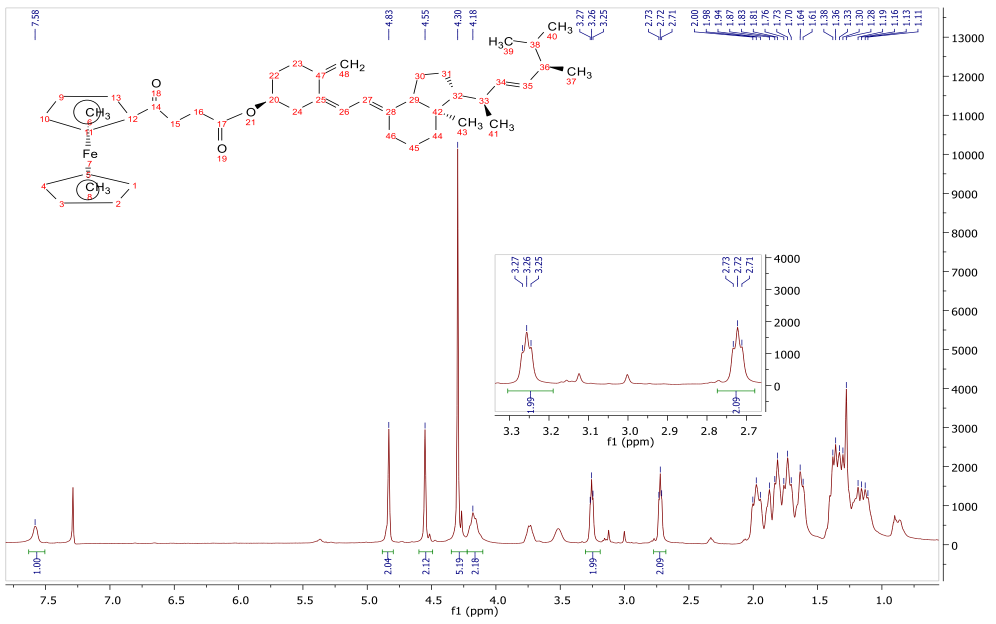

Figure S6b: ^{1}H-NMR spectra of **18**

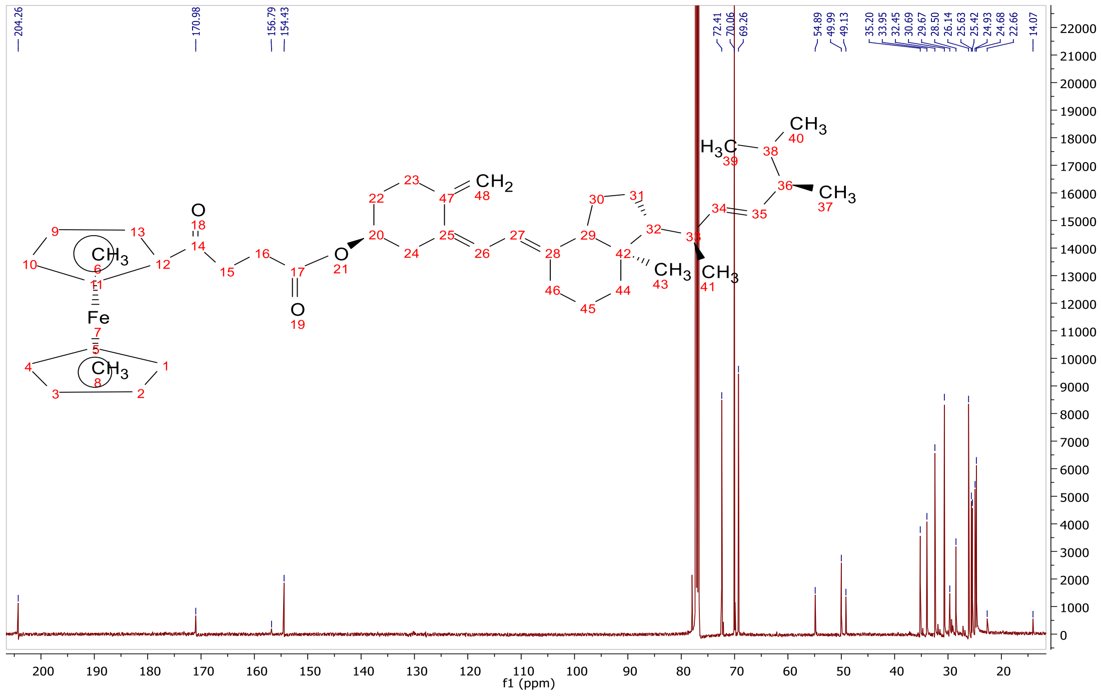

Figure S6c: ^{13}C-NMR spectra of **18**

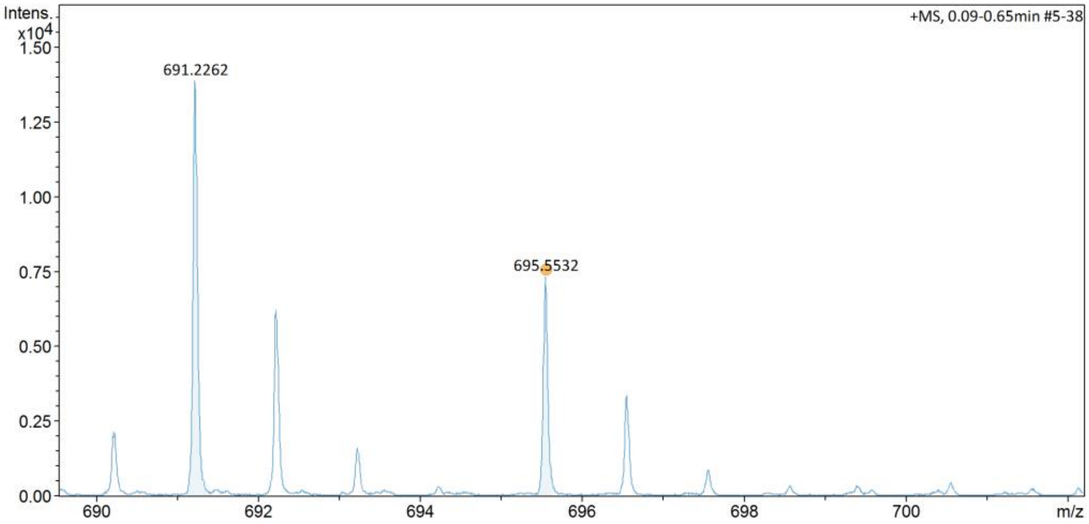

Figure S6d: HRMS spectrum of **18**

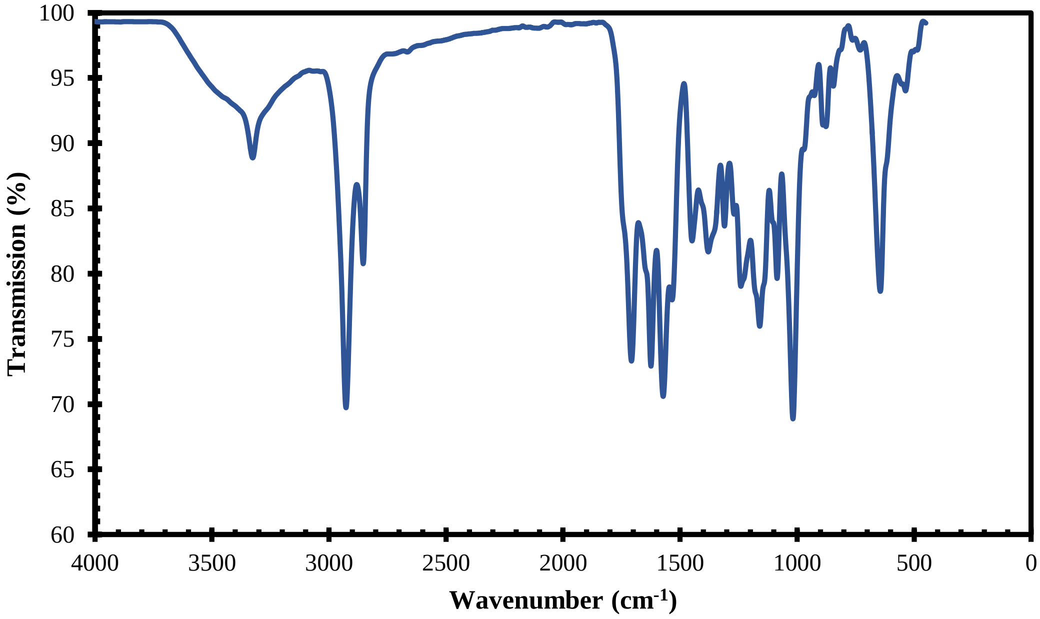

Figure S7a: FTIR spectrum of **19**

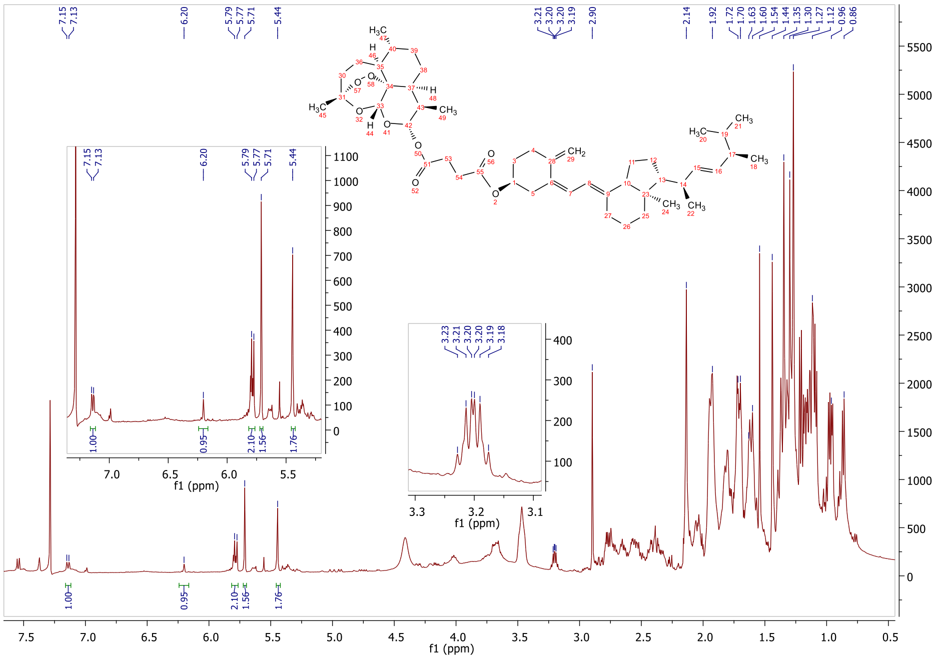

Figure S7b: ^{1}H-NMR spectra of **19**

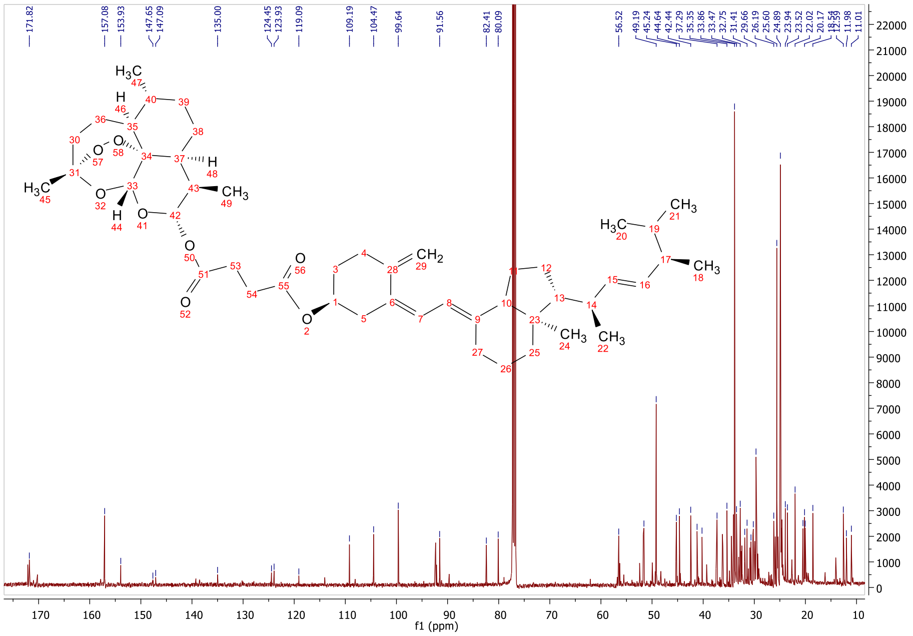

Figure S7c: ^{13}C-NMR spectra of **19**

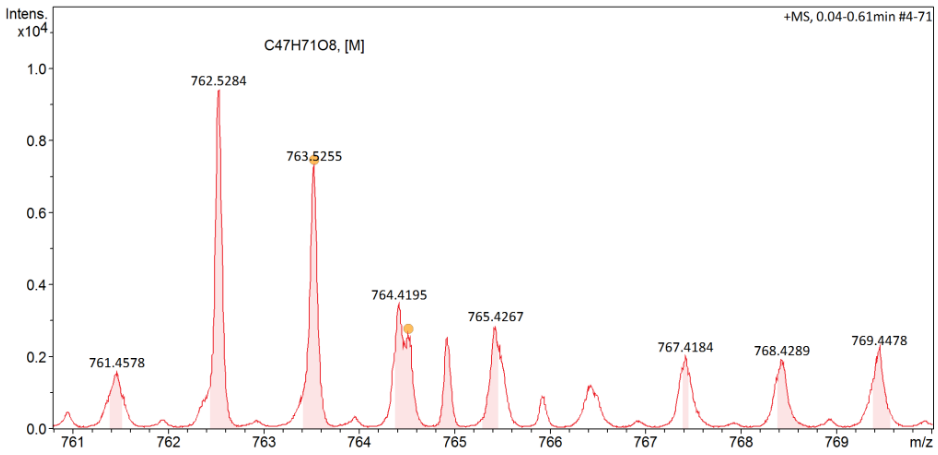

Figure S7d: HRMS spectrum of **19**

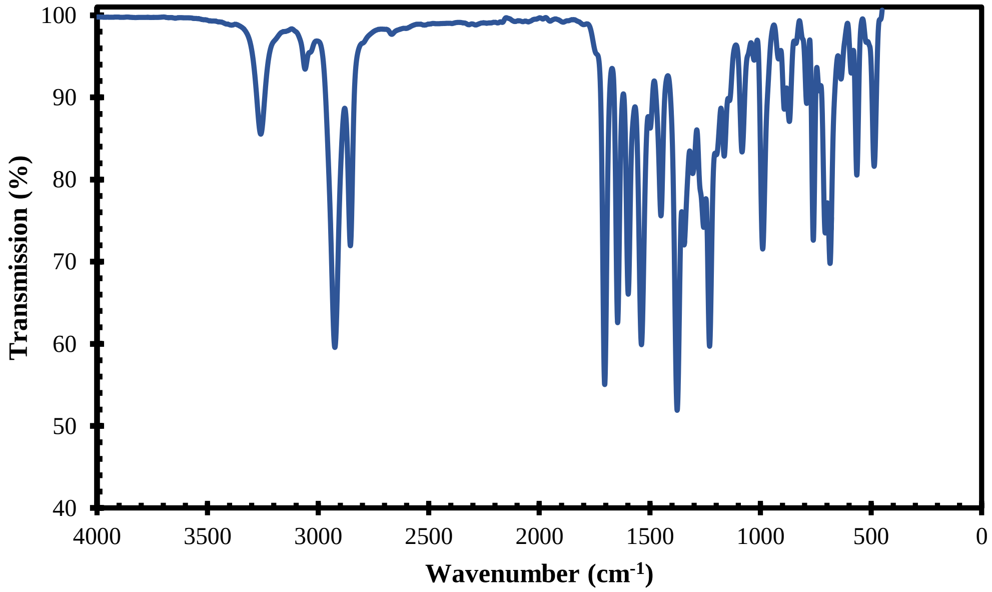

Figure S8a: FTIR spectrum of **20**

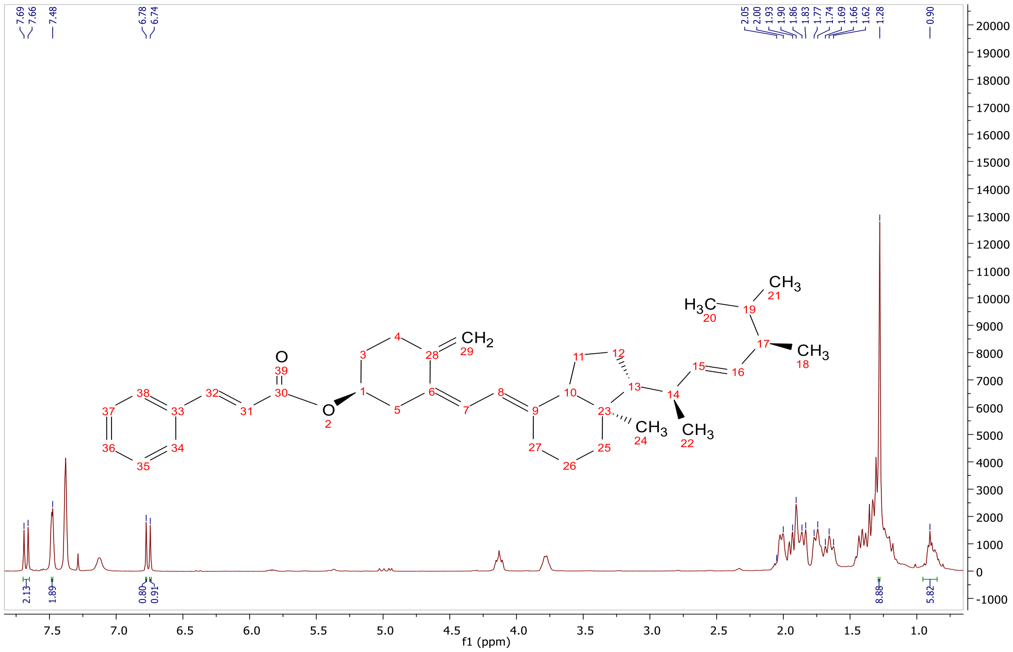

Figure S8b: ^{1}H-NMR spectra of **20**

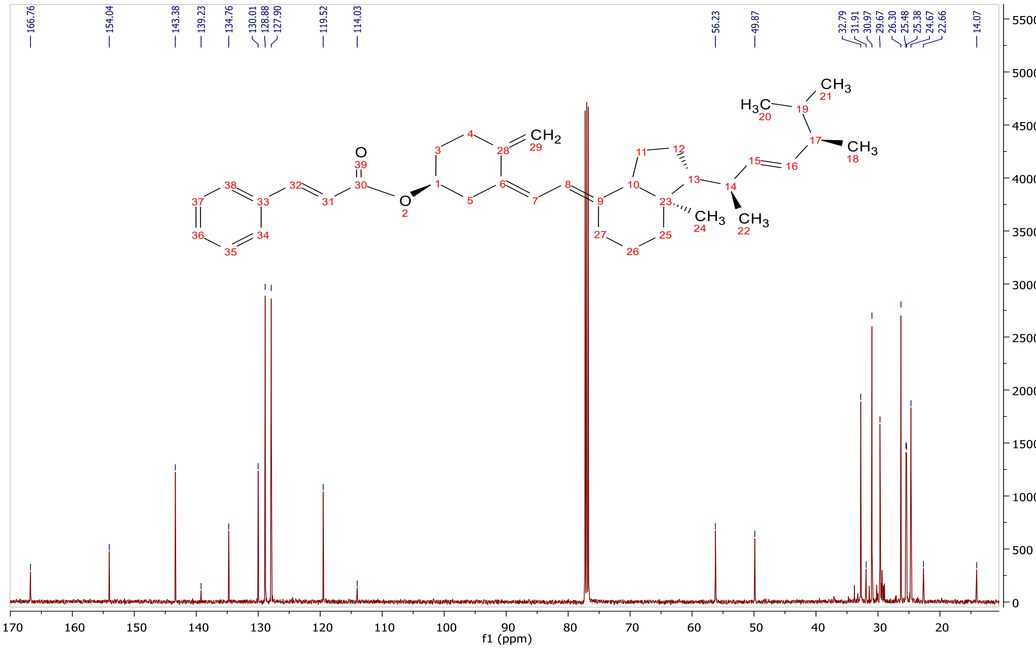

Figure S8c: ^{13}C-NMR spectra of **20**

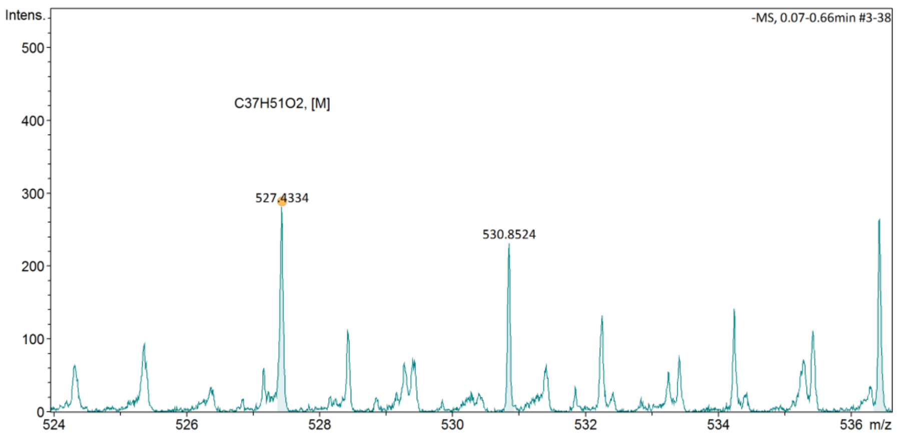

Figure S8d: HRMS spectrum of **20**

Table S1: SwissADME of the complex and hybrid molecules **4, 7, 11, 13, 15**

		**4**	**7**	**11**	**13**	**15**
Physicochemical Properties	Molar Refractivity	82.24	194.55	176.28	269.82	225.63
	TPSA	210.97 Å²	279.65 Å²	-231.44 Å²	251.67 Å²	294.66 Å²
Lipophilicity	Log $P_{o/w}$ (iLOGP)	0.00	-0.91	2.60	5.45	0.00
	Log $P_{o/w}$ (XLOGP3)	-6.91	-0.73	-0.34	6.66	1.69
	Log $P_{o/w}$ (WLOGP)	-1.24	0.88	1.27	6.83	2.20
	Log $P_{o/w}$ (MLOGP)	-3.11	-0.15	0.16	3.15	0.23
	Log $P_{o/w}$ (SILICOS-IT)	-4.27	0.36	1.71	5.77	1.28
	Consensus Log $P_{o/w}$	-3.19	-0.11	1.08	5.57	1.08
	Log *S* (ESOL)	1.28	-3.01	-2.76	-8.97	-5.14

Water Solubility	Class	Highly soluble	Soluble	Soluble	Poorly soluble	Moderately soluble
	Log *S* (Ali)	3.40	-4.67	-4.06	-11.75	-7.49
	Class	Highly soluble	Moderately soluble	Moderately soluble	Insoluble	Poorly soluble
Pharmacokinetic	GI absorption	Low	Low	Low	Low	Low
	BBB permeant	No	No	No	No	No
	P-gp substrate	Yes	Yes	Yes	Yes	Yes
	CYP1A2 inhibitor	No	No	No	No	No
	CYP2C19 inhibitor	No	No	No	No	No
	CYP2C9 inhibitor	No	No	No	No	No
	CYP2D6 inhibitor	No	No	No	No	No
	CYP3A4 inhibitor	No	Yes	Yes	Yes	No
	Log K_p (skin permeation)	-14.51 cm/s	-11.11 cm/s	-11.87 cm/s	-10.45 cm/s	-10.45 cm/s
Drug-likeness	Lipinski	No; 2 violations:	No; 2 violations:	No; 3 violations:	No; 3 violations:	No; 3 violations:

	MW>500, NHorOH>5	MW>500, NorO>10	MW>500, NorO>10, NHorOH>5	MW>500, NorO>10, NHorOH>5	MW>500, NorO>10, NHorOH>5
Ghose	No; 2 violations: MW>480, WLOGP<-0.4	No; 3 violations: MW>480, MR>130, #atoms>70	No; 3 violations: MW>480, MR>130, #atoms>70	No; 4 violations: MW>480, WLOGP>5.6, MR>130, #atoms>70	No; 3 violations: MW>480, MR>130, #atoms>70
Veber	No; 1 violation: TPSA>140	No; 2 violations: Rotors>10, TPSA>140	No; 2 violations: Rotors>10, TPSA>140	No; 2 violations: Rotors>10, TPSA>140	No; 2 violations: Rotors>10, TPSA>140
Egan	No; 1 violation: TPSA>131.6	No; 1 violation: TPSA>131.6	No; 1 violation: TPSA>131.6	No; 2 violations: WLOGP>5.88, TPSA>131.6	No; 1 violation: TPSA>131.6
Muegge	No; 3 violations: XLOGP3<-	No; 4 violations: MW>600,	No; 4 violations: MW>600,	No; 6 violations: MW>600,	No; 5 violations: MW>600,

	2, TPSA>15, H-don>5	TPSA>150, Rotors>15, H-acc>10	TPSA>150, Rotors>15, H-don>5	XLOGP3>5, TPSA>150, #rings>7, Rotors>15, H-don>5	TPSA>150, Rotors>15, H-acc>10, H-don>5
Bioavailability Score	0.17	0.17	0.11	0.11	0.11

Table S2: SwissADME of the complex and hybrid molecules **18, 19, and 20**

	**18**	**19**	**20**	**Pamidronate**	**Vitamin D2**	**MTX**	**Cisplatin**
				Physicochemical properties			
Molar Refractivity	190.87	218.08	168.72	42.42	129.37	118.40	21.16
TPSA (Å²)	43.37	89.52	23.60	180.93	20.23	210.54	24.72
			Lipophilicity				
Log $P_{o/w}$ (iLOGP)	-	-	-	-1.92	-	1.53	-

Log $P_{o/w}$ (XLOGP3)	-	-	-	-6.90	-	-1.85	-
Log $P_{o/w}$ (WLOGP)	10.80	10.72	9.80	-1.66	7.64	0.13	-
Log $P_{o/w}$ (MLOGP)	-	-	-	-3.41	-	-0.46	-
Log $P_{o/w}$ (SILICOS-IT)	-	-	-	-3.43	-	-0.66	-
Consensus Log $P_{o/w}$	-	-	-	-3.47	-	-0.26	-
Water solubility							
Log *S* (ESOL)	-	-	-	3.31	-	-1.99	-
Class				Highly soluble		Very soluble	
Log *S* (Ali)	-	-	-	3.91	-	-2.05	-
Class				Highly soluble		Soluble	
Pharmacokinetics							
GI absorption	-	-	-	Low	-	Low	-
BBB permeant				No		No	
P-gp substrate				Yes		Yes	
CYP1A2 inhibitor				No		No	
CYP2C19 inhibitor				No		No	
CYP2C9 inhibitor				No		No	
CYP2D6 inhibitor				No		No	
CYP3A4 inhibitor				No		No	

Log K_p (skin permeation) cm/s				-12.63		-10.39	
Drug Likeness							
Lipinski	-	-	-	Yes (1 violation)<br>NHorOH>5	-	Yes (1<br>violation)<br>NorO>10	-
Ghose	-	-	-	No (1 violation)<br>WLOGP<-0.4	-	No	-
Veber	-	-	-	No (1 violation)<br>TPSA>140	-	No (1<br>violation)<br>TPSA>140	-
Egan	-	-	-	No (1 violation)<br>TPSA>131.6	-	No (1<br>violation)<br>TPSA>131.6	-
Muegge	-	-	-	No (4<br>violations)<br>XLOGP3<-2,<br>TPSA>150,<br>#C<5, H-don>5	-	No (1<br>violation)<br>TPSA>150	-
Bioavailability Score	-	-	-	0.55	-	0.11	-

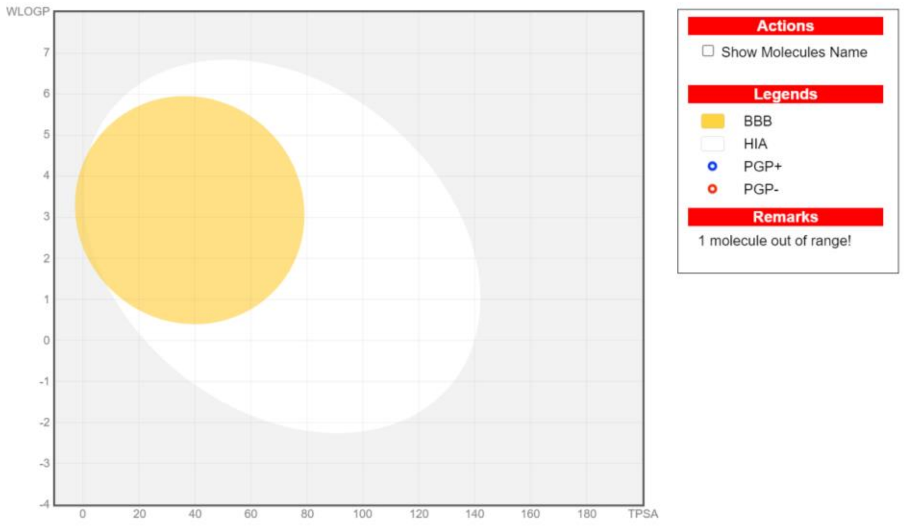

Figure S9: BOILED-Egg model of **4**

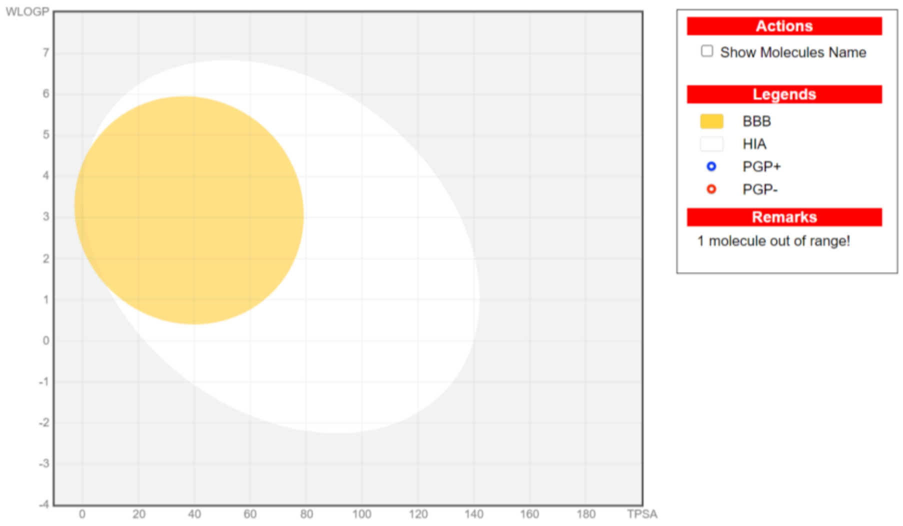

Figure S10: BOILED-Egg model of **7**

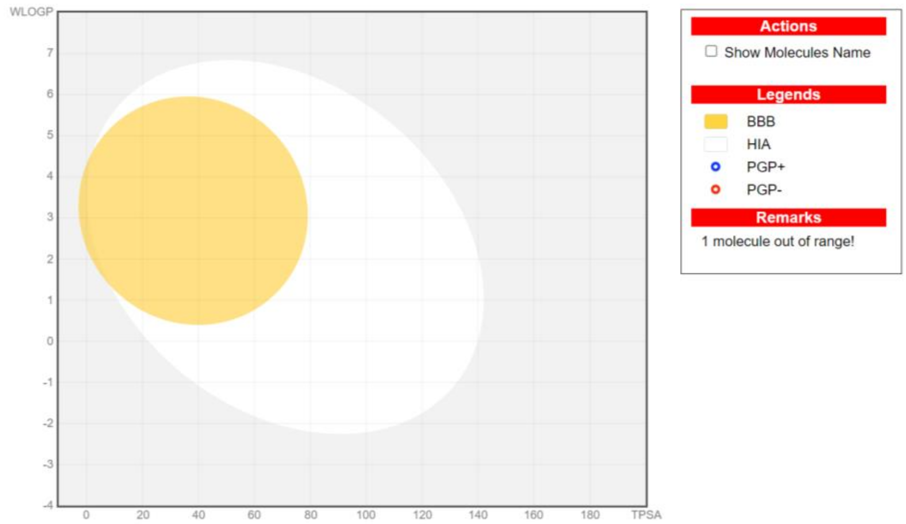

Figure S11: BOILED-Egg model of **11**

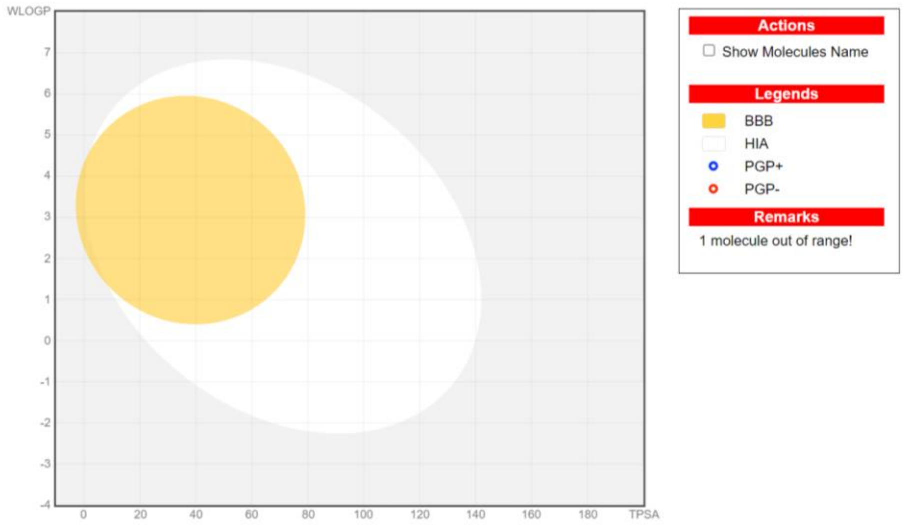

Figure S12: BOILED-Egg model of **13**

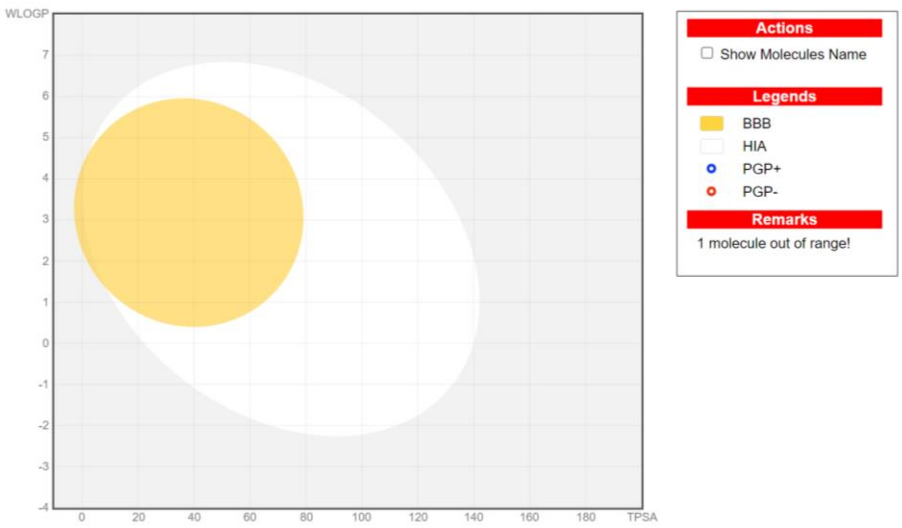

Figure S13: BOILED-Egg model of **15**

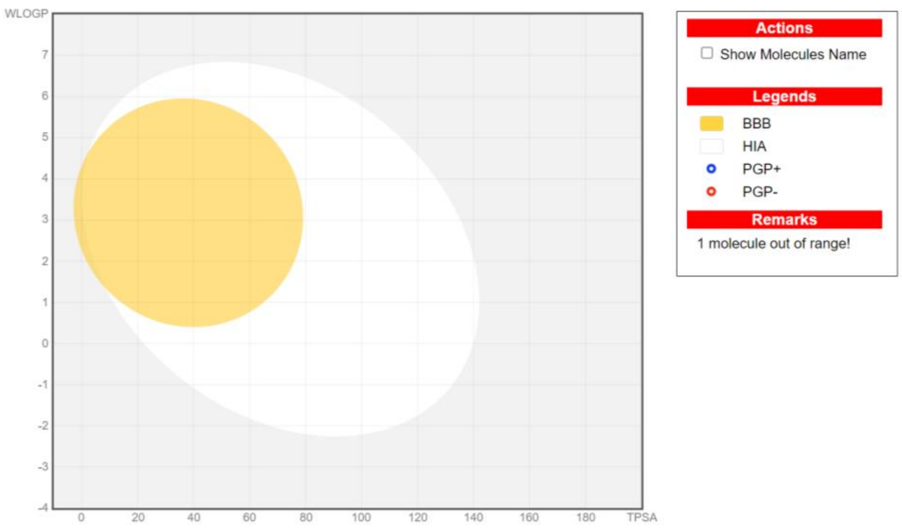

Figure S14: BOILED-Egg model of **18**

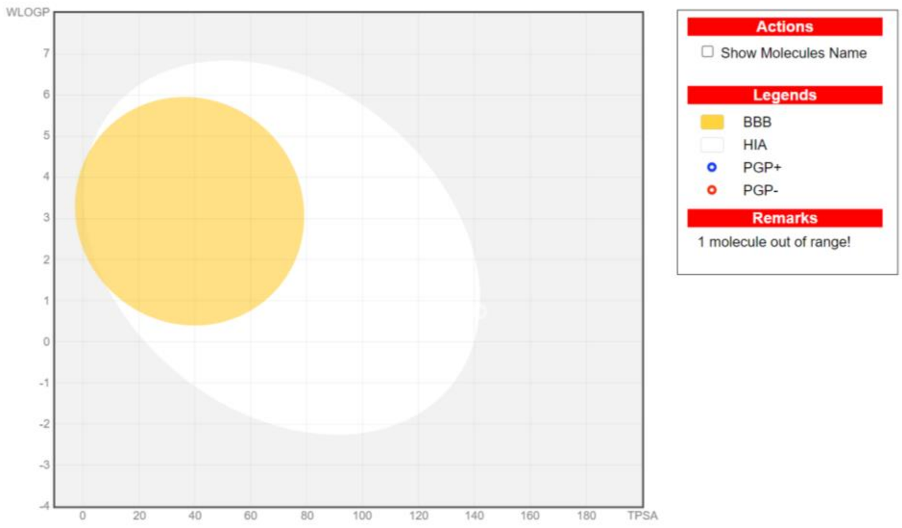

Figure S15: BOILED-Egg model of **19**

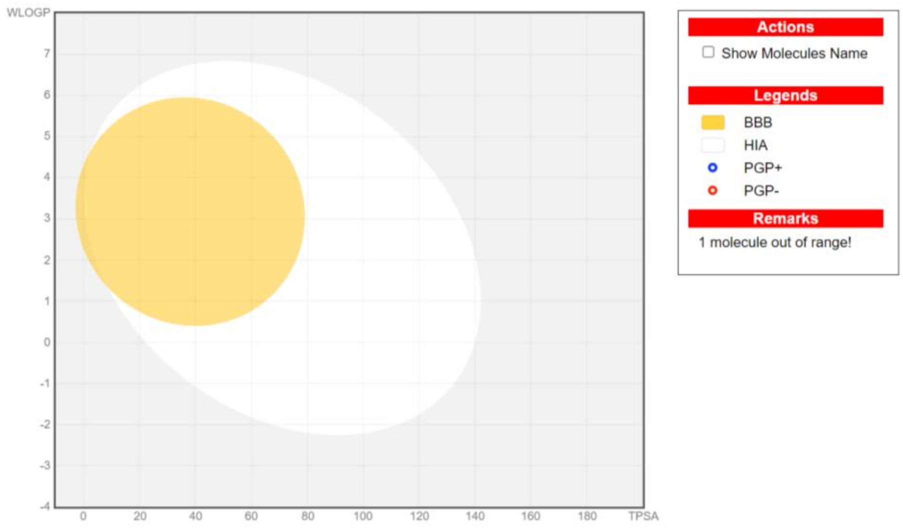

Figure S16: BOILED-Egg model of **20**

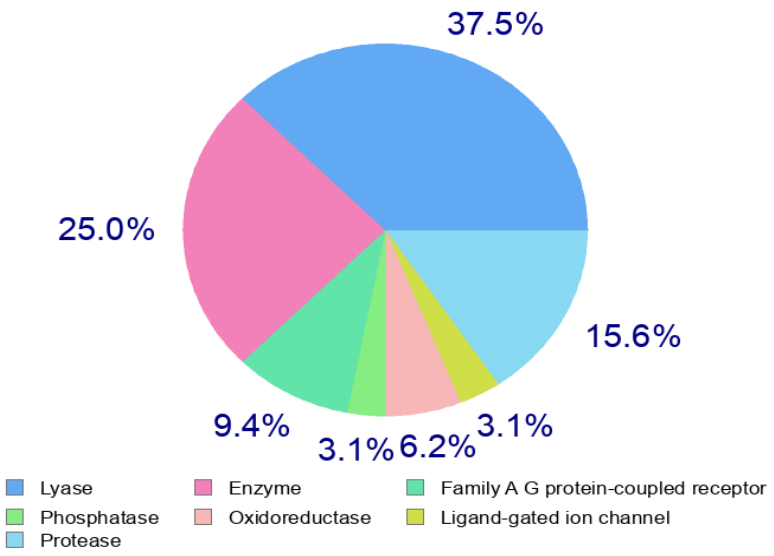

Figure S17: SWISS target prediction of **4**

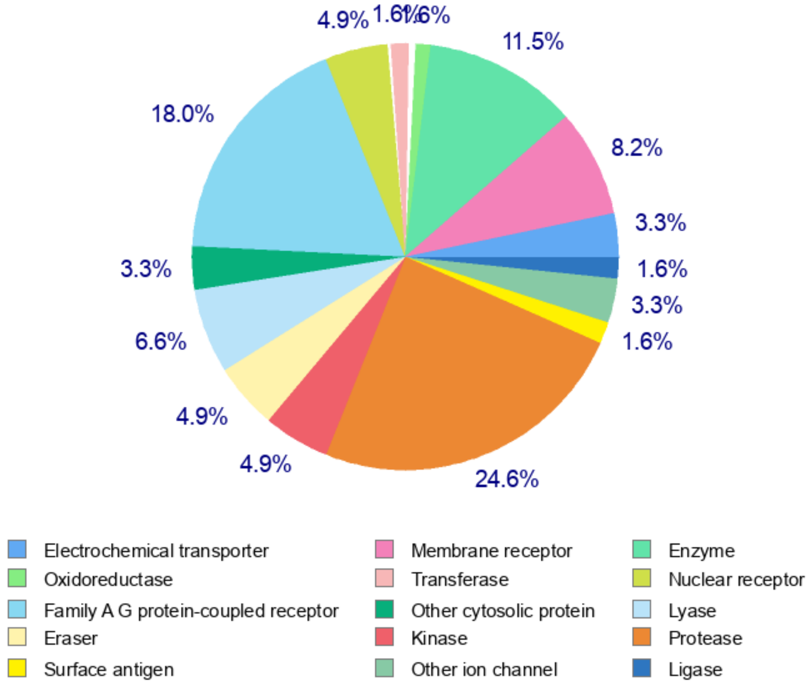

Figure S18: SWISS target prediction of **7**

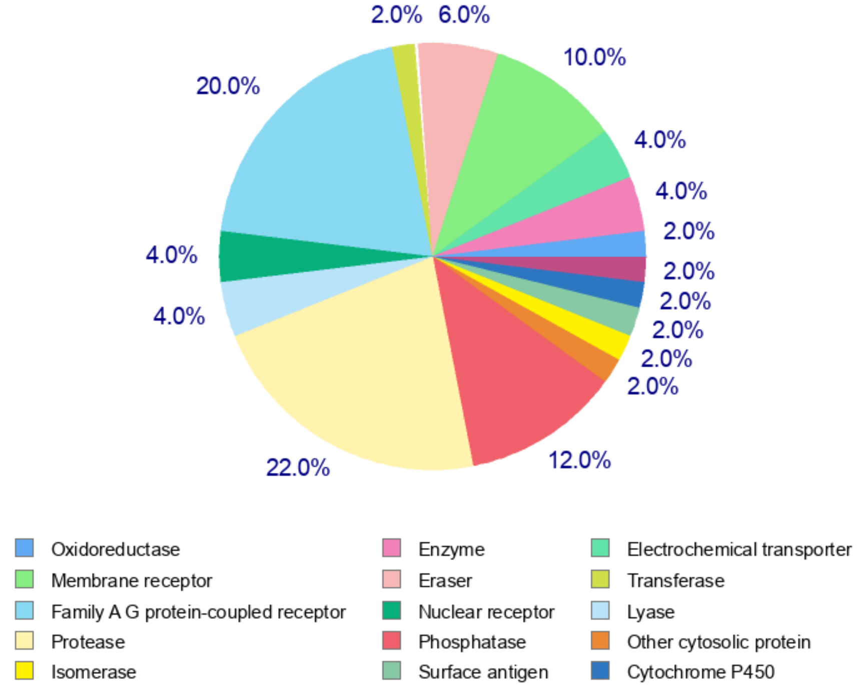

Figure S19: SWISS target prediction of **11**

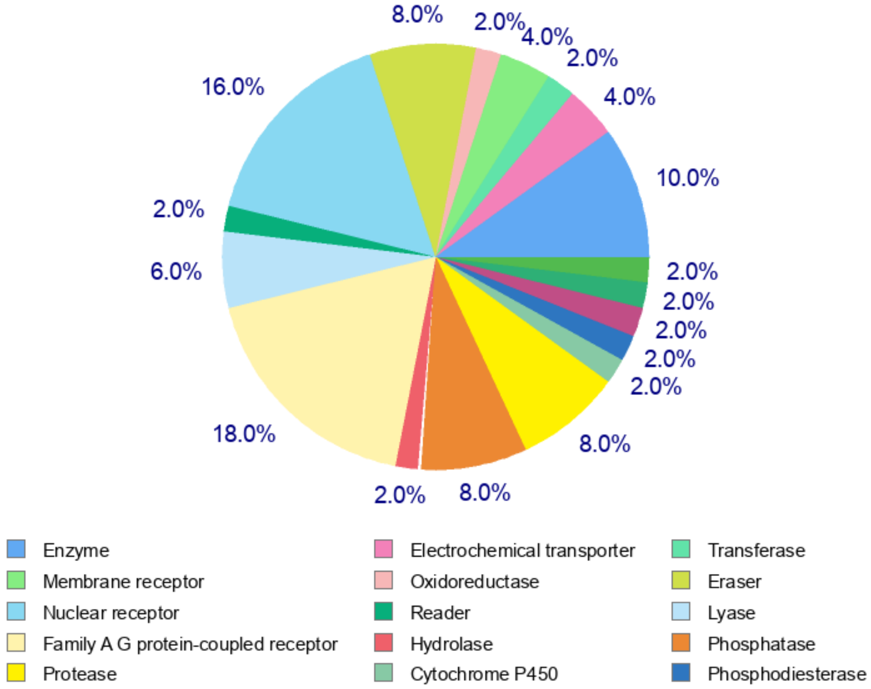

Figure S20: SWISS target prediction of **13**

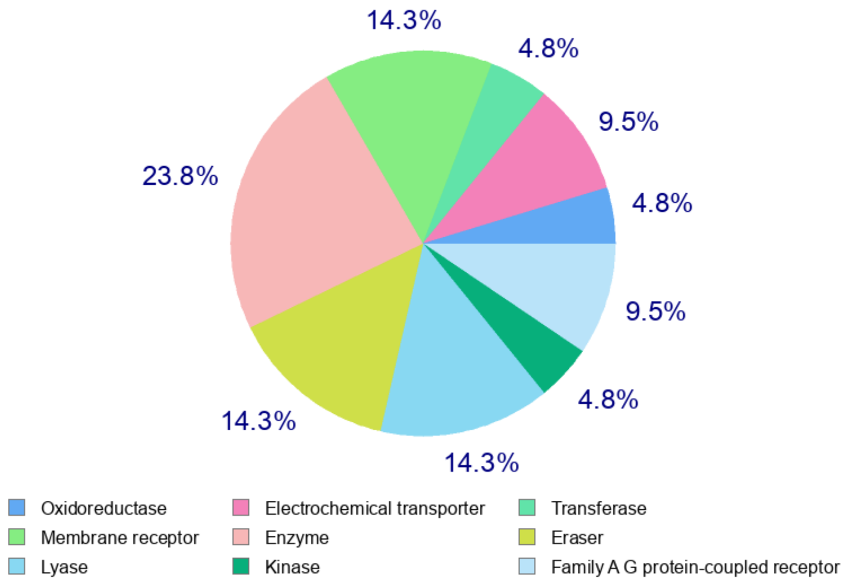

Figure S21: SWISS target prediction of **15**

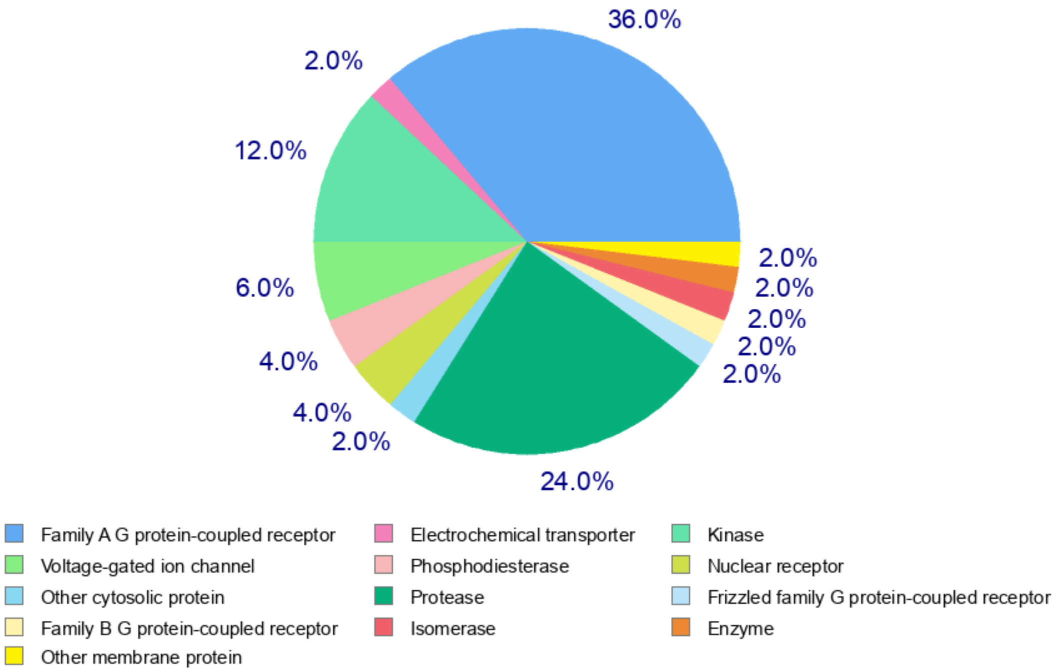

Figure S22: SWISS target prediction of **18**

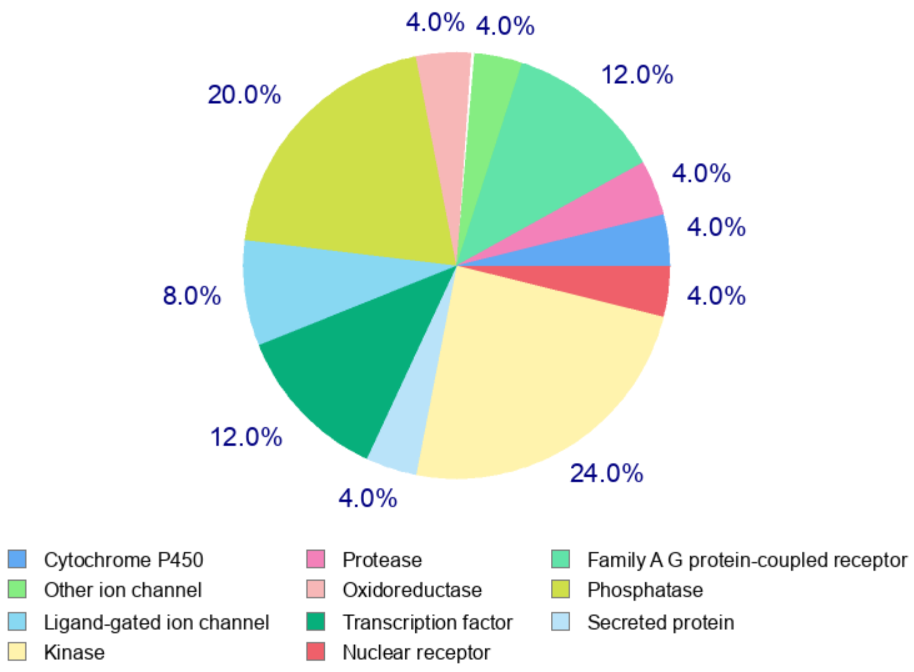

Figure S23: SWISS target prediction of **19**

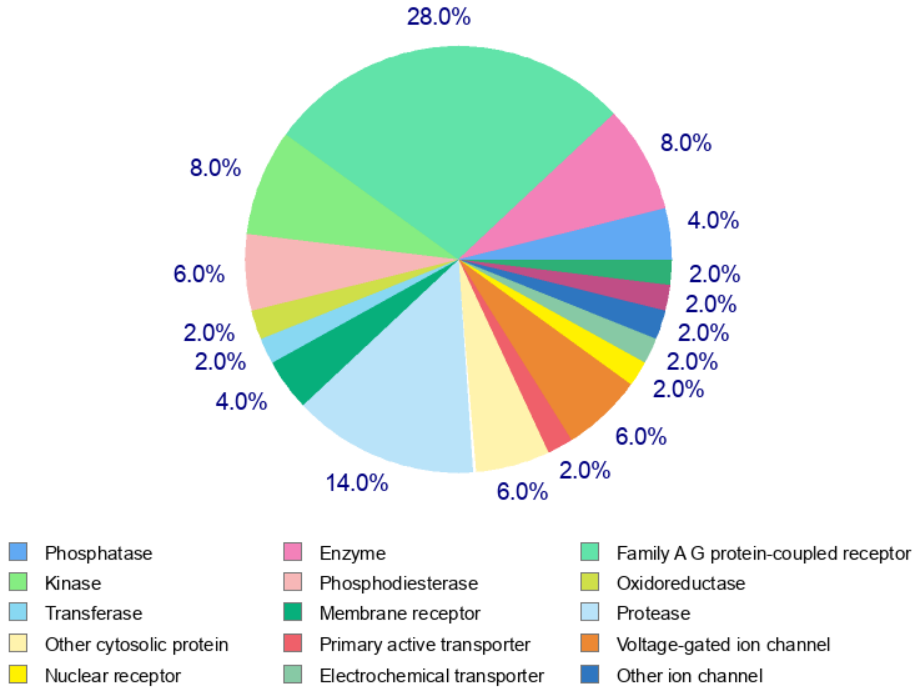

Figure S24: SWISS target prediction of **20**

Table S3: Organ toxicity and toxicological endpoints predicted activity calculated using the ProTox-II web server

Classification					
**Compounds**	**Organ Toxicity**	**Toxicity Endpoint**			
	Hepatotoxicity	Carcinogenicity	Immunotoxicity	Mutagenicity	Cytotoxicity
	Prediction and Probability				
**4**	Inactive (0.77)	Inactive (0.57)	Inactive (0.99)	Inactive (0.53)	Inactive (0.60)
**7**	Inactive (0.69)	**Active** (0.51)	**Active** (0.64)	Inactive (0.59)	Inactive (0.53)
**11**	Inactive (0.67)	**Active** (0.50)	**Active** (0.80)	Inactive (0.80)	Inactive (0.72)
**13**	Inactive (0.55)	**Active** (0.54)	**Active** (0.99	Inactive (0.69)	Inactive (0.)
**15**	Inactive (0.66)	Inactive (0.54)	**Active** (0.99)	Inactive (0.62)	Inactive (0.76)
**18**	Inactive (0.71)	Inactive (0.53)	**Active** (0.99)	Inactive (0.96)	Inactive (0.62)
**19**	Inactive (0.80)	Inactive (0.59)	**Active** (0.99)	Inactive (0.63)	Inactive (0.56)
**20**	Inactive (0.57)	Inactive (0.55)	**Active** (0.99)	Inactive (0.97)	Inactive (0.79)
**Vitamin D2**	-	-	-	-	-
**Pamidronate**	Inactive (0.97)	Inactive (0.66)	Inactive (0.99)	Inactive (0.67)	Inactive (0.62)
**Methotrexate**	**Active (0.69)**	Inactive (0.52)	Inactive (0.98)	Inactive (0.93)	Inactive (0.65)
**Cisplatin**	Inactive (0.73)	Inactive (0.71)	Inactive (0.99)	Inactive (0.60)	Inactive (0.68)

Table S4: Rat Toxicity Prediction using GUSAR

Compounds	Rat Oral LD50 (mg/kg)/ Toxicity Classification	Rat IP LD50 (mg/kg)/ Toxicity Classification	Rat IV LD50 (mg/kg)/ Toxicity Classification	Rat SC LD50 (mg/kg)/ Toxicity Classification
**4**	1847.000/Class 4[a]	516.200/Class 5 [a]	89.100/Class 4 [a]	250.500/Class 4 [a]
**7**	194.400/Class 4[a]	723.000/Class 5[b]	262.1000/Class 3[b]	467.000/Class 4[a]
**11**	1338.000/Class 4[a]	1305.000/Class 5[a]	299.000/Class 4[a]	758.300/Class 4[a]
**13**	2350.000/Class 5[a]	808.600/Class 5[a]	47.100/Class 4[a]	292,900/Class 4[a]
**15**	955.000/Class 4[a]	312.800/Class 4[a]	77.754/Class 4[a]	311.900/Class 4[a]
**18**	65.900/Class 3[a]	766.400/Class 5[a]	9.831/Class 3[a]	53.440/Class 3[3]
**19**	111.500/Class 3[a]	882.100/Class 5[a]	8.517/Class 3[a]	262.500/Class 4[a]
**20**	245.700/Class 3[a]	258.500/Class 4[b]	5.639/Class 2[a]	52.390/Class 3[b]
**Pamidronate**	4959.000/Class 5 [a]	459.7000/Class 4 [a]	133.900/Class 4 [a]	128.6000/Class 3[a]
**Vitamin D2**	13.910/Class 2[a]	293.500/Class 4[a]	0.764/Class 2[b]	12.670/Class 2[b]
**Methotrexate**	592.700/Class 4[b]	686.700/Class 5[a]	634,100/Class 5[a]	1033.000/ Class 5[a]
**Cisplatin**	66.620/ Class 3[b]	116.200/Class 4[a]	94.220/Class 4[a]	53.070/ Class 3[a]

Route of administration: P - Intraperitoneal; IV - Intravenous; Oral - Oral; SC - Subcutaneous.

Applicability Domain: [a]Compound falls in the applicability domain of models; [b]Compound is out of the applicability domain of models.

Toxicity classes are defined according to the globally harmonized system of classification of labelling of chemicals (GHS). LD50 values are given in [mg/kg]:

- Class I: fatal if swallowed ($LD_{50} \leq 5$)
- Class II: fatal if swallowed ($5 < LD_{50} \leq 50$)
- Class III: toxic if swallowed ($50 < LD_{50} \leq 300$)
- Class IV: harmful if swallowed ($300 < LD_{50} \leq 2000$)
- Class V: may be harmful if swallowed ($2000 < LD_{50} \leq 5000$)
- Class VI: non-toxic ($LD_{50} > 5000$)

Table S5: Environmental Toxicity using GUSAR

**Compounds**	**Activity**			
	Bioaccumulation factor Log10 (BCF)	Daphnia magna LC_{50} - Log10(mol/L)	Fathead Minnow LC_{50} Log10(mmol/L)	Tetrahymena pyriformis IGC_{50} - Log10(mol/L)
**4**	-0.042[a]	5.239[a]	-1.776[a]	0.449[a]
**7**	-1.146[a]	6.761[a]	-6.072[b]	1.677[a]
**11**	-0.861[a]	6.446[a]	-5.496[b]	1.647[a]
**13**	-4,791[b]	6.284[b]	-8.481[b]	1.726[a]
**15**	-2,457[b]	7.075[a]	-7.598[b]	2.117[a]

**18**	-0,183[b]	7.472[a]	-8.891[b]	1.627[a]
**19**	-1,247[b]	6.977[a]	-8.680[b]	2.498[a]
**20**	0.945[a]	7.450[a]	-7,819[a]	3.142[a]
**Pamidronate**	0.239[a]	4.670[a]	0.091[a]	-0.063[a]
**Vitamin D2**	2.030[a]	6.279[a]	-5.419[a]	2.462[a]
**Methotrexate**	-0.620[a]	5.149[a]	-2.039[a]	1.233[a]
**Cisplatin**	0.684[a]	3.222[a]	-0,593[a]	0.654[a]

Applicability Domain: [a]Compound falls in the applicability domain of models; [b]Compound is out of the applicability domain of models.

Table S6: hERG-predictions from Pred-hERG

Compounds	Binary Prediction	Confiability %	AD	Multiclass prediction	Confiability %	AD	Prediction (pIC_{50})
**4**	Non-blocker	98.68	Inside AD	Non-Blocker	36.1	Inside AD	4.95
**7**	Non-blocker	52.1	Inside AD	Non-blocker	31.9	Inside AD	5.429
**11**	Non-blocker	52.92	Inside AD	Moderate blocker	31.98	Inside AD	5.404

**13**	Non-blocker	86.41	Inside AD	Moderate blocker	32.7	Inside AD	5.599
**15**	Non-blocker	61.99	Inside AD	Weak blocker	31.3	Inside AD	5.599
**18**	Blocker	61.57	Outside AD	Weak blocker	32.81	Outside AD	5.37
**19**	Blocker	62.73	Inside AD	Weak blocker	29.54	Inside AD	5.469
**20**	Blocker	73.84	Inside AD	Weak blocker	31.4	Inside AD	5.351
**Pamidronate**	Non-blocker	99.96	Outside AD	Non-blocker	53.4	Outside AD	4.672
**Vitamin D2**	Non-blocker	73.43	Outside AD	Weak blocker	39.6	Outside AD	5.057
**MTX**	Non-blocker	79.35	Inside AD	Moderate blocker	33.4	Inside AD	5.374
**Cisplatin**	Non blocker	99.85	Outside AD	Non-blocker	47.1	Outside AD	4.904

AD-Applicability Domain

Table S7: The fragment Contribution Maps for the Regression Model

Compounds	Fragment Contribution Maps for the Regression Model	Fragment Contribution Maps and Explainable AI (XAI) for the Binary Model
**4**		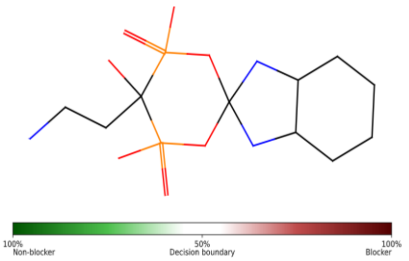
**7**	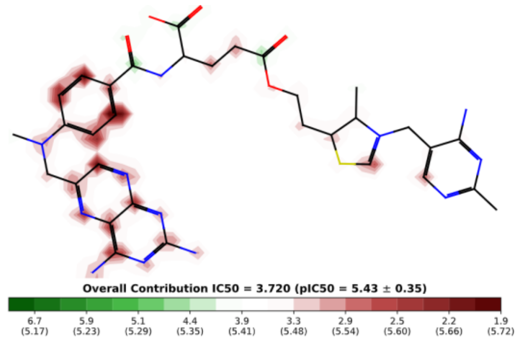	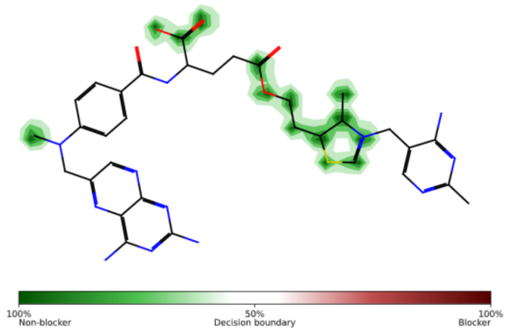
**11**	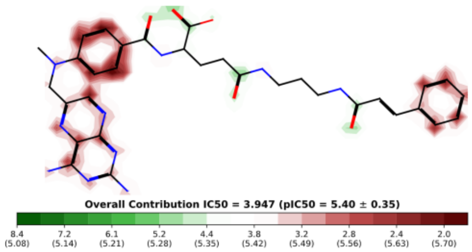	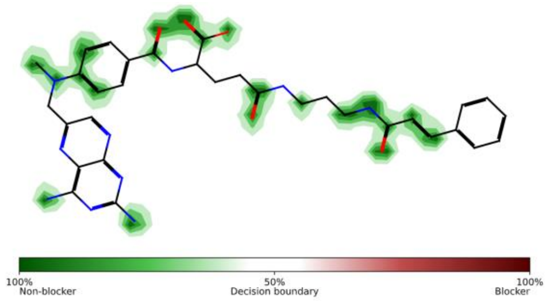
**13**		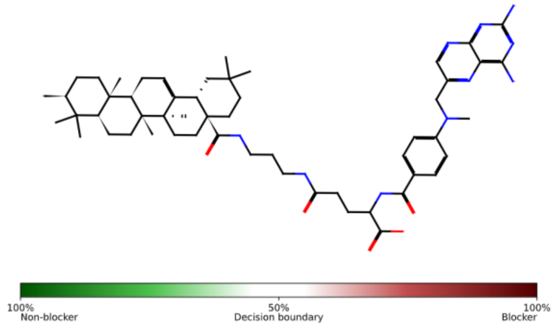

15

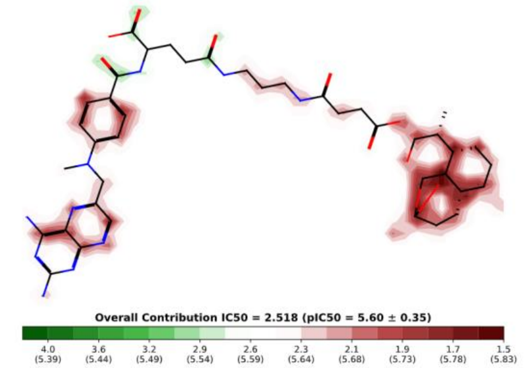

Overall Contribution IC50 = 2.518 (pIC50 = 5.60 ± 0.35)
4.0 (5.39)
3.6 (5.44)
3.2 (5.49)
2.9 (5.54)
2.6 (5.59)
2.3 (5.64)
2.1 (5.68)
1.9 (5.73)
1.7 (5.78)
1.5 (5.83)

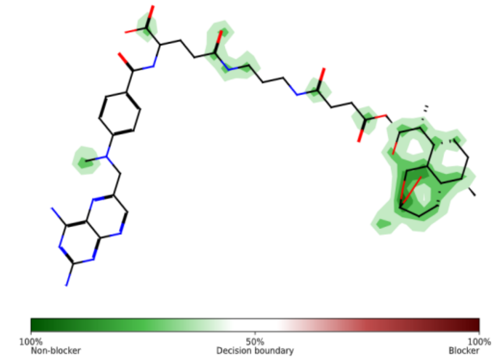

100% Non-blocker
50% Decision boundary
100% Blocker

19

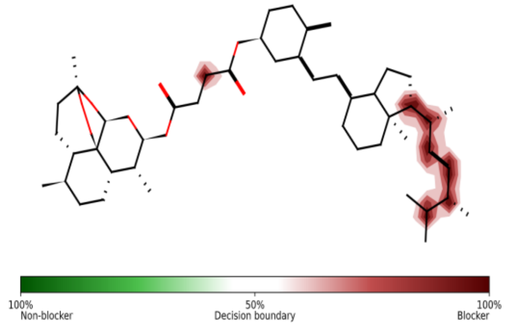

100% Non-blocker
50% Decision boundary
100% Blocker

20

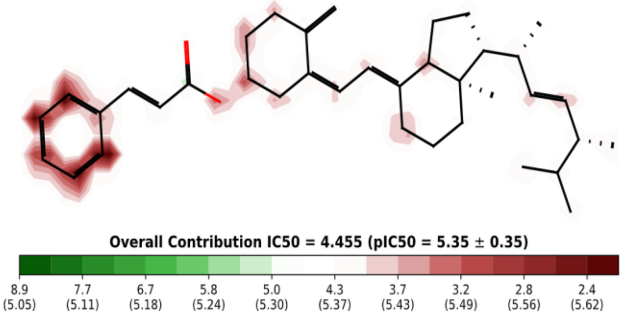

Overall Contribution IC50 = 4.455 (pIC50 = 5.35 ± 0.35)
8.9 (5.05)
7.7 (5.11)
6.7 (5.18)
5.8 (5.24)
5.0 (5.30)
4.3 (5.37)
3.7 (5.43)
3.2 (5.49)
2.8 (5.56)
2.4 (5.62)

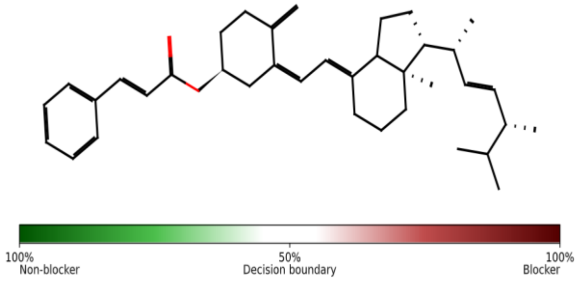

100% Non-blocker
50% Decision boundary
100% Blocker

MTX

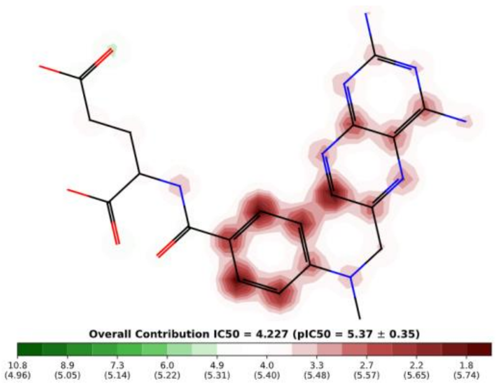

Overall Contribution IC50 = 4.227 (pIC50 = 5.37 ± 0.35)
10.8 (4.96)
8.9 (5.05)
7.3 (5.14)
6.0 (5.22)
4.9 (5.31)
4.0 (5.40)
3.3 (5.48)
2.7 (5.57)
2.2 (5.65)
1.8 (5.74)

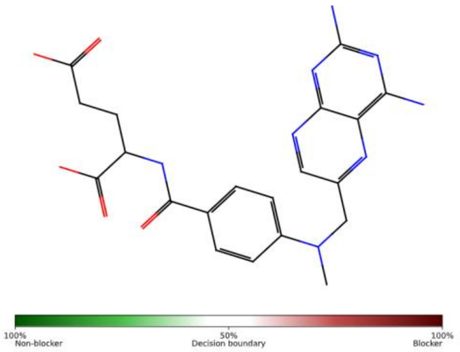

100% Non-blocker
50% Decision boundary
100% Blocker

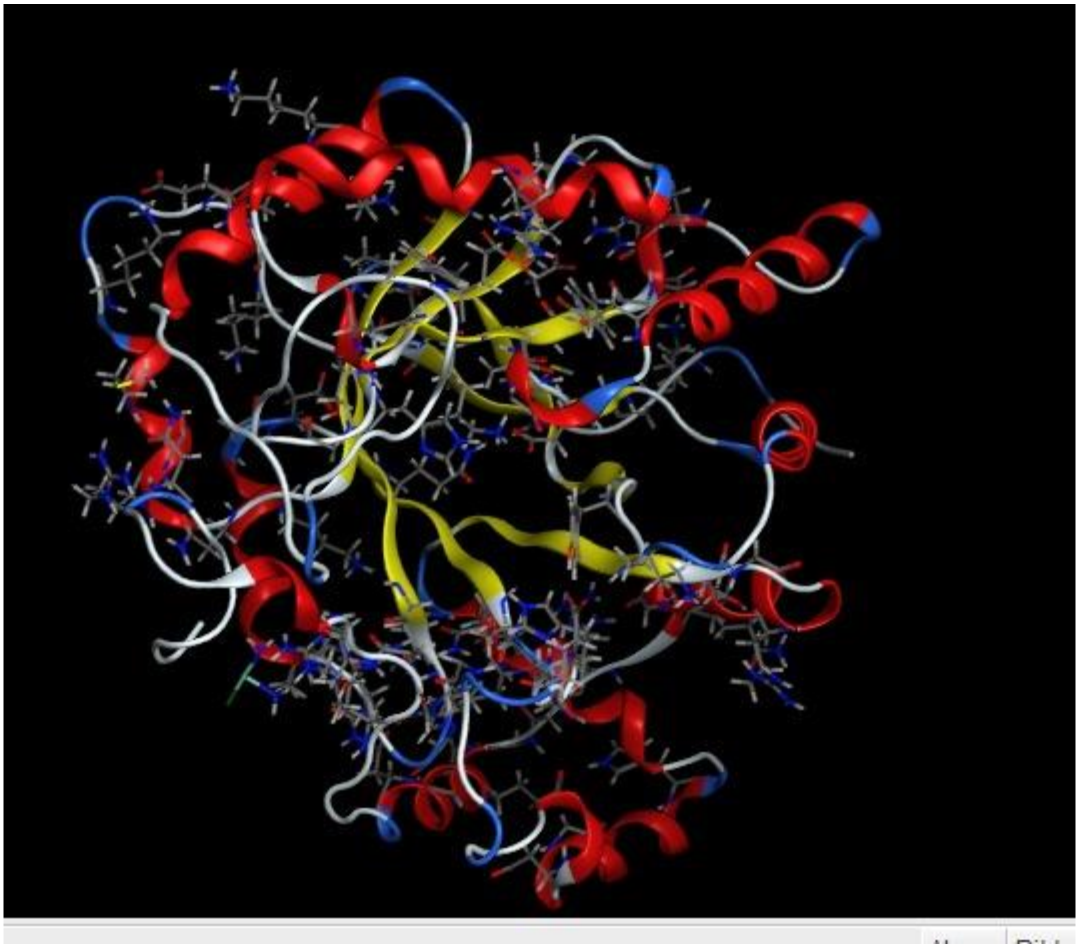

Figure S25: Representative 3D structure of the receptor: Human 3 alpha-hydroxysteroid dehydrogenase type 3.
